# GRASPing experience-dependent protein expression signatures enriched for hippocampal engram cell synapses

**DOI:** 10.1126/sciadv.adv3557

**Published:** 2026-05-15

**Authors:** Biswajit Moharana, Panthea Nemat, Renee M. Pullen, Anna Gradl, Marijn Schipper, Jeanne E. Savage, Remco V. Klaassen, Rolinka J. van der Loo, Cora H. Chadick, Frank Koopmans, Yvonne Gouwenberg, Juan J. Garcia Vallejo, Michel C. van den Oever, August B. Smit, Priyanka Rao-Ruiz

**Affiliations:** ^1^ Dept. of Molecular and Cellular Neurobiology, Center for Neurogenomics and Cognitive Research, Amsterdam Neuroscience, Vrije Universiteit Amsterdam, Amsterdam, Netherlands.; ^2^ Dept. of Complex Trait Genetics, Center for Neurogenomics and Cognitive Research, Amsterdam Neuroscience, Vrije Universiteit Amsterdam, Amsterdam, Netherlands.; ^3^ Microscopy and Cytometry Core Facility, Amsterdam UMC–Location VUMC, Amsterdam, Netherlands.; ^4^ Thermo Fisher Scientific, Greater Seattle Area, Seattle, WA, USA.

## Abstract

Enhanced synaptic wiring onto sparsely distributed memory engram cells supports contextual memory storage and recall, with negative-valence memories exhibiting greater salience. Protein-level adaptations of input-specific synaptic connectivity onto hippocampal CA1 engram cells, however, remain largely unexplored. By combining spatiotemporally restricted synapse labeling, sorting, and mass spectrometry, we generated a discovery-oriented proteomic dataset from samples enriched for CA1 engram cell synapses receiving CA3 input 72 hours after neutral context exploration or aversive contextual fear conditioning. Differential analysis relative to an unlabeled comparator identified protein expression signatures associated with synapse structure, efficacy, and strength in CA1 engram cell synapses. Aversive learning induced predominantly postsynaptic protein expression patterns, whereas neutral context skewed toward presynaptic changes. This differential engram cell synapse proteome was enriched for genes linked to cognitive genetic traits and related disorders. Together, these data provide a hypothesis-generating resource for investigations into the molecular basis of contextual memory at the level of the synapse.

## Teaser

Toward identifying the protein landscape of memory-encoding hippocampal synapses.

## INTRODUCTION

Memory consolidation and its subsequent maintenance over time require experience- and time-dependent modifications in sparsely distributed neurons, collectively known as engram cells, activated at the time of learning (*1*). Contextual information, an essential component of episodic memory, is encoded by coherently activated engram cells within the hippocampus (*2*–*7*). The salience of the encoded context increases when associated with a negative valence, but whether and how this is represented within an engram are largely unknown.

Hippocampal CA1 cells receive a large majority of their input from CA3 via Schaffer collaterals and commissural fibers projecting to the stratum oriens and stratum radiatum of both hemispheres (*8*, *9*). CA1 engram cells are responsible for the consolidation, maintenance, and retrieval of recent contextual memories (*6*). Learning induces AMPA receptor (AMPAR)–mediated augmentation of CA1 engram cell postsynaptic responses (*10*), spinogenesis, and the modification of existing synapses between preconnected CA3 and CA1 cells that are recruited into the engram (*11*). This learning-dependent physiological and structural synaptic plasticity facilitates efficient reactivation of engram cells to natural cues enabling memory recall (*12*, *13*) and likely involves changes in the molecular composition of engram cell synapses (*12*, *14*). However, the identity of these protein-level adaptations remains largely unknown.

A recently developed method to label input-specific synapses in vivo, enhanced Green Fluorescent Protein Reconstitution Across Synaptic Partners (eGRASP), has enabled morphometric analysis of memory-encoding synapses (*10*, *11*, *15*, *16*). While this reveals structural modifications, the protein architecture of engram cell synapses—crucial for understanding the molecular mechanisms supporting contextual memories—is unexplored because of difficulties in quantifying proteins in these sparsely distributed synapses. As a first step toward addressing this, we harvested two populations of hippocampal synaptosomes, one enriched for spatiotemporally restricted and activity-dependent eGRASP-tagged dorsal CA3-CA1 engram cell synapses and the other for the bulk of unlabeled dorsal hippocampal synapses, 72 hours after either aversive contextual fear conditioning (CFC) or neutral context exploration (CE). The proteome of these samples was then analyzed using an in-house optimized workflow for small-scale sample processing and in-depth, quantitative, and sensitive mass spectrometry (MS) (*17*, *18*). Our data show that a distinct set of proteins that overlap partially in function, but not in identity, to previously characterized synaptic plasticity modulators, is enriched or depleted in labeled CA1 engram cell synapses when compared to unlabeled heterogeneous comparators. In addition, we demonstrate that these differentially expressed engram cell synaptic protein signatures associate with cognitive phenotypes and disorders, highlighting their potential clinical relevance. Furthermore, robust regression analysis identified postsynaptic protein expression patterns as more dominant for salient negative contextual memories when compared to neutral contexts. Last, we validated the enrichment of selected proteins using flow cytometry (*19*) and characterized the higher expression and localization of Grm5, the most significantly enriched and dominant CFC protein, in the smaller subset of synapses between CA3 engram and CA1 engram cells coactivated during learning.

## RESULTS

### Viral-TRAP + eGRASP enables activity-dependent tagging and morphometry of CA3 synapses onto CA1 engram cells

**Fig. 1.**
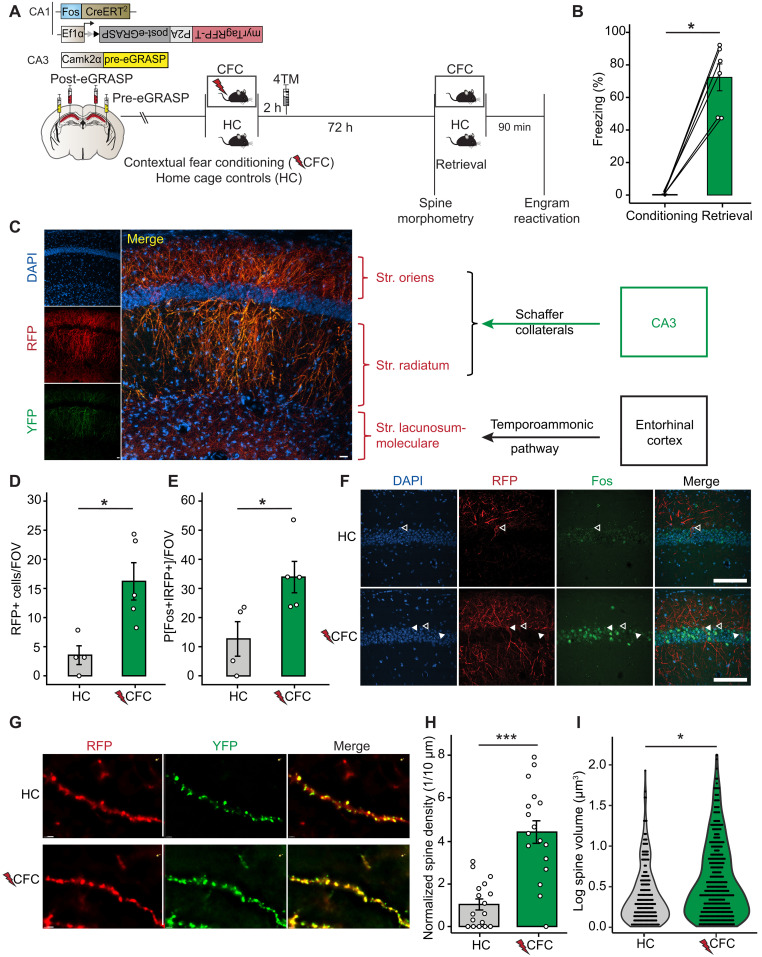
Viral-TRAP + eGRASP enables tagging and morphometry of CA3 synapses onto CA1 engram cells. (**A**) Experimental setup. Mice injected with eGRASP constructs remained in their HC or underwent CFC and were injected with 4TM 2 hours (h) later. Spine morphometry or cellular analysis 72 hours after CFC or memory retrieval. (**B**) CFC mice (*n* = 6) exhibit freezing behavior during memory retrieval. Paired-samples Wilcoxon signed-rank test, *P = 0.036. Data are presented as the means ± SEM. Each data point equals one mouse. (**C**) Representative image of CA1 RFP and YFP expression. Scale bars, 100 μm. (**D**) More RFP+ cells after CFC than HC. Wilcoxon rank-sum test, HC: *n* = 4; CFC: *n* = 5; *P = 0.020. Data are presented as the means ± SEM. Each data point equals one mouse. FOV, field of view. (**E**) Higher percentage of RFP+ cells colocalizing with Fos after CFC (CFC: *n* = 5; HC: *n* = 4). Independent samples *t* test, *P = 0.035. Data are presented as the means ± SEM. Each data point equals one mouse. (**F**) Representative images of RFP and Fos coexpression (HC: upper panel; CFC: lower panel). Filled arrows: RFP+/Fos+; outlined arrows: RFP+/Fos−. Scale bars, 100 μm. (**G**) Representative images of RFP+YFP+ CA1 dendrites and synapses (HC: upper panel; CFC: lower panel). Scale bars, 2 μm. (**H**) Spine density on reconstructed CA1 dendrites higher after CFC than HC (main effect condition: *b* = 3.60, *t* = 7.68, ***P < 0.001; *n*_slice_ = 15, *N*_dendrite_ = 34; clustering according to slice). Bar plot of means ± SEM. Each data point equals one dendrite. (**I**) Larger eGRASP+ spine volume after CFC than HC (main effect condition: *b* = −0.92, *t* = −2.25, *P = 0.025; *n*_slice_ = 15, *n*_dendrite_ = 77, *N*_spine_ = 595; clustering according to dendrite nested in slice on log-transformed values). Violin plot of data distribution. Each data point equals one spine. DAPI (blue), RFP (red), and YFP/Fos (green).

eGRASP facilitates input-specific synapse labeling by reconstitution of pre- and postsynaptic fluorescent protein fragments across the 20-nm mammalian synaptic cleft (*10*, *20*, *21*). To drive post-eGRASP expression to CA1 engram cells, we combined it with our previously validated viral-based Targeted Recombination in Active Populations (TRAP) (*22*). TRAP facilitates the expression of a second Cre-dependent vector in activated (*Fos* expressing) neurons in a 4-hydroxytamoxifen (4TM)–controlled manner (*22*–*24*). To label ipsi- and contralateral dorsal CA3 input onto dorsal CA1 engram cells (i.e., total CA3 input onto CA1 engram cells, CA3^T^-CA1^E^ synapses), we injected mice bilaterally with a *CaMKII*α-driven yellow pre-eGRASP in CA3 and a mixture of viral-TRAP and Cre-dependent post-eGRASP with membrane-targeted myristoylated red fluorescent protein (RFP) into CA1 (Fig. 1A). Mice either remained in their home cage (HC) or underwent CFC followed by systemic 4TM treatment to induce recombination and expression of the myrTagRFP-T-post-eGRASP construct (Fig. 1A). The combination of these viral constructs and synaptic expression of eGRASP did not interfere with learning (*11*), evidenced by robust freezing observed in a memory retrieval test 72 hours after CFC (freezing prior to foot shock delivery: 0.34 ± 0.34%; freezing during retrieval: 72.34 ± 8.25%; Fig. 1B).

While myristoylated RFP was expressed along the membrane of labeled CA1 neurons, yellow fluorescent protein (YFP) was expressed in the stratum radiatum and stratum oriens of CA1 that receive synaptic input from CA3 neurons expressing the pre-eGRASP construct (Fig. 1C and fig. S1A). We observed minimal YFP reconstitution in the stratum lacunosum moleculare where axons originating from the entorhinal cortex terminate (Fig. 1C and fig. S1A) or within CA1 cell somata, thereby reducing the likelihood of pre- and post-eGRASP expression co-occurring within the same neuron (fig. S1, B and C) (*15*). Using viral-TRAP–driven myristoylated RFP as a marker for engram cells and endogenous Fos as a proxy of neuronal activity during memory retrieval (*22*), we found that when compared to HC controls, CFC induced a significantly higher number of labeled cells that were also reactivated upon memory retrieval (RFP+ cells—HC: 3.54 ± 1.61; CFC: 16.22 ± 3.21; Fos+ of RFP+—HC: 10.58 ± 4.95%; CFC: 28.28 ± 4.49%; Fig. 1, D to F). Morphometric analysis of RFP+YFP+ spines on reconstructed RFP-expressing CA1 engram dendrites showed that learning increased spine density and volume 72 hours after CFC (spine density—HC: 1.04 ± 0.25; CFC: 4.4 ± 0.53; log-transformed spine volume—HC: 0.40 ± 0.03; CFC: 0.56 ± 0.02; Fig. 1, G to I, and fig. S1D). Together, these findings indicate that the combination of viral-TRAP and eGRASP enables activity- and 4TM-dependent labeling of CA3 synapses onto a sparse population of CA1 neurons and that these cells display enhanced synapse structural connectivity 72 hours after learning. Thus, we next asked which protein expression signatures are associated with this augmented state at the level of the engram cell synapse.

### Isolation and proteomic analysis of eGRASP+ and eGRASP− synaptosome–enriched samples

**Fig. 2.**
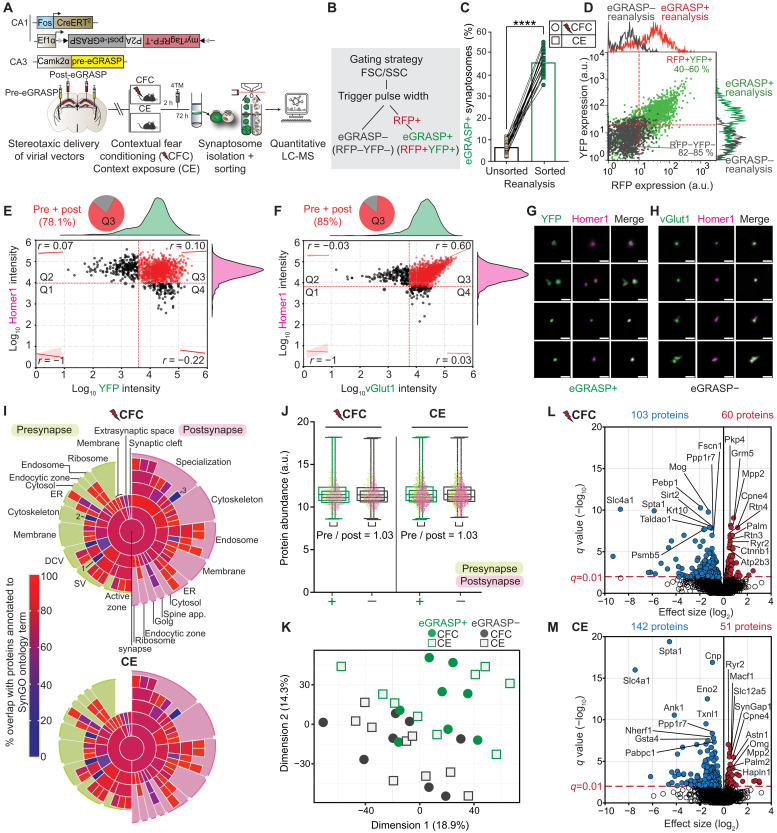
Isolation and proteomic analysis of eGRASP+ and eGRASP− synaptosome–enriched samples. (**A**) Experimental setup. Synaptosome isolation from dorsal hippocampi 72 hours after CFC or CE for sorting and MS. (**B**) Sort gate hierarchy. (**C**) Percentage of gated eGRASP+ events (CFC, *n* = 11; CE, *n* = 10). Two-tailed paired *t* test, ****P < 0.0001. Data are presented as the means ± SEM. a.u., arbitrary units. (**D**) Representative ungated fluorescence profile dot plots of sorted, reanalyzed eGRASP+ (RFP+YFP+, green) and eGRASP− (RFP−YFP−, gray) samples. Marginal histograms display density distributions (RFP and YFP expression). (**E** and **F**) Correlation and colocalization of synaptic markers, (E) eGRASP+ and (F) eGRASP−. Scatterplot correlation between (E) YFP (*x* axis), (F) vGlut1 (*x* axis), and Homer1 [*y* axis; (E) and (F)]. Q3 represents the highest correlation coefficients [(E): *r* = 0.10; (F): *r* = 0.60]. Pie charts of Q3 proportions containing both pre- and postsynaptic elements. Marginal histograms display density distributions per channel (CFC: *n* = 5; CE: *n* = 5). (**G** and **H**) Representative images of sorted synaptosomes. (G) eGRASP+: YFP (eGRASP, green) and Homer 1 (postsynaptic, magenta); (H) eGRASP−: vGlut1 (presynaptic, green). Scale bars, 1 μm. (**I**) Sunburst plots (CC) of overlap between identified CFC or CE proteins and SynGO annotated proteins. Labels are the same for CE and CFC. Terms with 0% coverage: (i) synaptic vesicle lumen (SynGO, one protein), (ii) presynaptic actin cytoskeleton (SynGO, two proteins), and (iii) postsynaptic specialization membrane of symmetric synapse (SynGO, one protein). (**J**) Presynaptic and postsynaptic protein abundance. Box plot: median, 25th, and 75th percentiles. Each data point equals one protein (presynapse, green; postsynapse, pink). (**K**) Principal components analysis of eGRASP+ (green) and eGRASP− (gray) samples. CFC (circles, *n* = 8) and CE (squares, *n* = 9). Each data point equals one mouse. (**L** and **M**) Proteins differentially expressed in eGRASP+ synaptosomes after (L) CFC and (M) CE. Volcano threshold: *q* < 0.01. Labels: 20 most significantly (*q* < 0.01) regulated proteins.

We combined eGRASP, synaptosome isolation, and sorting with sensitive ion-mobility MS to examine the proteome of samples enriched for sparsely distributed and fluorescently labeled CA3-CA1 contextual memory-storing engram cell synapses. Animals underwent aversive CFC and CE to generate aversive (CFC) and neutral (CE) hippocampal-dependent contextual memories, respectively, and were euthanized 72 hours later for synaptic proteome analyses (Fig. 2A).

Using electron microscopy, immunoblotting, and immunohistochemical colocalization, we confirmed ultrastructural integrity, synaptic-protein enrichment, and presynaptic with postsynaptic apposition of synaptosome preparations (fig. S2, A to C). We used a size- and fluorescence-based sorting strategy instead of FM4-64 fluorescence-triggered sorting (*25*) to enrich and harvest biochemically isolated eGRASP-expressing synaptosomes (Fig. 2B and fig. S3). This was necessary because the amphiphilic styryl dye FM4-64 interfered spectrally with RFP detection in myristoylated RFP– and YFP-expressing eGRASP+ synaptosomes. Reanalysis of the sorted eGRASP+ (RFP+YFP+) fraction demonstrated an average sevenfold enrichment of eGRASP+ events (CFC: unsorted = 7.09 ± 0.82% to sorted = 46.35 ± 1.86%; CE: unsorted = 6.22 ± 0.87% to sorted = 45.56 ± 1.36%), and qualitative back-gating analysis of sorted, reanalyzed eGRASP+ and eGRASP− populations confirmed this enrichment (Fig. 2, C and D, and fig. S3, D and E). Furthermore, analysis of a representative subset of these samples revealed no differences in forward scatter (FSC) and side scatter (SSC) parameters between reanalyzed eGRASP+ and eGRASP− events, indicating similar particle size distributions across sorted populations (fig. S3, F to H). To characterize the extent to which sorted samples contained synaptosomes with attached pre- and postsynapses, we immunolabeled sorted eGRASP+ (RFP+YFP+) and eGRASP− (RFP−YFP−) fractions with markers for these synaptic subcompartments. Approximately 60% of imaged particles were considered as single synaptosomes after applying exclusion criteria (*26*) to discard nonquantifiable events, such as aggregates (fig. S4A; see Materials and Methods for all exclusion criteria). These single synaptosomes were then assessed for pre- and postsynaptic colocalization using protein marker intensity to define quadrant gates that split positive and negative expression for each label (Fig. 2, E and F, and fig. S4B) (*26*). Quadrant 3 (Q3) exhibited the highest Spearman correlation coefficient between pre- and postsynaptic protein intensities [eGRASP+ synaptosomes: YFP+SYP+: 0.46 (fig. S4B); YFP+Homer1+: 0.10 (Fig. 2E); eGRASP− synaptosomes: vGlut1+Homer1+: 0.60 (Fig. 2F)], consistent with Q3 representing synaptosomes positive for both pre- and postsynaptic markers. A vast majority of double-positive synaptosomes fall within this quadrant, confirming that they retain both pre- and postsynaptic components [eGRASP+ synaptosomes: YFP+SYP+: 90.2% [95% confidence interval (CI) = 88.89 to 91.46%] (fig. S4B); YFP+Homer1+: 78.1% [95% CI = 75.97 to 80.10%] (Fig. 2E); eGRASP− synaptosomes: vGlut1+Homer1+: 85% [95% CI = 83.31 to 86.56%] (Fig. 2F)]. High-resolution rescan confocal imaging of these same synaptosomes confirmed the apposition of pre- and postsynaptic terminals (fig. S4C and Fig. 2, G and H). Together, these results demonstrate that eGRASP+ events are enriched for labeled synapses, while eGRASP− events enrich for heterogeneous unlabeled synapses from the dorsal hippocampus, with both fractions containing a similar proportion of single synaptosomes.

From ~5500 uniquely identified proteins across all groups (median coefficient of variation % = 18.75 ± 0.29%) (fig. S5, A and B), 4595 (CFC) and 4550 (CE) proteins were quantified in at least half of the samples within each experimental condition (data S1 to S3) and used for downstream analyses. Peptide depth and MS signal intensities were highly comparable between eGRASP+ and eGRASP− fractions across both CFC and CE experimental conditions (fig. S5, C to E). Median protein abundance values and variance were consistent across groups (CFC eGRASP+: 10.90 ± 1.48; CFC eGRASP−: 10.96 ± 1.49; CE eGRASP+: 10.94 ± 1.47; CE eGRASP−: 11.02 ± 1.49; fig. S5F). From those quantified, 1182 (CFC) and 1178 (CE) proteins were annotated in the curated synaptic gene ontology database SynGO (*27*) and showed high overlap with 91 of 94 defined synaptic subcompartments (Fig. 2I and data S4). A comparison of SynGO-annotated (i.e., evidenced synaptic) and SynGO-nonannotated (i.e., putatively “nonsynaptic”) protein abundancies demonstrated that both eGRASP+ and eGRASP− populations were enriched for synaptic compared to nonsynaptic proteins [median interquartile range (IQR) log_2_ intensities for SynGO versus non-SynGO proteins, CFC+: 11.19 (10.50 to 12.08) versus 10.78 (10.09 to 11.72); CFC−: 11.18 (10.52 to 12.04) versus 10.86 (10.17 to 11.82); CE+: 11.21 (10.51 to 12.08) versus 10.81 (10.13 to 11.76); CE−: 11.20 (10.51 to 12.08) versus 10.81 (10.13 to 11.76)] (fig. S5G). In addition, there was no difference in overall SynGO protein abundancy between labeled and unlabeled conditions, demonstrating that the bulk synaptic proteome remains quantitatively stable across eGRASP+ and eGRASP− populations (fig. S5G). An equal distribution between pre- and postsynaptic protein abundancies was observed in eGRASP+ and eGRASP− samples across both groups, as evidenced by a mean abundance ratio of 1.03 (CFC, +/presynapse: 11.7 ± 0.06; +/postsynapse: 11.2 ± 0.04; −/presynapse: 11.6 ± 0.06; −/postsynapse: 11.2 ± 0.04; CE, +/presynapse: 11.6 ± 0.06; +/postsynapse: 11.3 ± 0.04; −/presynapse: 11.7 ± 0.05; −/postsynapse: 11.3 ± 0.04) (Fig. 2J). Together, these analyses indicate synaptic protein enrichment and proteome comparability across sorted populations and experimental groups.

Principal components analysis explained 33.2% of the total variance (PC1: 18.9%; PC2: 14.3%) primarily segregating samples on the basis of synapse labeling and, therefore, activation (eGRASP+ and eGRASP−), with no discernible batch effect of other experimental parameters such as date of (i) conditioning or (ii) synapse sorting (Fig. 2K and fig. S5, H to K). To uncover the drivers of these proteome differences, we performed differential expression analysis (DEA) using a group-wise paired-sample design (eGRASP− versus eGRASP+; data S3). From proteins quantified with at least two peptides, 61 proteins were enriched, and 103 proteins were depleted in eGRASP+ synapses after CFC when compared to the eGRASP− cohort [false discovery rate (FDR) *q* < 0.01; data S5]. In the CE group, 52 proteins were enriched, and 142 proteins were significantly depleted (FDR *q* < 0.01; data S5). The use of a *CaMKII*α-driven pre-eGRASP construct was supported by the significant association of glutamatergic neuronal cell types for enriched proteins [fig. S6, A and B; single-cell gene expression data for the hippocampus were obtained from the Allen Brain Atlas (*28*), and bootstrapping was performed with 10,000 repetitions]. Given that the enrichment of Nlgn1 in both groups was driven by peptide sequences shared with the eGRASP construct (*10*), we removed all eGRASP-derived neurexin and neuroligin peptides before rerunning quantification and DEA (fig. S6C and data S3). As expected, only Nlgn1 lost statistical significance, while all other proteins retained their significance and effect sizes under these filtered conditions, resulting in 60 enriched and 103 depleted proteins after CFC and 51 enriched and 142 depleted proteins after CE (Fig. 2, L and M; fig. S6, D to F; and data S5).

### Validation of protein enrichment using immunolabeling flow cytometry

**Fig. 3.**
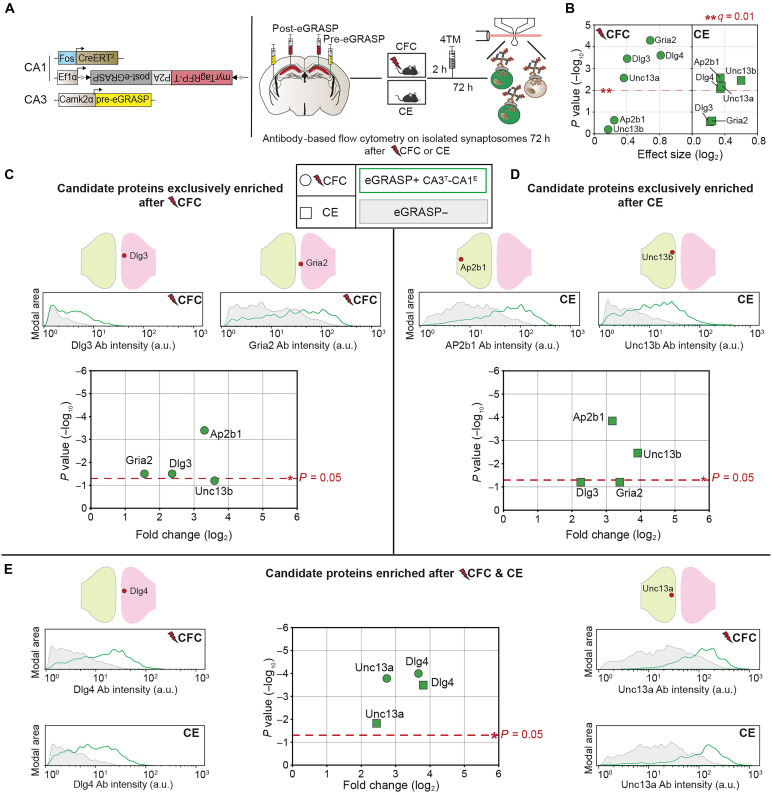
Validation of protein enrichment using immunolabelling flow cytometry. (**A**) Experimental setup. Independent cohorts of mice injected with eGRASP constructs to label total CA3 input onto CA1 engram cells (CA3^T^-CA1^E^ synapses). Isolation and flow analysis of immunolabelled eGRASP+ and eGRASP− synaptosomes 72 hours after CFC or CE. (**B**) Candidate proteins for immunolabeling flow cytometry. Log_2_ effect size (*x* axis) and −log_10_ *q* value (*y* axis) derived from MS-DEA analysis. Dotted line: significance drawn at **q = 0.01. (**C**) Left: eGRASP+ synaptosomes exhibit significant enrichment of Gria2 (one-tailed Wilcoxon signed-rank test, *P* = 0.0312) and Dlg3 (one-tailed Wilcoxon signed-rank test, *P* = 0.0312) exclusively after CFC. (**D**) Left: eGRASP+ synaptosomes exhibit significant enrichment of Unc13b exclusively after CE (one-tailed paired *t* test, *P* = 0.0035). eGRASP+ synapses exhibit significant enrichment of Ap2B1 after both CFC (two-tailed paired *t* test, *P* = 0.0004) and CE (one-tailed paired *t* test, *P* = 0.000145). (**E**) Center: eGRASP+ synaptosomes exhibit significant enrichment of Dlg4 (one-tailed paired *t* test, CFC: *P* = 0.0001; CE: *P* = 0.0003) and Unc13a (one-tailed paired *t* test, CFC: 0.0002; CE: 0.0146; *n* = 5) after CFC and CE. [(C) to (E)] Comparisons made to eGRASP− counterparts. Dotted line: significance drawn at *P = 0.05. CFC: *n* = 5; CE: *n* = 4 or 5. Corresponding representative antibody fluorescence intensity distribution profile shown for eGRASP+ (green) and eGRASP− (gray) samples.

To validate MS results, we performed immunolabeling flow cytometry to examine the enrichment of selected proteins in labeled eGRASP+ samples relative to unlabeled eGRASP− samples harvested from an independent cohort of CFC and CE animals (Fig. 3A and fig. S7). Six candidate proteins were selected for validation: four showing condition-specific enrichment (Gria2 and Dlg3 after CFC; Unc13b and Ap2b1 after CE) and two proteins enriched after both CFC and CE (Dlg4 and Unc13a) (Fig. 3B).

Consistent with MS findings, flow cytometry revealed a higher intensity distribution profile of immunolabeled proteins in eGRASP+ CA3^T^-CA^E^ synaptosomes compared to their untagged counterparts (Fig. 3, C to E). Specifically, postsynaptic Gria2 [fold change (FC): 1.6 ± 0.27] and Dlg3 [FC: 2.4 ± 0.11] were significantly enriched only after CFC (Fig. 3C), while presynaptic Unc13b (3.9 ± 0.26) showed significant enrichment exclusively after CE (Fig. 3D). Both Dlg4 (FC—CFC: 3.7 ± 0.46; CE: 3.8 ± 0.13) and Unc13a (FC—CFC: 2.7 ± 0.11; CE: 2.4 ± 0.29) were significantly enriched in both conditions (Fig. 3E). Ap2b1 was significantly enriched after both CFC and CE diverging from the CE-exclusive regulation observed in our MS data (FC—CFC: 3.3 ± 0.1; CE: 3.2 ± 0.1) (Fig. 3D). In summary, these findings largely corroborate experience-dependent and shared protein enrichment signatures following CFC and CE in an independent group of animals.

### Proteins differentially expressed in sorted CA1 engram cell synapses display a unique molecular signature

**Fig. 4.**
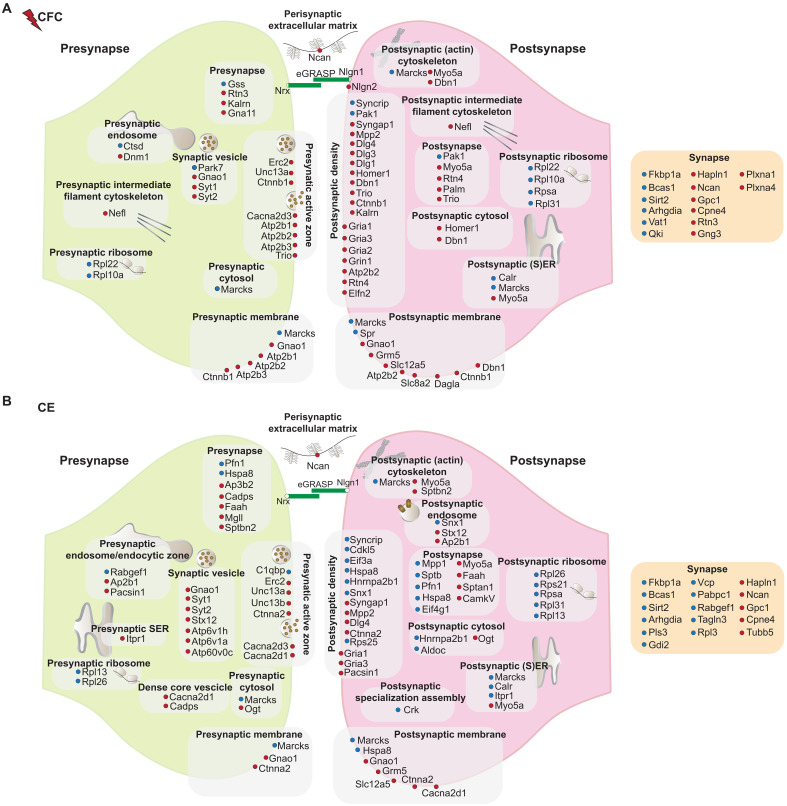
Differentially expressed proteome of CA1 engram cell synapses relative to an unlabeled comparator. (**A** and **B**) SynGO CC subsynaptic annotation of differentially expressed proteins 72 hours after (A) CFC and (B) CE. Proteins significantly enriched and depleted in eGRASP+ samples are depicted in red and blue, respectively. A single protein may be annotated to more than one location in the synapse.

Proteins enriched or depleted in eGRASP+ synapses covered a range of protein classes and higher-order physiological functions related to synapse function, metabolism, and protein turnover (figs. S8 and S9 and data S6 and S7). Subsequent data interpretation of synaptic function was focused on proteins annotated to the curated synapse database SynGO-v1.2 (*27*). From significantly enriched/depleted proteins (Fig. 2, L and M), 61 CFC proteins and 69 CE proteins had subsynaptic localizations in SynGO (Fig. 4, A and B, and data S8) and were used for downstream analyses.

**Fig. 5.**
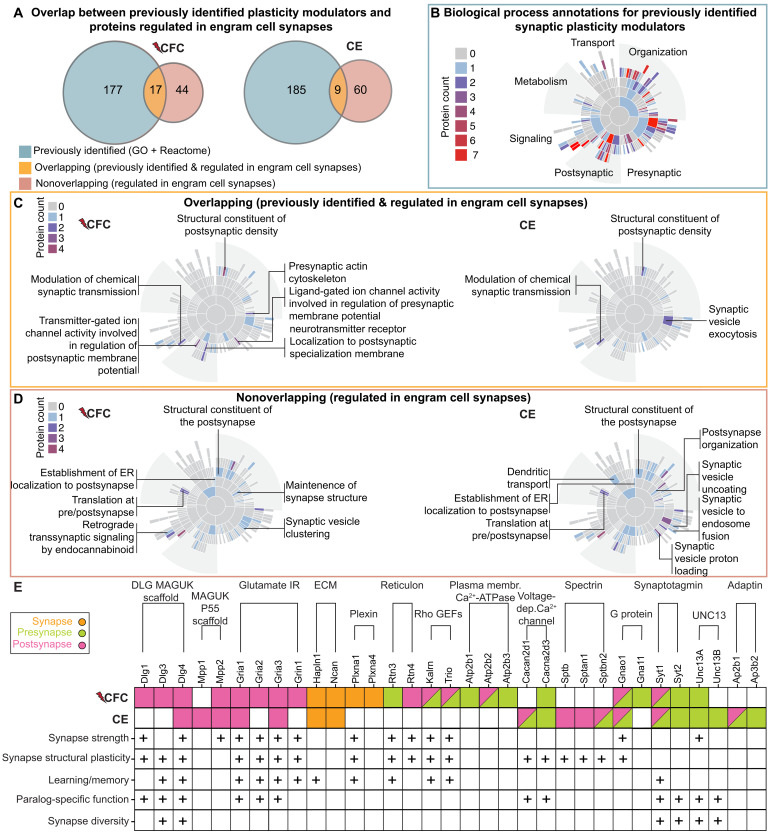
Proteins differentially expressed in sorted CA1 engram cell synapses display a unique molecular signature. (**A**) Overlap between 194 SynGO proteins, belonging to GO-0048167: regulation of synaptic plasticity and Reactome: Long Term Potentiation, and 61 CFC-regulated SynGO proteins (left) or 69 CE-regulated SynGO-proteins (right). (**B**) Previously identified synaptic plasticity modulators (167 of 194 proteins with a BP annotation) are involved in a variety of BPs at the synapse. (**C**) Previously identified plasticity proteins regulated in engram cell synapses [overlapping proteins in (A); CFC: 17; CE: 9] are involved in a subset of higher-order (second level in the sunburst) BPs depicted in (B). Terms with a protein count of more than 1 are labeled. (**D**) Regulated engram cell synapse proteins [nonoverlapping proteins in (A); CFC: 26 of 44; CE: 36 of 60 proteins with a BP annotation] are mostly involved in the same BPs as previously identified modulators [as depicted in (B)], albeit being different in identity. Some proteins also specialize in processes not implicated by previously identified plasticity modulators. These terms are labeled for CFC (left) and CE (right). (**E**) Protein paralogs regulated after CFC or CE categorized by their functional groups and SynGO-CC annotation. Paralogs previously implicated in synapse strength, structural plasticity, learning/memory, paralog-specific function, and synapse diversity are indicated with a “+,” with source example references listed in data S10. SynGO-CC annotation: presynapse (green), postsynapse (pink), synapse (orange), and not regulated (white).

CA1 engram cell synapses receiving input from CA3 excitatory neurons exhibit structural and functional plasticity evidenced by larger spine volume after both CFC and CE (Fig. 1I and fig. S10A: spine volume—CFC: 0.4 ± 0.01; CE: 0.41 ± 0.02) and enhanced AMPAR-mediated synaptic transmission after CFC (*10*). Therefore, we investigated whether SynGO proteins differentially expressed in these synapses 72 hours after learning may have biological function related to typical synaptic plasticity processes such as long-term potentiation (LTP). Specifically, we assessed the overlap between proteins regulated in labeled CA1 engram cell synapses (CFC: 61 proteins; CE: 69 proteins) and previously identified plasticity modulators (194 SynGO-annotated proteins belonging to GO-0048167: Regulation of synaptic plasticity and Reactome: Long Term Potentiation; data S9). Notably, only a small subset of regulated proteins overlapped with this reference plasticity set (CFC: 17 proteins; CE: 9 proteins; Fig. 5A and data S9). These overlapping proteins mapped to a specific subset of the higher-order biological processes (BPs) as known plasticity modulators, related to bidirectional synaptic function, e.g., pre- and postsynaptic coactivation, postsynaptic structure, and modulation of chemical transmission (Fig. 5, B and C, and data S9). The remaining nonoverlapping proteins from each experimental group were involved in (i) similar BPs as known synaptic plasticity modulators (Fig. 5D and data S9) and (ii) different higher-order BPs related to synapse structure, synaptic vesicle cycle, and translation and transport at the synapse (Fig. 5D and data S9). Together, while labeled eGRASP+ CA1 engram cell synapses exhibit molecular remodeling after learning, most of differentially expressed proteins do not fall within the canonical set of LTP-associated factors and map to similar but not identical BPs implicated thus far in plasticity processes.

Next, we determined whether synaptic protein paralogs that likely evolved after consecutive genome duplications to enable specialization of protein function exhibit differential expression in response to contextual learning stimuli (*29*, *30*). We detected paralogs, including postsynaptic MAGUK scaffolds, glutamate receptors, presynaptic plasma membrane adenosine triphosphatases, heterotrimeric guanine nucleotide–binding proteins, Unc proteins, and adaptins, as differentially regulated in labeled CFC and CE engram cell synapses (Fig. 5E and data S10). Of these, several have been previously implicated in modulation of synapse strength, structural plasticity, learning and memory, synapse diversity, and/or exhibiting paralog-specific function (Fig. 5E and data S10). Moreover, relative to unlabeled controls, we observed experience-dependent differential expression of paralogs, such as postsynaptic Dlg1 and Dlg3 enriched after CFC and Dlg4 after both CFC and CE and presynaptic Unc13b enriched after CE and Unc13a after both CFC and CE (Fig. 5E and data S10). These findings support the idea that contextual learning recruits a specialized set of synaptic paralogs, potentially enabling fine-tuned modulation of synapse function according to distinct memory demands.

Aberrant hippocampal synapse function is a critical feature of Alzheimer’s disease (AD) that correlates with cognitive decline (*31*). We hypothesized that CA1 engram cell synapses contain key molecular substrates that are dysregulated with progressive worsening of synaptic function and memory in AD. Comparison of our CFC- and CE-regulated proteomes to SynGO-annotated proteins dysregulated in human postmortem CA1/subiculum tissue of AD across all Braak stages (0 to VI) (*32*) showed an overlap of 15 proteins (fig. S10B and data S11). These include, among others, presynaptic targets of the synaptic vesicle and trafficking proteins (Syt1, Dnm1, and Ap2b1) and postsynaptic glutamate receptors (Gria1/2/3 and Grin1) (fig. S10B and data S11). Most of these proteins showed opposite patterns of differential expression in the two datasets, i.e., down-regulated in AD tissue and enriched in CA1 engram cell synapses, suggesting that their dysregulation may be linked to cognitive deficits in AD.

Given that CA1 engram cell synapses specifically adapt their synaptic proteome relative to their unlabeled counterparts, we reasoned that these adaptations encompass key molecular determinants of cognitive phenotypes and their related neuropsychiatric disorders and dementias. To increase statistical power and minimize the likelihood of chance overlaps, we expanded our analysis beyond SynGO proteins to include all enriched proteins following CFC and CE. We first tested whether genome-wide association study (GWAS) traits related to intelligence, memory, well-powered synaptopathies, and dementia-related phenotypes were enriched in this DEA dataset. While “educational attainment” (associated proteins: Dlg1, Dagla, Pkp4, Syt1, Kalrn, Gfap, Dbn1, Ryr2, and Cpne4) and “post-traumatic stress disorder” (PTSD; associated proteins: Gria1 and Slc12a5) were enriched for CFC, “educational attainment” (associated proteins: Camkv, Ctnna2, Sptbn2, Ap2b1, Syt1, Ryr2, Fbxo41, and Cpne4), “PTSD” (associated proteins: Camkv, Gria1, and Slc12a5), and “schizophrenia” (associated proteins: Ncan, Ctnna2, Syngap1, and Ap3b2) (fig. S10, C to E) were enriched after CE. No enrichment was found for “AD,” “attention deficit/hyperactivity disorder,” “intelligence quotient,” “short-term memory,” or a well-powered control GWAS of “serum urate levels” (data S12). No GWAS trait enrichment was found in a control dataset containing the least differentially regulated proteins common to both experimental groups (fig. S10C and data S12). To assess rare variants, we queried the Online Mendelian Inheritance in Man (OMIM) database for genes causal to neurological Mendelian disorders with cognitive/intellectual/memory phenotypes or symptoms. The proportion of genes linked to these phenotypes was significantly higher for the CFC (23 of 60 = 38.33%; *P* = 2.37 × 10^−06^)– and CE (20 of 51 = 39.22%; *P* = 2.34 × 10^−05^)–enriched proteomes when compared to the nonsignificantly regulated control dataset (28 of 100 = 28%) (fig. S10, D and E; list of variants and disorders in data S12).

Overall, eGRASP+ CA1 memory engram cell synapses show enhanced structural and functional connectivity (*33*) paralleled by a distinct molecular signature relative to eGRASP− samples. This includes paralogous proteins with experience-dependent expression patterns and a protein landscape associated with cognitive traits and disorders.

### Salient aversive context memory is reflected as dominant postsynaptic protein expression patterns

CA1 encodes contextual representations after both CFC and CE. However, associating negative valence to context results in a more salient memory when compared to neutral CE (*34*). Thus, we next investigated whether this enhanced salience emerges at the level of engram cell synapses.

The limited separation between eGRASP+, aversive CFC and neutral CE samples in the principal components analysis (Fig. 2K) suggests an overlap in the synaptic proteome of CA1 engram cells involved in encoding contextual memories of varying salience. On the other hand, experience-dependent regulatory patterns became apparent when proteins were grouped by class and function (figs. S8 and S9 and Fig. 5, C to E). Together, this is suggestive of CFC and CE memory processing involving both shared and specialized molecular mechanisms. Direct DEA between eGRASP+ CFC and eGRASP+ CE conditions revealed no significant differentially expressed proteins. This outcome likely reflects a combination of factors, including dilution effects caused by the nonenriched complement present in eGRASP+ synaptosomes, as well as inherent limitations of traditional DEA methods such as MSqRob. These methods evaluate each protein independently and may fail to detect modest but coordinated changes in protein abundance that reflect meaningful biological variation (*35*). To overcome these analytical constraints and enable robust comparison of experience-dependent protein signatures between CFC and CE conditions, we extended the MSqRob DEA results from within-group comparisons (eGRASP− versus eGRASP+) for both CFC and CE groups by implementing robust (Huber) linear regression (*36*) coupled with comparative dominance analysis. This integrated approach stabilizes model performance in noisy datasets by down-weighting outliers and ranks proteins by their contribution to group-level variance, allowing us to prioritize functionally relevant proteins that may be underpowered in traditional DEA.

**Fig. 6.**
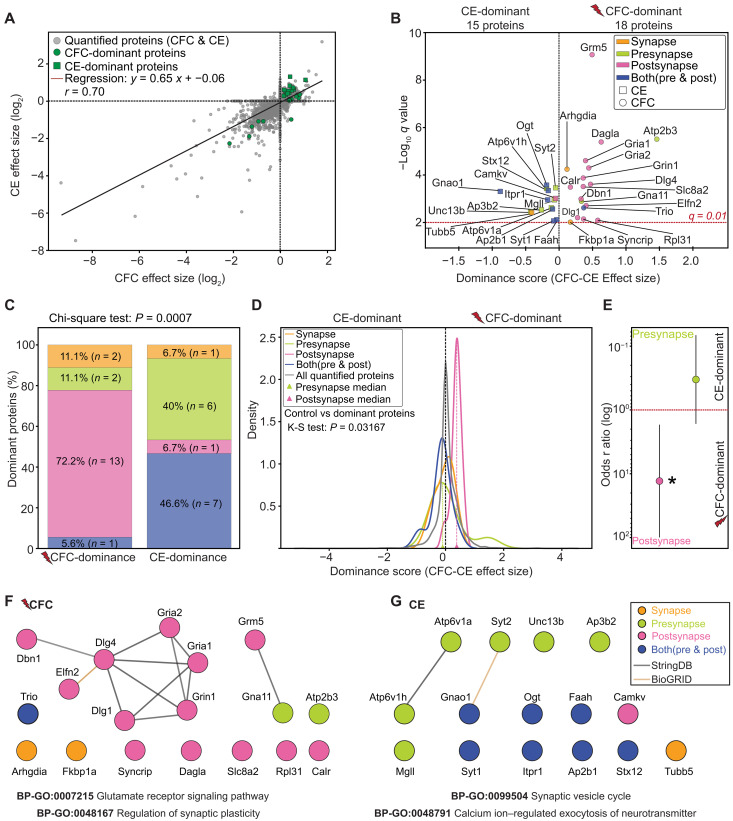
Salient aversive context memory is reflected as dominant postsynaptic protein expression patterns. (**A**) Scatterplot of effect sizes of quantified CFC (*x* axis) and CE (*y* axis) proteins. CFC-dominant SynGO proteins (green circles) and CE-dominant SynGO proteins (green squares) highlighted. The regression line (red) indicates a positive correlation (*y* = 0.65*x* + 0.06, Pearson *r* = 0.70). (**B**) Volcano plot of dominance scores (CFC-CE effect size, *x* axis) and −log_10_ *q* values (*y* axis) for SynGO-dominant proteins. CFC-dominant (circles) and CE-dominant (squares) proteins are categorized by SynGO-CC synaptic location. The dashed red line indicates the *q*-value threshold of 0.01. (**C**) Stacked bar chart displaying distribution of SynGO-dominant proteins across synaptic location categories. Chi-square test confirms significant association between dominance and synaptic location (*P* = 0.0007; Chi2 Stat: 16.9867; df = 3). (**D**) Kernel density plots showing distribution of dominance scores (CFC-CE effect size) for SynGO-dominant proteins categorized by synaptic location categories. Median dominance scores for pre- and postsynaptic proteins are indicated (triangles). The K-S test confirms a significant deviation of SynGO-dominant distributions from the control distribution (*P* = 0.0317; K-S Stat = 0.2460). (**E**) Forest plot showing ORs for post- and presynaptic proteins. Postsynaptic proteins exhibit significantly higher odds of being CFC-dominant (*P = 0.013). Error bars represent 95% CIs, with OR = 1 (dotted line) indicating no association [presynapse CI (0.05, 2.7); postsynapse CI (1.7, 100)]. (**F** and **G**) Protein interaction network and corresponding STRINGDB GO-BPs for (F) CFC-dominant and (G) CE-dominant proteins. Edges represent a STRINGDB score of >0.7 (high confidence) and BioGRID-curated PPI interactors. [(B) to (G)] SynGO-CC annotations for synaptic location: presynapse (green), postsynapse (pink), both (blue), and synapse (orange).

Huber regression revealed a strong positive correlation between effect sizes of both experimental groups (Pearson *r* = 0.70; Fig. 6A), and comparative dominance analysis leveraging effect size differences, prediction residuals, SynGO annotations, and significance thresholds (see Materials and Methods) identified CFC- and CE-dominant proteins (Fig. 6A and data S13). The alignment of these dominant proteins along the regression line indicates the functional specialization of synaptic proteins in response to CFC or CE valence.

Next, we categorized the 33 dominant proteins (CFC: 18; CE: 15) by their SynGO-annotated subsynaptic location (“Presynapse,” “Postsynapse,” “Both,” or “Synapse”) and dominance scores (Fig. 6B and data S13). Several CFC-dominant proteins, including Grm5, the most significantly dominant protein; Dbn1; Gria1; Gria2; Grin1; Dagla; and Dlg4, were strongly associated with the postsynaptic compartment. Conversely, CE-dominant proteins, such as Syt2, Unc13b, Ap3b2, Atp6v1h, and Atp6v1a, are primarily localized to the presynapse. Quantitative analysis revealed that most CFC-dominant proteins (72.2%) were postsynaptic, whereas CE-dominant proteins were more evenly distributed to both compartments (46.6%) or to the presynapse (40%) (Fig. 6C and data S13). A chi-square test confirmed a significant association between dominance group (CFC/CE) and synaptic locations (Fig. 6D). Extending our findings on dominance patterns, kernel density estimation (KDE) analysis revealed that CFC-dominant proteins skewed positively toward postsynaptic dominance, while CE-dominant proteins skewed negatively toward presynaptic dominance (Fig. 6D). A significant Kolmogorov-Smirnov (K-S) test result confirmed that dominance score distributions of dominant proteins was nonrandom compared to the control distribution of all quantified proteins (Fig. 6D). We then tested a logistic regression model to determine whether synaptic location predicts dominance between CFC and CE (logistic regression *P* = 0.0008; pseudo *R*^2^ = 0.316). This analysis confirmed that postsynaptic proteins were ~13 times more likely to exhibit CFC dominance than CE dominance {log [odds ratio (OR)] = 2.56; Fig. 6E}. In contrast, presynaptic proteins leaned toward CE dominance, albeit not significantly [log (OR) = −1.10; Fig. 6F]. Protein-protein interaction (PPI) networks further revealed that CFC-dominant proteins contain a functional module of postsynaptic proteins involved in receptor signaling and synaptic plasticity (GO: 0007215; GO: 0048167; Fig. 6F). Conversely, smaller presynaptic functional modules emerged for CE-dominant proteins, including those involved in synaptic vesicle cycling and neurotransmitter release (GO: 0099504; GO: 0048791; Fig. 6G).

Last, we used a phenome-wide association study (PheWAS) to investigate whether proteins dominant after CFC or CE are genetically associated with cognitive and neuropsychiatric phenotypes derived using human gene-based GWAS data. For CFC, significant associations were identified for Dagla, Gria1, and Syncrip with educational attainment and qualifications; Dlg4 with reaction time; and Dbn1 with cognitive performance, intelligence, reasoning, and education (data S14). In the CE group, Ap2b1 was linked to education; Gnao1 to reaction time; Ap3b2 to cognitive performance, schizophrenia, bipolar disorder, and intelligence; Camkv to cognitive performance, intelligence, and education; and Tubb5 to reasoning and schizophrenia (data S14).

Our findings indicate that salient aversive contextual memories exhibit a significant dominance of postsynaptic protein expression relative to eGRASP− controls, while less salient neutral contextual representations are characterized by a tendency toward presynaptic protein changes. In addition, PheWAS analysis associates these dominant proteins with several human cognitive traits and neuropsychiatric conditions.

### Molecular and structural analysis of CA1 engram cell synaptic subpopulations

CA1 engram dendrites are preferentially connected to CA3 engram cells before CFC (*11*). Our proteomic analyses thus far focused on samples enriched for eGRASP-labeled CA3^T^-CA1^E^ synapses, encompassing dorsal CA3 synaptic input onto dorsal CA1 engram cells. This total-to-engram population comprises the following:

1) CA3^E^-CA1^E^ synapses between CA3 engram cells and CA1 engram cells coactivated by learning. These include (i) preexisting engram-to-engram synapses between preconnected CA3 and CA1 neurons that are allocated to the engram ensemble during learning and (ii) newly formed engram-to-engram synapses that form de novo between CA3 engram cells and CA1 engram cells after learning.

2) CA3^NE^-CA1^E^ synapses between CA3 nonengram cells and CA1 engram cells.

**Fig. 7.**
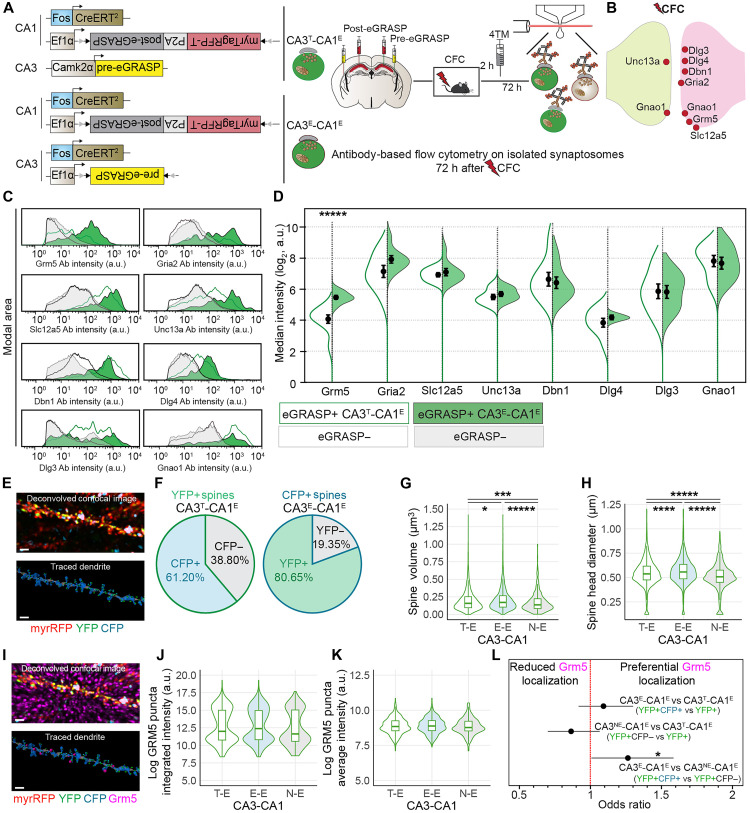
Molecular and structural analysis of CA1 engram cell synaptic subpopulations. (**A**) Experimental setup. Independent cohorts of mice with post-eGRASP and viral-TRAP in CA1 and either (i) yellow pre-eGRASP in CA3 (CA3^T^-CA1^E^ synapses) or (ii) yellow pre-eGRASP with viral-TRAP in CA3 (CA3^E^-CA1^E^ synapses). Isolated synaptosomes analyzed by immunolabeling flow cytometry 72 hours after CFC. (**B**) Candidate proteins for immunolabeling flow cytometry. (**C**) Fluorescence intensity distributions from eGRASP+ (green) and eGRASP− (gray) synaptosomes for CA3^E^-CA1^E^ and CA3^T^-CA1^E^ conditions. Ab, antibody. (**D**) Violin plots of median fluorescence intensity (log_2_) with means ± SEM, comparing CA3^T^-CA1^E^ and CA3^E^-CA1^E^ eGRASP+ synaptosomes. Significance observed for Grm5 (*n* = 8 per group, unpaired two-tailed *t* test, *****P = 0.00004). [(B) and (C)] CA3^T^-CA1^E^: green-open distributions; CA3^E^-CA1^E^: green-filled distributions. (**E** to **L**) Mice expressing dual-eGRASP to label CA3^T^-CA1^E^ (yellow) and CA3^E^-CA1^E^ synapses (cyan). (E) Representative deconvolved, traced dendrite with YFP and CFP spots. Scale bar, 2 μm. (F) Pie charts of CFP/YFP colocalization in traced spines per dendrite (*n*_dendrites_ = 47; *n*_mice_ = 5). [(G) and (H)] Violin/box plots demonstrating the largest volume (G) and head diameter (H) of CA3^E^-CA1^E^ spines (GLMM with clustering by dendrite and animal; Bonferroni-corrected post hoc, *n*_spinesT-E_ = 2245, *n*_spinesE-E_ = 1388, and *n*_spinesNE-E_ = 857; spine volume: *P_adjT-Evs.E-E_ = 0.0327 , ***P_adjT-Evs.N-E_ = 0.0004 , and *****P_adjE-Evs.N-E_ < 0.0001; spine head diameter: ****P_adjT-Evs.E-E_  = 0.0002, *****P_adjT-Evs.N-E_ < 0.0001, and *****P_adjE-Evs.N-E_ < 0.0001). (I) Representative deconvolved, traced dendrite with YFP, CFP, and Grm5 spots. Scale bar, 2 μm. [(J) and (K)] Violin/box plots of Grm5 puncta log-integrated intensity (J) and log average intensity (K) across spine populations (*n*_spinesT-E_ = 846, *n*_spinesE-E_ = 551, and *n*_spinesNE-E_ = 295). (L) Forest plots showing Grm5 colocalization ORs (95% CI, OR = 1 dotted line). Grm5 localization higher in CA3^E^-CA1^E^ versus CA3^NE^-CA1^E^ spines (hierarchical mixed-effects logistic regression, *P = 0.039; *n*_spinesYFP+_ = 2245, *n*_spinesYFP+CFP+_ = 1388, and *n*_spinesYFP+CFP−_ = 857).

Given this heterogeneity of synapses within the total-to-engram population, we next asked to what extent protein expression signatures of CA3^T^-CA1^E^ synapses reflect molecular changes in the smaller and functionally more specific CA3^E^-CA1^E^ synaptic pool. To examine this, we performed immunolabeling flow cytometry in independent cohorts of animals to compare protein expression in CA3^T^-CA1^E^ and CA3^E^-CA1^E^ synapses after CFC. We focused on eight enriched and/or dominant proteins, including the most significantly dominant CFC protein, Grm5; paralogs such as Dlg3 and Dlg4; and the PTSD-associated protein Slc12a5 (Fig. 7, A and B).

Both CA3^T^-CA1^E^ and CA3^E^-CA1^E^ eGRASP+ populations demonstrated elevated protein expression profiles relative to eGRASP− samples, once again validating total-to-engram MS findings and extending them to the smaller population of engram-to-engram synapses (Fig. 7C). Direct comparison of eGRASP+ CA3^T^-CA1^E^ and CA3^E^-CA1^E^ populations revealed Grm5 as significantly more abundant in CA3^E^-CA1^E^ synapses (5.47 ± 0.12) compared to the CA3^T^-CA1^E^ population (4.07 ± 0.27) (Fig. 7D). In contrast, expression levels of the remaining proteins including Gria2 (CA3^E^-CA1^E^: 7.92 ± 0.24; CA3^T^-CA1^E^: 7.14 ± 0.39), Slc12a5 (CA3^E^-CA1^E^: 7.10 ± 0.24; CA3^T^-CA1^E^: 6.92 ± 0.14), Unc13a (CA3^E^-CA1^E^: 5.69 ± 0.15; CA3^T^-CA1^E^: 5.50 ± 0.17), Dbn1 (CA3^E^-CA1^E^: 6.41 ± 0.37; CA3^T^-CA1^E^: 6.64 ± 0.44), Dlg4 (CA3^E^-CA1^E^: 4.10 ± 0.07; CA3^T^-CA1^E^: 3.84 ± 0.28), Dlg3 (CA3^E^-CA1^E^: 5.82 ± 0.41; CA3^T^-CA1^E^: 5.86 ± 0.47), and Gnao1 (CA3^E^-CA1^E^: 7.67 ± 0.38; CA3^T^-CA1^E^: 7.81 ± 0.36) did not differ between these two synapse subpopulations (Fig. 7D).

The similar protein expression profiles of seven of eight tested proteins in CA3^T^-CA1^E^ synapses and CA3^E^-CA1^E^ synapses suggest that total-to-engram synapses largely reflect the protein expression signatures of engram-to-engram synapses. This is possibly due to engram-to-engram synapses comprising a substantial proportion of total-to-engram synapses. To determine the overlap between these two synaptic populations, we labeled them within the same animal using dual-eGRASP, tagging CA3^T^-CA1^E^ synapses with eGRASP-YFP and CA3^E^-CA1^E^ synapses with eGRASP-cyan fluorescent protein (CFP). In line with previous structural data (*10*, *11*), we found that a considerable percentage of CA3^T^-CA1^E^ (YFP+) synapses also expressed CFP (61.20 ± 1.54%), while a large majority of CA3^E^-CA1^E^ (CFP+) synapses also expressed YFP (80.65 ± 1.26%) (Fig. 7, E and F). These data demonstrate that CA3^E^-CA1^E^ synapses make up a larger fraction of CA3^T^-CA1^E^ synapses than their CA3^NE^-CA1^E^ counterparts. In addition, these CA3^E^-CA1^E^ (YFP+CFP+) synapses had a larger volume and spine head diameter compared to putative CA3^NE^-CA1^E^ (YFP+CFP−) synapses on the same dendrites (median spine volume—CA3^T^-CA1^E^: 0.16; IQR: 0.16; CA3^E^-CA1^E^: 0.17; IQR: 0.16; CA3^NE^-CA1^E^: 0.14; IQR: 0.14; spine head diameter—CA3^T^-CA1^E^: 054; IQR: 0.14; CA3^E^-CA1^E^: 0.56; IQR: 0.15; CA3^NE^-CA1^E^: 0.51; IQR: 0.13; Fig. 7, G and H).

Last, given that Grm5 is not expressed in all CA1 synapses (*37*), we postulated that the higher expression profile of Grm5 in engram-to-engram synapses might reflect preferential localization of this metabotropic glutamate receptor to these synapses. To test this, we analyzed Grm5 expression and localization across the three aforementioned synapse types: i.e., CA3^T^-CA1^E^ (YFP+), CA3^E^-CA1^E^ (YFP+CFP+), and CA3^NE^-CA1^E^ (YFP+CFP−) synapses (Fig. 7, I to L). Although not reaching significance, GRM5 puncta detected in CA3^E^-CA1^E^ spines also displayed higher integrated intensity relative to CA3^NE^-CA1^E^ synapses (Grm5 puncta log median integrated intensity—CA3^T^-CA1^E^: 11.99; IQR: 4.25; CA3^E^-CA1^E^: 12.38; IQR: 4.16; CA3^NE^-CA1^E^: 11.62; IQR: 4.46; Grm5 puncta log median average intensity—CA3^T^-CA1^E^: 8.84; IQR: 0.71; CA3^E^-CA1^E^: 8.88; IQR: 0.71; CA3^NE^-CA1^E^: 8.76; IQR: 0.69; Fig. 7, J and K). Comparison between synapse subpopulations revealed that CA3^E^-CA1^E^ (YFP+CFP+) spines had a significantly higher OR of Grm5 colocalization compared to CA3^NE^-CA1^E^ (YFP+CFP−) spines (OR = 1.26), representing a 26% increase in the likelihood of preferential Grm5 localization to CA3^E^-CA1^E^ synapses (Fig. 7L). In contrast, no significant differences were observed when comparing Grm5 localization in CA3^E^-CA1^E^ (YFP+CFP+) (OR = 1.09) and CA3^NE^-CA1^E^ (YFP+CFP−) (OR = 0.87) synapses to their mixed parent CA3^T^-CA1^E^ (YFP+) population (Fig. 7L).

Together, our molecular data demonstrate that Grm5, the most significantly enriched and dominant CFC protein, is more highly expressed in and preferentially localized to labeled CA3^E^-CA1^E^ synapses. This convergence of molecular and structural data suggests a specialized role for Grm5 at synapses formed between neurons coactivated by learning.

## DISCUSSION

Experience-dependent adaptations in the protein architecture of synapses are a major determinant of information storage and recall. However, the protein landscape of engram cell synapses has remained largely unresolved because of technical limitations in identifying, isolating, and analyzing these sparse synaptic subpopulations. We made progress in overcoming these hurdles by implementing a workflow that combined activity-dependent labeling and enrichment of input-specific synapses with optimized sample preparation for low input and untargeted label-free high-throughput quantitative DIA-MS (data-independent acquisition mass spectrometry). This approach enabled us to profile the molecular content of samples enriched for CA1 engram cell synapses. In doing so, we provide a discovery-oriented proteomic resource that parallels the augmented synaptic connectivity of eGRASP-expressing CA3 synapses onto CA1 engram cells 72 hours after learning. In addition, we identified distinct protein expression signatures that dominate when contextual memories are more salient through negative valence, suggesting molecular features associated with memory strength and emotional modulation.

### Experience-dependent protein expression signatures in CA1 engram cell synapses

eGRASP expression within the CA3-CA1 circuit does not interfere with synaptic transmission (*10*) or learning (*11*), thereby providing a spatiotemporally restricted tag to label the engram cell synaptic subpopulation for morphometric and proteome analysis. Approximately 6 to 7% of CA1 synapses were eGRASP+ before sorting. Thus, global proteomic analysis would be dominated by nonengram synapses, effectively averaging out signals specific to labeled CA3 synapses onto CA1 engram cells. Synaptosome sorting resulted in a sevenfold enrichment of eGRASP+ synaptosomes, ensuring that downstream analyses were focused on this otherwise sparse population.

We directed our proteomic analysis to samples enriched for CA1 engram cell postsynaptic compartments receiving input from both engram and nonengram cells in CA3 (i.e., total CA3 input-CA3^T^-CA1^E^ synapses). In vivo longitudinal imaging of this circuit (*11*) has demonstrated that (i) CA1 neurons that become engrams after learning are preferentially preconnected to CA3 neurons that become engrams, in comparison to neurons that remain nonengrams; (ii) CFC results in the modification of preexisting synapses between these preconnected CA3 and CA1 neurons in conjunction with spinogenesis that results in new engram-engram synapses; and (iii) these newly formed synapses account for a higher proportion of the observed elevated spine density after learning. On the basis of this, we expect our CA3^T^-CA1^E^ pool to contain a substantial proportion of preexisting and newly formed CA3^E^-CA1^E^ synapses along with a relatively smaller percentage of CA3^NE^-CA1^E^ synapses. In accordance, overlap analysis determined that more than 50% of CA3^T^-CA1^E^ synapses were also CA3^E^-CA1^E^. At a molecular level, flow cytometry analysis confirmed no significant expression differences between CA3^T^-CA1^E^ and CA3^E^-CA1^E^ synapses for seven of eight proteins tested, supporting the notion of CA3^E^-CA1^E^ synapses contributing strongly to the molecular signature of CA3^T^-CA1^E^ synapses and/or that CA3^NE^-CA1^E^ and CA3^E^-CA1^E^ synapses differ in number and structure rather than in the expression levels of the assessed proteins. In line with this, engram-to-engram synapses displayed a larger volume and spine head diameter than nonengram-to-engram synapses.

We found that eGRASP-expressing engram cell synapses have a unique molecular signature when compared to unlabeled counterparts that may reflect proteome adaptations involved in recent contextual memory maintenance. Notably, we observed a small overlap between proteins regulated at CA3-CA1 engram cell synapses and previously identified plasticity modulators annotated to Gene Ontology (GO) and Reactome. Although these databases encompass plasticity-associated proteins identified across multiple model systems and time points, they likely do not capture proteins specific to the engram cell circuit or the specific time point probed here. We hypothesize that the low overlap may stem from CA3-to-CA1 circuit synapses recruiting a specific subset of proteins to maintain plasticity patterns of contextual memory 72 hours after learning. This underscores the importance of examining synapse-specific molecular diversity among different strata and circuits under both basal and activated conditions (*38*). It is also notable that although being different in identity, enriched/depleted CA1 engram cell synaptic proteins map to a very specific subset of BPs related to physiological and structural plasticity. In addition, we propose that substrates identified as differentially expressed in our dataset (e.g., Marcks, Elfn2, and Plxna4) are candidates for driving or maintaining enhanced synapse structural signatures observed after contextual learning and thus merit further functional investigation via in vivo manipulation of protein expression. Although the proteome enriched in eGRASP+ synapses was predominantly associated with glutamatergic cell types, we observed a higher expression of selected inhibitory and astrocytic markers, such as Syt2 (after CFC and CE), Nlgn2, and Gfap (after CFC). This may be due to a complex synaptic microarchitecture that includes multiple synapse boutons (*39*) and dually innervated spines contacted by both excitatory and inhibitory presynaptic terminals (*40*). These synapse types increase in number after learning or stimulation (*41*–*43*). In addition, CA1 engram cell synapses are in close apposition to astrocytic membranes, forming astrocyte-synapse interfaces (*43*). The enrichment of these proteins in our dataset may thus reflect (i) copurification of closely adjacent structures, part of a complex memory-associated synaptic microenvironment; (ii) leaky or nonexclusive *CaMKII*α-driven viral expression not strictly confined to excitatory neurons (*44*); (iii) copurification attributable to aggregation or partial enrichment after sorting; or (iv) a combination of these factors.

A direct comparison of CFC and CE groups using Huber regression and comparative dominance analyses allowed us to pinpoint differentially expressed protein signatures, relative to unlabeled comparators, that may reflect aversive contextual memory. Logistic regression, along with other statistical and distribution analyses, confirmed experience-dependent divergence in synaptic compartment specialization, establishing a dominance for postsynaptic proteins in aversive contextual fear memory. These molecular distinctions between CFC and CE at the level of the labeled CA1 engram cell postsynapse may be attributed to the longer-lasting stability of synaptic proteome adaptations when a contextual memory acquires a negative valence and therefore becomes more salient and persistent. The regulation of synaptic plasticity requires calcium homeostasis, which may be driven by the CFC-exclusive enrichment of Ca^2+^ signaling pathways and dominant expression of the Na^+^/Ca^2+^ exchanger Slc8a2, a protein known to regulate the frequency threshold of LTP in the hippocampus (*45*). The Gria2 AMPAR, an enhancer of synaptic transmission (*46*), was exclusively enriched after CFC and categorized within the CFC-dominant group. Quantitative flow cytometry confirmed Gria2 enrichment in both CA3^T^-CA1^E^ and CA3^E^-CA1^E^ synapses after CFC, mirroring the pattern of enhanced AMPAR-mediated synaptic transmission previously reported for the same two synaptic populations (*10*). Furthermore, CA1 engram cell synapses are known to exhibit LTP occlusion after CFC, an effect most prominent in CA3^E^-CA1^E^ synapses (*10*). Consistent with this, we observed a higher expression and preferential localization of Grm5, the most significantly CFC-dominant protein, in CA3^E^-CA1^E^ synapses. Whether and how this group I metabotropic glutamate receptor contributes functionally (i) to maintaining memory and underlying plasticity (*47*) at engram-to-engram synapses or (ii) in regulating engram synapse structural connectivity patterns via actin remodeling and local translation (*48*) in the days after CFC remain an important focus of future investigation. This would require engram-specific in vivo targeting of the protein combined with physiological, molecular, structural, and memory-expression readouts.

Overall, the larger contribution of CA1 postsynaptic enrichment after CFC supports the view that this subcellular compartment hosts molecular machinery critical to maintain salient learned behavior over time, while the relatively modest contribution of presynaptic proteins is in line with CA3 being more involved in rapid encoding of unified contextual information than in the longer-lasting process of memory consolidation (*8*, *49*). This perpetuation of specialized (post)synaptic function after CFC may be driven by several factors, including (i) coactivated neuromodulatory or inhibitory input onto these synapses that can sculpt synaptic connectivity in an experience-dependent manner (*15*, *50*–*52*) or (ii) nonlinear interactions between CA3 input and entorhinal inputs conveying discrete sensory information about the fear context onto the same dendrites (*51*).

It remains to be determined whether this potential postsynaptic dominance is a general feature of salient memory traces or whether it is specific to negative-valence experiences. Whether a similarly enhanced postsynaptic signature would emerge at CA1 engram cell synapses following the formation of a positive-valence contextual memory remains an open question (*53*, *54*).

### Experience-dependent enrichment of protein paralogs in CA1 engram cell synapses

An intriguing observation is the experience-dependent regulation of protein paralogs, many of which have been previously implicated in synapse strength, structural plasticity, learning and memory, paralog-specific function, and synapse diversity. For instance, DEA revealed that relative to the eGRASP− population, Dlg paralogs Dlg1 and Dlg3 were more enriched after CFC, while Dlg4 was enriched after both CFC and CE. These members of the PSD-MAGUK family play sequential roles in the maturation of nascent spines, with Dlg1 rapidly accumulating at new spines to stabilize them after high synaptic activity (*55*), as may be the case after CFC. This underscores the notion that protein paralogs arisen after two consecutive genome duplications have acquired different functional roles in supporting cognitive complexity (*30*). At the presynapse, Unc13a was enriched in both groups and Unc13b after CE. Of these, Unc13a is specifically associated with changes in synaptic strength (*56*–*58*), in line with learning-induced augmentation of presynaptic plasticity (*10*), while both of these proteins have been implicated in paralog-specific roles underlying versatile information storage in the face of varying sensory input (*59*–*61*). These combinatorial expression patterns of paralogs may support a model in which diverse synaptic proteomes are each tailored to the contextual experience being encoded, which warrants further research across multiple levels of analysis.

### Engram cell synapses of contextual memory encompass molecular determinants of cognitive phenotypes and related disorders

We demonstrate that a selection of genes enriched in labeled CA1 engram cell synapses versus unlabeled controls is identified with high confidence in normal and aberrant human cognitive functioning. For instance, a very recent analysis of PTSD loci identified the solute carrier Slc12a5 as among the top hits for PTSD vulnerability across 1,222,882 individuals of European ancestry (*62*). This K^+^-Cl^−^ cotransporter, enriched in both CFC and CE engram cell synapses, plays a critical role in inhibitory modulation of excitatory synaptic plasticity and memory formation (*63*). A possible loss-of-function variant in this gene could therefore lead to aberrant memory processing, as observed in PTSD. We also found an enrichment of GWAS/phenotypes not limited to memory engram dysfunction per se but instead related to cognitive synaptopathies (e.g., schizophrenia). These associated GWAS proteins may be involved in specialized plasticity within activated circuits relevant to such disorders/phenotypes. In addition, our finding that a subset of synaptic proteins down-regulated in the CA1/subiculum of patients with AD are enriched in eGRASP-expressing CA1 memory engram cell synapses indicates that they may serve as molecular determinants of AD-related synaptic dysfunction and memory impairment. Attributing a causal role for these proteins in cognition may help to classify them as key disease substrates with therapeutic potential.

### Limitations and future perspectives

In this study, we combined synaptosome isolation, sorting, and MS-based proteomics with DEA to identify proteomic signatures within eGRASP-labeled CA1 engram cell synapses. The incorporation of pelleting steps during synaptosome isolation and downstream flow cytometry may introduce aggregation or selective loss of material. These would consistently affect all samples used for relative comparisons. Our sorting strategy used a size-based trigger, which is inherently less sensitive than fluorescence-based triggering (*64*). Despite this, the resulting synaptosome population (i) displayed a sevenfold enrichment upon reanalysis, (ii) retained apposed pre- and postsynaptic elements, and (iii) was enriched for synaptic proteins. Nevertheless, size-gated synaptosomes likely contain higher levels of non–synapse-specific contaminants and aggregates relative to fluorescence-triggered preparations. Future efforts to incorporate eGRASP tags compatible and/or compensable with FM4-64 may enhance the sensitivity of circuit-specific synaptic subpopulation analysis.

For enrichment, depletion, and downstream dominance analyses, the eGRASP− population—gated exclusively on biophysical properties rather than fluorescence—served as a comparative baseline. While this group displays broadly overlapping proteomic composition and similar synaptic protein abundance with eGRASP+ samples, it includes a higher proportion of nonsynaptic proteins and is inherently more heterogeneous. In addition to containing a substantial fraction of CA1 nonengram synapses, this preparation also harbors putatively unlabeled CA1 engram cell synapses, a very sparse fraction of activated synapses (*65*) and a bulk of nonactivated synapses from other hippocampal areas like CA3 and DG [except mossy fiber synapses (*66*)]. The compositional heterogeneity of this comparator population should therefore be considered when interpreting enrichment and depletion patterns. Future approaches enabling fluorescence tagging of input-specific, nonactivated CA1 synapses would improve sorting purity and facilitate more synapse type–specific comparisons with labeled CA1 engram cell populations.

Because of the limited protein input obtained from sorted engram cell synaptosomes, we used flow cytometry for multiplexed validation of differential protein expression. This approach is however not fully orthogonal to the isolation and sorting strategy used for proteomic analyses. Complementary immunohistochemical analyses provided quantitative support for Grm5 localization and enrichment in engram synapse subpopulations, and future, superresolution microscopy might provide independent validation and nanoscale spatial mapping of candidate proteins to defined synapse subtypes and synaptic subdomains.

While our data offer a static snapshot into the synaptic proteome changes associated with memory processing, the temporal evolution of these expression signatures remains unknown. Investigating how synaptic states underlying contextual fear memory evolve across time and brain regions will be critical, particularly in light of (i) the transient role of hippocampal engrams in memory persistence (*67*), (ii) the dynamics of synaptic protein turnover in response to experience (*68*), and (iii) emerging evidence that the CA3-CA1 engram cell network may shift to non-Hebbian plasticity mechanisms as memory matures (*43*). Notably, the identification of proteomic signatures associated with engram cell synapses in the hours after learning, i.e., during memory formation, would require a different tagging method than the viral-TRAP system, which requires at least 24 hours, after learning and 4TM administration, for downstream vector expression (*22*).

This dataset primarily serves as an exploratory hypothesis-generating resource and identifies several discussed proteins as prime candidates for future analysis. These include proteins (i) with a unique predicted function in regulating synapse structure and strength, (ii) belonging to paralogous families, (iii) validated as having a higher expression signature in CA3^E^-CA1^E^ synapses, and (iv) with links to cognitive phenotypes and related disorders. Determining the duration of expression and establishing a functional mechanistic link between these proteins, memory expression, and synapse physiology within the CA3-to-CA1 circuit over time are crucial for causally identifying the protein landscape underlying memory maintenance.

In conclusion, our data make an important first step toward mapping input-specific protein signatures of CA1 memory-encoding synapses that parallels augmented engram cell connectivity 72 hours after contextual learning. It also highlights synaptic adaptations that are more prominent when such memories are aversive and, therefore, more salient. Beyond these insights, the repertoire of techniques used here, in combination with evolving genetic, molecular, and imaging techniques, will provide a crucial bridge between molecular and system neuroscience and may serve as a valuable platform for identifying circuit-specific vulnerabilities in synaptopathies and neurodegenerative disease.

## MATERIALS AND METHODS

### Animals

Adult male C57BL6/J mice aged 9 weeks at the start of experiment were obtained from Charles River, France. Animals were group housed upon arrival, single housed in the week before surgery, and kept under a 12-hour light-dark cycle with ad libitum access to food and water for the entire duration of the experiment. Behavioral experiments were conducted during the light phase, and mice were randomly allocated to a behavioral condition. All animal experiments are approved by the “Centrale Commissie Dierproeven” (Central Commission for Animal Experiments, protocol no. AVD11200202316866) of the Netherlands government and performed according to the Netherlands Law on Animal Research (Wet op de dierproeven) in full agreement with the Directive 2010/63/EU with local approval by and under supervision of the Animal Welfare Body of Amsterdam UMC-VU.

### Viral vectors and stereotactic microinjections

AAV-CWB-yellow pre-eGRASP(p32) [titer: 2.21 × 10^11^, Addgene plasmid no. 111580, gift from Kaang and colleagues (*10*)], AAV-EWB-DIO-cyan pre-eGRASP(p32) [titer: 5.9 × 10^10^, Addgene plasmid no. 111589, gift from Kaang and colleagues (*10*)], AAV-EWB-DIO-myrTagRFP-T-P2A-post-eGRASP [titer: 6.6 × 10^11^, Addgene plasmid no. 111581, gift from Kaang and colleagues (*10*)], AAV-*Fos*::CreERT2 [titer: 2.85 × 10^12^, gift from van den Oever and colleagues (*22*)], and AAV-EWB-DIO-yellow pre-eGRASP(p32) were generated by replacing the coding sequence of cyan pre-eGRASP with that of yellow pre-eGRASP (titer: 1.53 × 10^12^), and all constructs were all packaged as serotype 5 viruses.

For viral microinjections, mice were first anesthetized with isofluorane (1 to 3% to effect, up to 5% for induction, RB Pharmaceuticals, UK) and then transferred to a stereotactic frame. Subcutaneous injections of Lidocaine (2%, Sigma-Aldrich Chemie N.V., The Netherlands) provided topical analgesia. The yellow pre-eGRASP construct and/or a virus mixture of CreERT2 and cyan/yellow pre-eGRASP construct (ratio, 1:200; AAV-Fos::CreERT2 was injected at a final titer of 1.4 × 10^10^) were injected bilaterally into CA3 [anterior-posterior (AP): −1.9, medial-lateral (ML): ±2.35, dorsal-ventral (DV): −2.45 from bregma], while a virus mixture of CreERT2 and post-eGRASP construct (ratio, 1:200; AAV-Fos::CreERT2 was injected at a final titer of 1.4 × 10^10^) was bilaterally injected into CA1 (AP: −1.9, ML: ±1.5, DV: −1.5 from bregma). All injections were performed with microinjection glass needles and a microinjection pump (CMA/100 Microinjection Syringe Pump, Microdialysis CMA, Sweden). A total virus volume of 0.5 μl was injected at a rate of 0.1 μl/min per injection site and given an additional 8 min for diffusion. Perioperative analgesia was administered by a subcutaneous injection of Temgesic (0.1 ml per kg, RB Pharmaceuticals, UK). After surgery, mice were closely monitored and given 3 to 4 weeks for recovery.

### Contextual fear conditioning (CFC)

Mice were handled for 2 min for 2 days, 48 hours before the start of the behavioral experiment. On the day of the experiment, mice were placed in the fear conditioning chamber with a grid and walls made of stainless steel and a plexiglass door located in a soundproof box (Med Associates Inc., USA, and Noldus, The Netherlands). The box was illuminated and with constant background white noise (50 dB, 5000 Hz). Mice undergoing CFC were given 120 s to explore the context, after which three electrical foot shocks (0.7 mA, 2-s duration) were administered at an interval of 60 s. Mice were placed back into their HC 30 s after the last foot shock. The fear conditioning box was cleaned with 70% ethanol between trials.

#### *Context exposure* (*CE*)

Mice were handled as described above but were given 276 s to explore the fear conditioning chamber with no foot shock delivery.

#### HC controls

Mice were handled as described above but not exposed to the conditioning chamber and remained in the HC for the entire duration of the experiment.

#### Context fear memory retrieval

Seventy-two hours after conditioning, animals were exposed to the conditioning context in the absence of foot shocks for 120 s. Freezing behavior was automatically analyzed by Video Freeze Video Fear Conditioning Software (Med Associates Inc., US) or Ethovision XT (Noldus, The Netherlands). Freezing bouts were defined as a lack of movement except respiration at a threshold of 0.23%.

### 4HT treatment

Mice received an intraperitoneal injection of 4-TM (25 mg/kg, HB6040, Hello Bio Ltd., Republic of Ireland) solution 2 hours after the behavioral experiment, as described previously (*22*). Fifteen milligrams of 4-TM was first dissolved in 300 μl of dimethyl sulfoxide (D8418, Sigma-Aldrich, The Netherlands). This stock solution was then stepwise further diluted in 2.8 ml of saline containing 2% Tween 80 (P1754, Sigma-Aldrich, The Netherlands), and in a last step, an equal amount of saline was added.

### Immunohistochemistry

Mice were deeply anesthetized with a mixture of tribromoethanol (14:1000, Thermo Fisher Scientific, The Netherlands, 0.16 ml/kg, intraperitoneal injection), Active amyl alcohol (14:1000, Sigma-Aldrich, The Netherlands) in Milli-Q water was transcardially perfused with ice-cold phosphate-buffered saline (PBS) (pH 7.4), followed by 4% ice-cold paraformaldehyde (PFA) (pH 7.4). After removal of the brain, the tissue was postfixed in 4% PFA for 24 hours. The tissue remained in a 30% sucrose solution of PBS with 0.02% NaN_3_ until further processing. Brains were sliced at 50 μm on a cryostat and stored in PBS with 0.02% NaN_3_, of which sections from −1.6 to −2.1 mm AP to bregma were used for further analyses. For immunohistochemical staining, slices were washed three times in 1× PBS for 10 min each and then incubated in blocking solution consisting of 5% normal goat serum (Thermo Fisher Scientific, The Netherlands), 2.5% bovine serum albumin (Sigma-Aldrich, The Netherlands), and 0.25% Triton X-100 (Sigma-Aldrich, The Netherlands) in PBS at room temperature for 1 hour. Afterward, slices were incubated in blocking solution with the primary antibody anti-RFP (Rabbit, 1:1000, Tebubio BV, The Netherlands) and anti–c-Fos (1:1000, Rat, SySy, Germany) at 4°C overnight. Slices were then washed three times in 1× PBS for 10 min each and incubated in blocking solution containing anti-rabbit Alexa Fluor 568 (1:400, Goat Life Technologies, The Netherlands) and anti-rat Alexa Fluor 633 (1:400, Goat, Thermo Fisher Scientific, The Netherlands) at room temperature for 3 hours. After three washing steps of 10 min in 1× PBS, the sections were incubated in PBS with 4′,6-diamidino-2-phenylindole (DAPI; 300 nM, Invitrogen, The Netherlands), mounted on glass slides, and coverslipped with ProLong Antifade Mountant (Thermo Fisher Scientific, The Netherlands).

### Cell counting

For the quantification of cell somas, six *z*-stack images of the dorsal CA1 (1200 by 1200 pixel, 4-μm step size, 40-μm range) were obtained per mouse using a 40× objective on a Nikon HD Eclipse confocal microscope. DAPI, YFP eGRASP, boosted RFP, and Cy5 were imaged using excitation wavelengths of 405, 488, 560, and 635 nm, respectively. The number of tagged RFP+ and Fos+ somas in the pyramidal layer of the dorsal CA1 was counted manually per *z*-stack plane in FIJI (*69*) (version 2.9.0/1.53t) using the Cell counter plug-in. Somas that were present in multiple subsequent planes were only counted once. The experimenter was blinded to the experimental condition. Representative images were edited in FIJI to generate two-dimensional (2D) projections of *z*-stacks, and all images were treated identically.

### Reconstruction of dendrites and spines

Structural analysis was performed as previously described (*34*). Briefly, for reconstruction of dendrites and spines, six *z*-stack images each of the stratum oriens and stratum radiatum of the dorsal CA1 (1200 by 1200 pixel, 0.2-μm step size, 20-μm range) were recorded using a 100× objective on a Nikon Ti2 wide-field microscope using excitation wavelengths of 405, 470, and 560 nm. Images acquired using the wide-field microscope were first deconvolved using Huygens Professional (Scientific Volume Imaging BV, Hilversum, The Netherlands) and then further processed in Imaris (version 9.8, Bitplane, Zurich, Switzerland) to reconstruct spines and dendrites of the 3D volumes. To reconstruct eGRASP+ postsynaptic CA1 spines, all synapses tagged with YFP (eGRASP) were identified using the Spot Detection tool. Then, a region of interest was set around a secondary or tertiary dendrite, and the dendrite was manually tracked using the Filament Tracer tool. In a next step, all YFP+ eGRASP spots colocalizing with the RFP signal of the traced dendrite were manually identified and automatically reconstructed with the experimenter being blind to the condition. Structural data, such as spine density and spine volume, were exported from IMARIS and used for further analyses. Representative images are of 2D planes in slicer view (main figures) or 3D volumes (supplementary figures) within IMARIS.

### Statistics for spine morphometric analysis

Bar graphs display the group means ± 1 standard error of the mean. Dots are equivalent to individual data points or averages per mouse and specified in the figure legends (and table S2). For behavioral analysis and engram cell and reactivation analysis, an independent Student *t* test was conducted if assumptions of normality and homogeneity of variance were met, and a Wilcoxon rank-sum test was chosen as a nonparametric alternative in all other cases.

For morphometric analysis of spines, a generalized linear model was built in a hierarchical manner starting from a fixed intercept model over a random intercept model to a predictor model with a random intercept if the more advanced model fitted the data substantially better than the previous model. Given that morphometric data do not fulfill the assumption of independent observations of conventional statistical tests, a random intercept was chosen to account for the nested data structure (*70*). Each level was tested separately as a random intercept (mouse, slice, dendrite, mouse/slice, mouse/dendrite, slice/dendrite, and mouse/slice/dendrite), and the random intercept with the best fit was chosen on the basis of Akaike’s information criterion (*71*). The final model was then tested whether it fulfilled statistical assumptions of normality, linearity, and homogeneity of residuals, and if necessary, the fit of the model was improved by performing variable transformations or using weighted means. All statistical analyses were performed in R Studio (The R Foundation for Statistical Computing, 2023). The statistical significance threshold was defined as *P* < 0.05, and a Bonferroni correction was performed in case of multiple testing.

### Synaptosome isolation

Synaptosomes were prepared as previously described (*72*). Briefly, the dorsal hippocampus was dissected, snap-frozen on dry ice, and stored at −80°C until further use (*72*, *73*). Samples from CE (*n* = 12) and CFC (*n* = 12) groups were homogenized using a Homgenplus homogenizer (Schuett Biotec., Göttingen, Germany) in a buffer containing 0.3 M sucrose (Sigma-Aldrich, The Netherlands) and 500 mM Hepes, pH 7.4 (Sigma-Aldrich, The Netherlands), supplemented with a protease inhibitor cocktail (PIC) (Roche, The Netherlands). The homogenate was centrifuged at 1000*g* for 10 min at 4°C, and the collected supernatant was layered onto a 0.8 M/1.2 M sucrose gradient, followed by ultracentrifugation at 10,000*g* for 2 hours at 4°C. Purified synaptosomal fractions were harvested from the interface of the sucrose gradient; diluted in 5 mM Hepes, pH 7.4; and centrifuged again at 18,500*g* for 30 min at 4°C. The pelleted purified synaptosomes were resuspended in 1 ml of (0.22 μm) filtered PBS containing PIC, divided into 250-μl aliquots, and snap-frozen in liquid nitrogen for storage at −80°C before downstream analysis (*74*, *75*).

### Synaptosome sorting

Flow cytometric data acquisition and synaptosome sorting were conducted using a BD Influx instrument (BD Biosciences, US) equipped with BD FACS software for data analysis. Frozen synaptosomes were thawed on ice, protected from light, and diluted to 1 ml in filtered PBS supplemented with a PIC. To prevent clumping and aggregation of synaptosomes, 0.5% PluronicR F-68 surfactant (Thermo Fisher Scientific, The Netherlands) was added to the samples (*75*, *76*). Instrument settings included an FSC detector (with a 4-mm obscuration bar) and an SSC detector (488/10 BP). A 488-nm, 200-mW laser (530/40 nm) and a 561-nm laser (610/20 nm, 75 mW) were set for eGRASP-YFP and RFP detection, respectively. All detector voltages were determined by eight-peak bead (Invitrogen, The Netherlands) mean fluorescence intensity targets, established at the beginning of the project to account for instrument variability over time. An FSC threshold was used and set by running only PBS + PIC buffer to determine the instrument noise threshold. Synaptosomes were initially resolved on the basis of their light scattering properties (FSC and SSC). Fluorescent polystyrene beads (Spherotech, US) of various sizes (0.44, 0.88, and 1.35 μm) served as relative size approximation to produce consistent scatter gates of putative synaptosomes (*77*). Unlabeled control wild-type synaptosomes measured background autofluorescence and established gates on the basis of fluorescence tagging expression (RFP+ and YFP+) in unsorted eGRASP samples. A trigger pulse gate was established to enrich synaptosome events with a narrow pulse profile on the basis of trigger pulse width and SSC parameters. For eGRASP samples (CFC: *n* = 11; CE: *n* = 10), synapses within the trigger pulse width gate were initially gated against RFP expression, and the subset coexpressing YFP was sorted. Sorting was done with a 100-μm nozzle at 15 psi (103.42 kPa), a piezo amplitude of 6 to 10 V, and a frequency of 25 to 27 kHz/s with a one-drop purity phase mask. The pressure differential was optimized to maintain the acquisition of 6000 to 8000 events/s, collecting up to 500,000 sorted events in a protein low-bind 1.5-ml tube (Eppendorf, Germany). Samples were snap-frozen and stored at −80°C until further use. Sorting efficiency was assessed by reanalyzing a fraction of eGRASP+ sorted sample (5000 events) with its unsorted counterpart. Plots were generated using FlowJo version 10 (BD Biosciences, US).

### Synaptosome immunofluorescence

Synaptosome immobilization on glass coverslips was performed as described previously (*74*). Glass coverslips (diameter: 13 mm; Precision cover glasses thickness no. 1.5 H, Marienfeld, Germany) were initially coated with 5% bovine serum albumin (Sigma-Aldrich, The Netherlands) and left overnight at 4°C. The following day, an equivalent of 250,000 sorted synaptosomes (eGRASP+/−) from CFC (*n* = 5) and CE (*n* = 5) groups were thawed on ice and subsequently diluted with PBS, 0.5% Pluronic F-68 membrane dye, and PIC. Diluted synaptosomes were then centrifuged at 4000 rpm for 40 min at 4°C onto coated coverslips. After immobilization, the synaptosomes on coverslips were fixed using 4% PFA (Sigma-Aldrich, The Netherlands) in PBS for 15 min at 4°C, followed by an additional 45 min at room temperature. Excess PFA was quenched with 100 mM NH_4_Cl for 30 min, followed by a wash with filtered PBS. Next, synaptosomes were blocked for 30 min at room temperature with blocking solution containing PBS, 5% normal goat serum (Thermo Fisher Scientific, The Netherlands), 2.5% bovine serum albumin (Sigma-Aldrich, The Netherlands), and 0.2% Triton X-100 (Sigma-Aldrich Chemie N.V., The Netherlands). Subsequently, samples were incubated for 1 hour at room temperature with primary antibodies: anti-Syp (1:500, Guinea pig, cat. no. 101004, SySy), anti-Grin2a (1:500, Rabbit, cat. no. ab124913, Abcam), anti-Dlg4 (1:500, Mouse, cat. no. MA1-046, Invitrogen), anti-vGlut1 (1:1000, Mouse, cat. no. 135011, SySy), anti-Homer1 (1:1000, Guinea pig, cat. no. 160004, SySy), anti-Bsn (1:500, Mouse, cat. no. SAP7F407, Enzo Life Sciences), and anti-Map2 (1:2000, Chicken, cat. no. AB5543, Sigma-Aldrich) diluted in blocking solution, followed by a 1-hour incubation with secondary antibodies Alexa Fluor 647 (AF647) (1:400, Goat Anti-Guinea Pig, cat. no. A-21450, Invitrogen), Alexa Fluor 568, (1:400, Goat Anti-Rabbit, cat. no. A-11011, Invitrogen), Alexa Fluor 568 (1:400, Goat Anti-Mouse, cat. no. A-11004, Invitrogen), AF647 (1:400, Goat Anti-Chicken, cat. no. A-21449, Invitrogen), and Alexa Fluor 633 (1:400, Goat Anti-Rabbit, cat. no. A-21070, Invitrogen) diluted in same blocking solution. After a final wash with PBS, coverslips were mounted on glass microscope slides (VWR, The Netherlands) using ProLong Glass Antifade Mountant (Thermo Fisher Scientific, The Netherlands) and dried in the dark at room temperature overnight. The following day, samples were either imaged or transferred to −20°C for storage.

### Rescan confocal microscopy of synaptosomes

High-resolution representative images of single synaptosomes were acquired using a Re-scan Confocal Microscopy system, consisting of an Olympus IX81 microscopy body and a rescan unit (Confocal.nl., The Netherlands) and equipped with a 60×, 1.42–numerical aperture (NA) oil objective. Images were captured on a Retiga LUMO charge-coupled device camera chip (Teledyne Qimaging). Multichannel *z*-stacks were obtained for single synaptosomes with a step size of 123 nm. The eGRASP-YFP tag was imaged with a laser excitation wavelength of 488 nm (emission wavelength: 520), while immunohistochemical stains were imaged with excitation wavelengths of 561 nm (emission wavelength: 595) and 640 nm (emission wavelength: 700). After imaging, the images were processed using Huygens Professional (SVI, version 21.10). Deconvolution was performed using an experimentally measured point spread function obtained by imaging 0.2-μm TetraSpeck Microspheres (Thermo Fisher Scientific, The Netherlands) on the same system. The between-channel chromatic aberration was determined using the 0.2-μm TetraSpeck Microspheres (Thermo Fisher Scientific, The Netherlands), using the Chromatic Aberration Corrector of Huygens Professional to assess and correct for center-to-center differences between each channel. Last, all images were cropped and further processed using FIJI (ImageJ, version 2.3.0/1.53q).

### Confocal microscopy of synaptosomes

Imaging of synaptosomes for colocalization analysis was conducted through laser scanning confocal microscopy using a Nikon Eclipse Ti inverted microscopy system with an A1R confocal module for image acquisition. An apochromatic 60× oil 1.40-NA objective lens was used, and images were captured using excitation lasers at 488, 561, and 635 nm. Corresponding emission spectra were recorded with detectors set to 500 to 550, 570 to 616, and 663 to 738 nm, incorporating a pinhole size of 1.2 Airy Units.

### Synaptosome colocalization analysis

The colocalization of pre- and postsynaptic markers was assessed using SynaptosomesMacro version 1.0 (*26*), which automated image preprocessing and identified local maxima of synaptosomes under the assumption that all stained markers were in proximity. A gallery view of detected synaptosomes was generated, followed by manual selection by two independent experimenters to exclude regions with (i) additional particles, (ii) aggregation, (iii) poor focus, or (iv) proximity to image borders. Background-corrected integrated intensities were measured for each selected synaptosome. To improve the statistical rigor, quadrant borders were defined using baseline intensity thresholds derived from the intensity distributions of each marker: Q1 (low pre- and postsynapse, baseline/nonsynaptic), Q2 (low presynapse, high postsynapse), Q3 (high pre- and postsynapse, colocalization quadrant), and Q4 (high presynapse, low postsynapse). Spearman correlation analysis within each quadrant evaluated colocalization between pre- and postsynaptic markers. KDE plots visualized intensity distributions for each marker, and the proportion of synaptosomes in each quadrant was calculated to quantify synaptic population with intact pre- and postsynaptic component (Q3). Correlation, KDE, and quadrant analyses were conducted in Python environment (IDLE version 3.12.0) using the following packages: pandas (version 2.2.3) and NumPy (version 2.1.2) for numerical operations; scipy.stats (version 1.14.1) for Spearman correlation; seaborn (version 0.13.2) for visualizing correlations, regression plots, and KDEs; and matplotlib (version 3.9.2) for scatterplots, density plots, and pie charts.

### MS sample preparation

eGRASP+ and eGRASP− samples (CFC: *n* = 11 mice; CE: *n* = 10 mice, where one mouse equals one sample) were processed together in a single session using sTRAP to minimize sample loss before MS (*17*), with some minor adaptations. Snap-frozen eGRASP sorted synaptosomes were thawed on ice and precipitated by centrifugation at 18,000*g* for 30 min at 4°C, and the supernatant was discarded. Proteins were extracted and reduced in 6% SDS buffer, containing 50 mM tris-HCl (pH 8) and 5 mM tris(2-carboxyethyl)phosphine, by incubation at 55°C for 15 min in a thermomixer (Eppendorf, Germany), set to 1250 rpm. Free sulfhydryl groups were alkylated by incubation with 20 mM methyl methanethiosulfonate (200 mM stock solution) for 30 min at room temperature. Next, samples were acidified by addition of 1.1% phosphoric acid (12% stock solution), mixed with six volumes of binding/washing buffer (90% methanol and 100 mM tris-HCl, pH 8), and loaded onto a plasmid DNA microspin column (HiPure from Magen Biotechnology, China). The protein suspension was retained in the column following centrifugation at 1400*g* for 1 min, and the columns were washed four times with binding/washing buffer. Columns were transferred to new protein LoBind tubes (Eppendorf, Germany), supplemented with 100 ng of trypsin/Lys-C (Promega, US) in 50 mM NH_4_HCO_3_, and incubated overnight at 37°C in a humidified incubator. Tryptic peptides were eluted and pooled by subsequent addition of 50 mM NH_4_HCO_3_, 0.1% formic acid, and 0.1% formic acid in acetonitrile. Collected peptides were dried by SpeedVac and stored at −80°C.

### Liquid chromatography–MS (LC-MS) analysis

Each sample of tryptic digest from eGRASP+ and eGRASP− sample groups was redissolved in 0.1% formic acid and loaded onto an Evotip Pure (Evosep, Denmark) for subsequent MS analysis in a single session. Load levels from eGRASP samples were normalized against a Pierce HeLa protein digest standard (Thermo Fisher Scientific, The Netherlands) and represented 20 ng of peptides. As previously described (*78*), peptide samples were separated by 30 standardized samples per day method on the Evosep One liquid chromatography system using a 15 cm–by–50 μm reverse-phase column packed with 1.5-μm C18-beads (EV1137 from Evosep, Denmark) connected to a 20-μm-inner-diameter ZDV emitter (Bruker Daltonics, Germany). Peptides were electrosprayed into a timsTOF Pro2 mass spectrometer (Bruker Daltonics, Germany) equipped with a CaptiveSpray source and measured with the following settings: scan range of 100 to 1700 mass/charge ratio (*m*/*z*), ion mobility of 0.65 to 1.5 V·s/cm^2^, ramp time of 100 ms, accumulation time of 100 ms, and collision energy decreasing linearly with inverse ion mobility from 59 eV at 1.6 V·s/cm^2^ to 20 eV at 0.6 V·s/cm^2^. Operating in dia-PASEF mode, each cycle took 1.38 s and consisted of 1 MS1 fμLl scan and 12 dia-PASEF scans. Each dia-PASEF scan contained two dia-PASEF isolation windows, in total covering 300 to 1200 *m*/*z* and ion mobility of 0.65 to 1.50 V·s/cm^2^. dia-PASEF window placement was optimized using the py_diAID tool (*79*). Ion mobility was autocalibrated at the start of each sample (calibrant *m*/*z*, 1/K0: 622.029, 0.992 V·s/cm^2^, 922.010, 1.199 V·s/cm^2^, 1221.991, 1.393 V·s/cm^2^).

### MS data analysis

As previously described (*78*), dia-PASEF raw data were processed with DIA-NN 1.8.1 (*18*). An in silico spectral library was generated from the UniProt mouse reference proteome (SwissProt and TrEMBL, release 2023-01) using trypsin/P digestion and at most one missed cleavage. Fixed modification was set to β-methylthiolation(C), and variable modifications were oxidation(M) and N-terminal M excision (at most one per peptide). The precursor charge range was set to 2 to 4, the precursor *m*/*z* range was limited to 280 to 1220, both MS1 and MS2 mass accuracy values were set to 15 parts per million, scan window was set to 8, and double-pass mode and match between runs were enabled. Retention time–dependent cross-run normalization was enabled with the robust liquid chromatography quantification strategy. Protein identifiers (isoforms) were used for protein inference. All other settings were left as default. Downstream analysis of DIA-NN results was carried out using MS-DAP 1.0.5 (*80*). Differential expression was performed using the paired-sample design matrix, explicitly reflecting the within-mouse pairing of eGRASP+ versus eGRASP− synaptosomes in each condition (CFC or CE). Mouse ID was included as a covariate to correctly model the subject-specific dependency structure. A total of *n* = 8 paired CFC samples (eGRASP+ and eGRASP−, one mouse equals one sample) and *n* = 9 paired CE samples (eGRASP+ and eGRASP−) passed quality control (sample processing, chromatogram failure, and improper normalization) and were used for further analysis. Two independent quantification pipelines were performed. The first analysis used DIA-NN output without modification of the quantified peptide set. In a second, filtered analysis, tryptic peptides from both pre- and post-eGRASP sequences (*10*) were generated computationally (7 to 40 amino acids, ≤1 missed cleavage; cleaver package), and all peptides matching these were filtered and removed postquantification, while the original FASTA/decoy setup was retained to preserve consistent FDR estimation across both pipelines. Peptide-level filtering was configured to retain only peptides that were confidently identified in at least 50% of the samples per sample group. Missing values were treated as MCAR (missing completely at random) throughout preprocessing. Peptide abundance values were normalized using the VSN algorithm, followed by protein-level mode-between normalizations. DEA was performed using the MSqRob algorithm modeling paired designs per contrast. Resulting *P* values were adjusted for multiple testing using the Benjamini-Hochberg FDR procedure. Proteins were considered significantly differentially expressed at *q* < 0.01. For each protein, log_2_ FC and effect size are reported, with effect size defined as log_2_ FC scaled to the mean abundance variance across biological replicates.

### EWCE analysis

Expression Weighted Cell-type Enrichment (EWCE) analysis (*28*) was conducted using the EWCE (version 1.12.0) R package to determine whether the identified proteins or significantly enriched proteins across CFC and CE were more highly expressed in specific cell types than would be expected by chance. Single-cell gene expression data for the hippocampus were obtained from the Allen Brain Atlas (*81*), which included data from 82,498 cells from hippocampal dissections, excluding cortical layer–specific cell types. Bootstrapping was performed with 10,000 repetitions using either a custom background of all detected proteins or the standard EWCE background of human-mouse ortholog genes. Multiple testing correction was performed using the Benjamini-Hochberg FDR correction procedure with significance defined as adjusted *P* (*P*_adj._) < 0.05.

### MS data interpretation

MSDAP 1.0.5 (*80*) was used to determine quality control metrics such as the total number of identified and quantified proteins across samples, coefficient of variation % within and across sample groups, principal components analysis, and protein abundance of sample groups. DEA analysis after filtering of eGRASP-derived peptides was performed for downstream data interpretation.

Panther classification system version 19.0. (*82*) and UniProt (release 2024_02) were used to annotate regulated proteins to different classes. Subcategories based on function were assigned for data filtering purposes. The complete classification system can be found in data S5.

ShinyGO version 0. 85 (*83*) gene ontology enrichment analyses of KEGG (Kyoto Encyclopedia of Genes and Genomes) pathways (*84*) and cellular components (CCs) were performed independently on all differentially up- and down-regulated proteins (eGRASP− versus eGRASP+) from CFC and CE groups with an FDR cutoff of *q* < 0.05 and a minimum pathway size of three proteins.

SynGO version 1.2 (*27*) analyses were performed with default settings to determine the coverage of SynGO-annotated proteins from the list of all identified proteins across CFC and CE groups. SynGO-CC and BP analysis was performed to map proteins to specific subcompartments and function.

Synaptic plasticity modulators were obtained from (i) the Reactome pathway of LTP and (iii) GO (GO-0048167): Regulation of synaptic plasticity. From 415 proteins, 194 had a SynGO annotation and were used for overlap analysis with SynGO-annotated CFC- and CE-regulated proteins.

Protein paralogs of differentially expressed eGRASP SynGO proteins were identified using the Ensembl genome database. The background list for KEGG enrichment contained all proteins used for DEA. For comparison to the AD dataset, SynGO-annotated and differentially expressed CFC and CE proteins were compared to SynGO-annotated and differentially expressed proteins described in (*32*).

### Phenome-wide association study

A PheWAS and gene lookups in online databases were carried out to characterize the broader biological role of dominant proteins in human phenotypes. The 18 dominant proteins in the CFC condition and 15 dominant proteins in the CE condition were selected. Using the corresponding human gene names for these protein products, GWASatlas (https://atlas.ctglab.nl/PheWAS) (*85*) was used to conduct PheWAS for each of these genes. This tool reports the phenotypes and associated functional domains for which robust gene-based associations (adjusted *P* < 0.05/18,476 genes = 2.706 × 10^−6^) have been previously identified in human GWAS results. Phenotypes related to cognitive functioning or cognitive disorders were highlighted.

### GWAS enrichment analysis

To test the enrichment of memory-related genes in GWAS traits, well-powered traits related to intelligence and memory from the GWAS catalog, as well as synaptopathies and dementia-related phenotypes from the Psychiatric Genetics Consortium, were selected. Positional mapping of genes from GWAS summary statistics was performed using FUMA (Functional Mapping and Annotation of GWAS). Genes mapped by FUMA that were in the top 10% of genes prioritized by PoPS (polygenic priority score) were considered implicated by GWAS. Given that the latest PTSD summary statistics were not yet publicly available, genes with the top 10% of prioritization scores, as generated by the original authors of the PTSD GWAS, were considered implicated. Enrichments were calculated using Fisher’s exact test, considering a background of 18,384 potential genes that could be prioritized by PoPS and FUMA. Multiple testing correction was performed using the Benjamini-Hochberg FDR correction procedure with significance set at *P*_adj*.*_ < 0.05.

### Rare variants

Associations to single-gene Mendelian disorders were manually curated from the VarElect tool (*86*) of the GeneCards Suite and OMIM database (https://omim.org/) for disorders related to cognitive/intellectual phenotypes or neurological phenotypes with cognitive/intellectual/memory symptoms. A one-tailed hypergeometric test was used to test whether the proportion of genes linked to these same phenotypes was significantly higher for the CFC and CE experimental groups than for the nonregulated control dataset, with significance set at *P* < 0.05. The nonregulated control dataset consisted of the 100 least differentially expressed proteins common to both the CFC and CE experimental groups.

### Huber regression and dominance analysis

To quantify the predominant expression of proteins between CFC and CE groups, a robust Huber regression analysis (*36*) was first performed using CFC (independent variable) and CE effect sizes (dependent variable) derived from the DEA results of eGRASP+ versus eGRASP− within CFC and CE. The robustness of the correlation was evaluated using Pearson’s correlation coefficient. Proteins were classified as CFC-dominant if higher observed effect sizes in CFC relative to the predicted CE values, positive effect size differences, and significant *q* values (<0.01) in CFC were observed. Similarly, proteins were classified as CE-dominant if higher observed effect sizes in CE relative to the predicted CE values, negative effect size differences, and significant *q* values (<0.01) in CE were identified. Only SynGO-annotated dominant proteins were included to explore their functional specialization. Dominance scores were calculated as the difference in effect sizes between CFC and CE, and 95% CIs for predicted CE effect sizes were computed using Huber regression with 5000 bootstrap resampling iterations. SynGO-CC annotations were applied to categorize proteins into four groups: “Presynapse,” “Postsynapse,” “Both” (Presynapse and Postsynapse), and “Synapse.”

A chi-square test of independence was conducted to assess whether the observed dominance (CFC or CE) was significantly associated with synaptic location categories. To investigate the functional specialization of dominant SynGO proteins, the distribution and median of dominance scores (CFC versus CE) across different SynGO synaptic location categories were compared using KDE. The density distribution of dominance scores for all quantified proteins in CFC and CE groups was used as a control to represent the overall proteome distribution. A K-S test was performed to determine whether the distribution of dominance scores for dominant SynGO proteins significantly deviated from the control distribution.

A logistic regression model was tested to calculate the OR for the likelihood of proteins being associated with specific synaptic compartments (independent variable) on the basis of their dominance classification (dependent variable). CIs were calculated, ORs were log transformed, and statistical significance was assessed to evaluate whether the odds of specific localization categories differed from the expected baseline (OR = 1). Last, PPI networks of CFC- and CE-dominant SynGO proteins were constructed using STRING version 12.0 (*87*), applying a high stringency score of 0.7.

All analyses were conducted in a Python environment (IDLE version 3.12.0). The following Python packages were used: pandas (version 2.2.3) and NumPy (version 2.1.2) for data processing and calculation of effect sizes and dominance scores; scipy.stats (version1.14.1) for performing chi-square, K-S tests, and Pearson’s correlation; statsmodels (version 0.14.4) for Huber regression and logistic regression analysis; and seaborn (version 0.13.2) and matplotlib (version 3.9.2) for data visualization, including KDE and forest plot. Significance for statistical tests was set at *P* < 0.05.

### Immunolabeling for flow cytometry

CA3^T^-CA1^E^ only (Fig. 3) and CA3^T^-CA1^E^ and CA3^E^-CA1^E^ (Fig. 7) connections were labeled with yellow eGRASP in different cohorts of animals. Viral microinjections, behavior experiment, and synaptosome preparations were performed as described above. The flow cytometry analysis protocol for labeling fixed pellets of synaptosomes was performed as described before for synaptoneurosomes (*19*). Biochemical isolation of synaptosomes from dorsal hippocampi of CFC (eGRASP+ versus eGRASP−: *n* = 5; CA3^T^-CA1^E^ versus CA3^E^-CA1^E^: *n* = 8) and CE (eGRASP+ versus eGRASP−, *n* = 5) mice was performed. Isolated frozen pelleted (Fig. 3) or gradient (Fig. 7, A to D) synaptosomes were first thawed on ice and then centrifuged at 15,000*g* at 4°C for 10 min. Subsequently, they were resuspended in 0.1% PFA in 1× PBS, thoroughly vortexed, and rotated on an end-over-end mixer at 4°C for 15 min. After another round of centrifugation at 15,000*g* and 4°C for 30 min, the resulting pellets were resuspended in 1 ml of 0.2% Tween 20 in PBS, followed by additional vortexing and rotation at 4°C for 30 min. The tubes were then centrifuged at 15,000*g* at 4°C for 7 min. Next, fixed synaptosome pellets were resuspended in 500 μl of 5% dimethyl sulfoxide in PBS and divided into 100-μl aliquots. These aliquots were frozen overnight at 20°C and then subjected to slow freezing in a Styrofoam box at −80°C. The samples were finally stored at −80°C. For immunolabeling, thawed aliquots of fixed synaptosome pellets were divided into equal parts. The primary antibodies anti-Dlg4 (1:100, Mouse, cat. no. MA1-046, Invitrogen), anti-Gria2 (1:100, Rabbit, cat. no. ab133477, Abcam), anti-Dlg3 (1:250, Rabbit, cat. no. PA5-29116, Invitrogen), anti-Unc13B (1:180, Rabbit, cat. no. 126203, SySy), anti-Ap2B1 (1:100, Rabbit, cat. no. ab220778, Abcam), anti-Unc13A (1:140, Rabbit, cat. no. 126103, SySy), anti-Grm5 (1:100, Mouse, cat. no. MA5-24185, Invitrogen), anti-Slc12a5 (1:100, Rabbit, cat. no. Ab259969, Abcam), anti-Dbn1 (1:100, Rabbit, cat. no. 10260-1-AP, Proteintech), anti-Gnao1 (1:100, Rabbit, PA5-26142, Invitrogen), Rabbit immunoglobulin G (IgG) isotype (1:100, Rabbit, cat. no. 08-6199, Invitrogen), and Mouse IgG isotype (1:100, Mouse, cat. no. 10400C, Invitrogen) were added to each tube, and the final volume was adjusted to 300 μl with a PBS solution. The tubes were then incubated on an end-over-end mixer at 4°C for 30 min. After incubation, samples were subjected to centrifugation at 15,000*g* and 4°C for 10 min, and the resulting pellets were washed using 500 μl of PBS. Following the wash, pellets were spun down, and secondary antibodies Anti-Rabbit (1:2000, Goat, AF647, cat. no. ab150079, Abcam) and Anti-Mouse (1:400, Goat, AF647, cat. no. A-21235, Invitrogen) were added to each tube and incubated on an end-over-end mixer operating at 20 rpm and 4°C for 15 min. Pellets were then spun down at 15,000*g* and 4°C for 7 min. Last, all the samples were washed with 1 ml of PBS before resuspension in filtered PBS supplemented with Pluronic PF-68 for flow cytometry analysis.

### Flow cytometric analysis for differentially expressed proteins

Validation of MS-led differentially expressed proteins and comparative flow analysis of CA3^T^-CA1^E^ versus CA3^E^-CA1^E^ were conducted using the BD Influx cytometer equipped with BD FACS acquisition software following the same workspace and gating strategy designed during synaptosome sorting of eGRASP-YFP–tagged synaptosomes, with minor adjustments. Detection of RFP used the 561-nm, 65-mW (BP 610/20) laser, while expression for YFP was checked with the 488-nm, 200-mW (BP 530/40) and 445-nm, 80-mW (BP 480/40) lasers. For fluorescent immunolabels, the 640-nm laser (670/30) operated at 100 mW was used. Before analysis, titration experiments were performed for each antibody to optimize the final antibody concentration for immunolabeling of synaptosomes. Thresholds for immunolabel expression were set using samples stained solely with the secondary antibody (AF647) or no antibody. We gated eGRASP+ synaptosome events as subsets of immunolabeled AF647+RFP+ CA1 engram cells using RFP+YFP+ fluorescence, while eGRASP− events were gated as a subset of AF647+ immunolabeled synaptosome events. During data acquisition, 100,000 (eGRASP+ versus eGRASP−, *n* = 5) or 500,000 (CA3^T^-CA1^E^ versus CA3^E^-CA1^E^, *n* = 8) events were recorded from every unsorted eGRASP sample. To measure the background fluorescence resulting from nonspecific isotype host binding, IgG control for each group was prepared by pooling a small fraction of every animal from each group and staining with a rabbit or mouse IgG antibody. For validation experiments (Fig. 3), the corrected median intensity of immunolabels from eGRASP+ synaptosome events was compared to that of eGRASP− synaptosome events. FC of regulation was calculated using median intensities of immunolabeled synaptosomes from the eGRASP+/eGRASP− gates. For analysis of protein signatures in CA3^T^-CA1^E^ and CA3^E^-CA1^E^ synapses after CFC (Fig. 7, A to D), the corrected median intensity of immunolabels from synaptosome events of each synaptic subpopulation was compared to each other. Sample comparisons between CA3^T^-CA1^E^ and CA3^E^-CA1^E^ eGRASP+ samples after CFC were performed using an unpaired *t* test. Significance for statistical tests was set at *P* < 0.05. One animal from the CA3^E^-CA1^E^ group was excluded from analysis for Dlg3, Dlg4, Unc13a, and Gnao1 because of very low antibody labeling, resulting in an insufficient number of immunolabeled events relative to its CA3^T^-CA1^E^ counterpart. Plots were generated using FlowJo version 10 (BD Biosciences, US). The associated figure depicts log-transformed intensity values.

### Statistics for molecular analyses

Statistical analyses were conducted using GraphPad Prism version 10 and Python package scipy.stats (version 1.14.1). Specific details regarding sample size (*n*), statistical tests, and exact *P* values, adjusted *P* values (*P*_adj._), or *q* values are provided in the figure legends. Sample comparisons within CFC and CE groups (Fig. 3) were executed using one-tailed or two-tailed paired *t* tests, depending on whether the direction of regulation was known or not. In instances of non-Gaussian distribution of data, a nonparametric paired test, the Wilcoxon signed-rank test (within-group), was used. Significance for all statistical tests was set at *P* < 0.05, unless stated otherwise. A summary of statistics is provided in table S2.

### CA3^T^-CA1^E^/CA3^E^-CA1^E^ overlap, morphometry, and Grm5 expression/localization analysis

CA3^T^-CA1^E^ and CA3^E^-CA1^E^ connections were colabeled with yellow and cyan eGRASP, respectively, within the same animal. Viral microinjections, CFC, slice preparation, and immunohistochemistry were performed as described above. Sections were immunolabeled using an anti-Grm5 (1:500, rabbit, SySy, Germany) primary antibody combined with an anti-rabbit AF647 (1:400, Goat, Thermo Fisher Scientific, The Netherlands) secondary antibody. Dendrites were imaged from the stratum oriens and stratum radiatum of the dorsal CA1 using a 63× 1.40-NA oil objective on a Leica SP8 confocal microscope. Image stacks were collected at 3.7× zoom to acquire with a pixel size of 48.75 nm, and 40 images were taken per stack at 0.15-μm intervals. Stacks were acquired in two separate sequences for cyan eGRASP/AF647 and yellow eGRASP/myrTagRFP-T to prevent cross-talk using excitation wavelengths of 405, 653, 470, and 555 nm and detection windows of 420 to 500, 658 to 775, 520 to 540, and 575 to 760 nm, respectively. Images were deconvolved with an experimentally measured point spread function and corrected for chromatic aberration using Huygens Professional (Scientific Volume Imaging BV, Hilversum, The Netherlands) on the basis of processed images of 0.2-μm TetraSpeck Microspheres (Thermo Fisher Scientific, The Netherlands) acquired on the same system. Dendrites were then manually traced using the Filament Tracer tool of Imaris (version 10.2, Bitplane, Zurich, Switzerland) on the basis of RFP expression only, with the experimenter blinded to the other channels. Cyan eGRASP (CFP), yellow eGRASP (YFP), and Grm5 spots were automatically detected using the Imaris Spot Detection Tool, and engram dendrite–associated spots were subset on the basis of the spot shortest distance to the filament below 200 nm for Grm5 spots and 500 nm for cyan/yellow eGRASP spots. Representative images are of 3D volumes within Imaris of traced filaments and engram dendrite–associated spots. Spot localization within spines was then assessed using an R script where spots were registered to spines when the Euclidean distance between the spine terminal *xyz* position and the spot *xyz* position was below 700 nm. Overlap analysis of cyan and yellow eGRASP signals was performed by counting the number of spines positive for cyan eGRASP, yellow eGRASP, and both and calculating colocalization (%) per dendrite. Individual spines were classified on the basis of the eGRASP signal: CA3^T^-CA1^E^ spines were identified by yellow fluorescence (YFP+), with this parent population subdivided into CA3^E^-CA1^E^ (YFP+CFP+) and CA3^NE^-CA1^E^ (YFP+CFP−) subpopulations on the basis of the presence or absence of cyan fluorescence. The spine morphometry analysis was performed using hierarchical generalized linear mixed effect models (GLMM) as described in previous sections, with additional post hoc testing for pairwise comparisons between the three spine populations. Grm5 preferential localization to spine subpopulations was assessed using a hierarchical logistic mixed effect model, and ORs with 95% CIs were extracted post hoc for each comparison. For the Grm5 puncta intensity analysis, spines with Grm5 puncta detected were subset, intensity values were log transformed, and differences between populations were analyzed using GLMM. Statistical significance was assessed at α = 0.05 with a Bonferroni correction applied for the multiple comparisons.

## Supplementary Materials

### The PDF file includes:

Figs. S1 to S10 Tables S1 and S2 Methods Legends for data S1 to S14 Legend for supplementary source data References
