# Supplementary material for: GRASPing experience-dependent protein expression signatures enriched for hippocampal engram cell synapses
Source: Sci Adv. 2026 May 15;12(20):eadv3557. doi: 10.1126/sciadv.adv3557 (PMC13178557; doi:10.1126/sciadv.adv3557)
Supplement: Supplementary file 1 — Figs. S1 to S10 Tables S1 and S2 Methods Legends for data S1 to S14 Legend for supplementary source data References [file sciadv.adv3557_sm.pdf]

Science Advances
AAAS

Supplementary Materials for

GRASPing experience-dependent protein expression signatures enriched for hippocampal engram cell synapses

Biswajit Moharana *et al.*

Corresponding author: Priyanka Rao-Ruiz, p.rao@vu.nl

Sci. Adv. **12**, eadv3557 (2026)
DOI: 10.1126/sciadv.adv3557

The PDF file includes:

Figs. S1 to S10
Tables S1 and S2
Supplementary Methods
Legends for data S1 to S14
Legend for supplementary source data
References

Other Supplementary Material for this manuscript includes the following:

Data S1 to S14
Supplementary source data

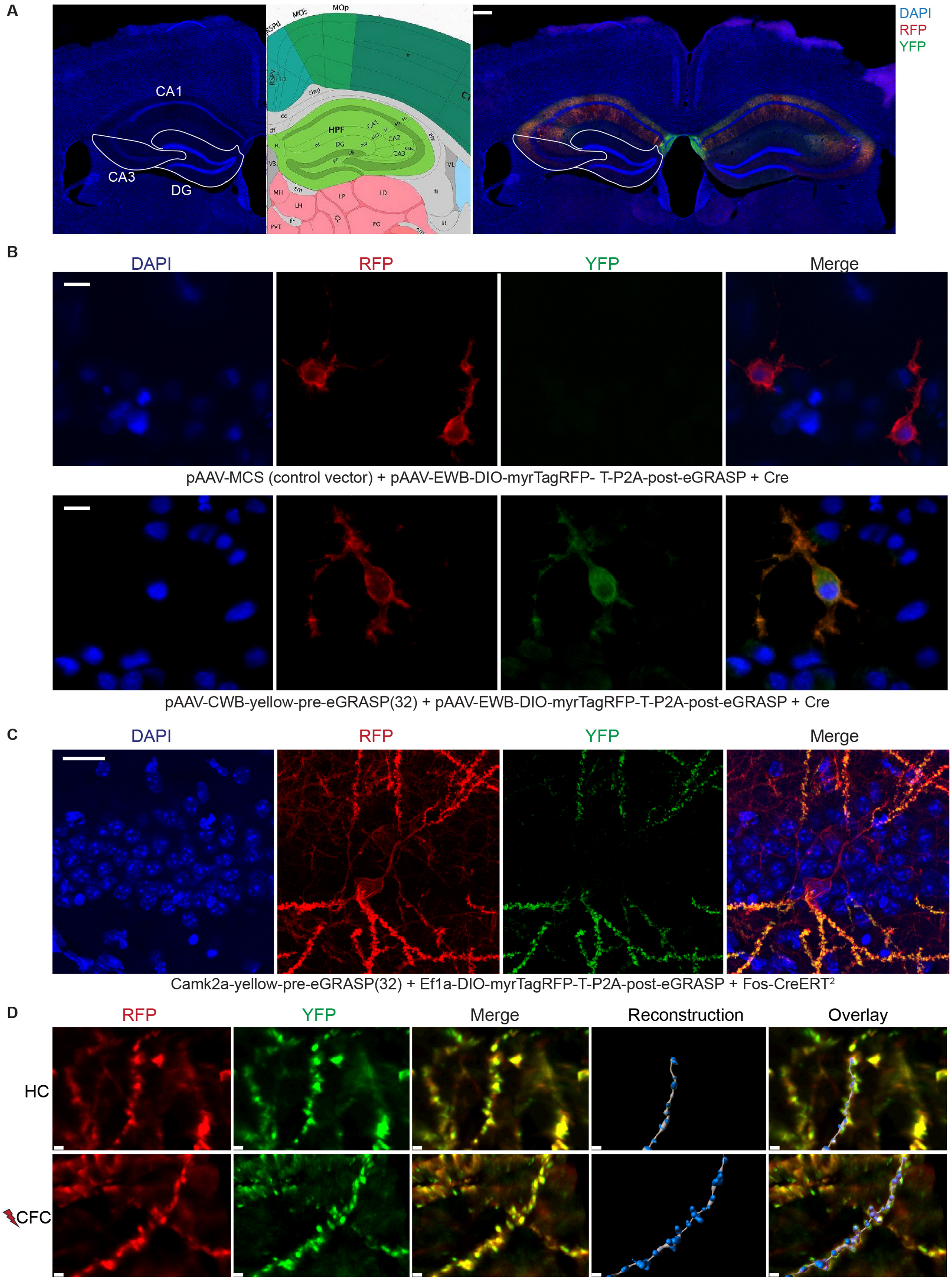
A
CA1
CA3
DG
DAPI
RFP
YFP
B
DAPI
RFP
YFP
Merge
pAAV-MCS (control vector) + pAAV-EWB-DIO-myrTagRFP- T-P2A-post-eGRASP + Cre
pAAV-CWB-yellow-pre-eGRASP(32) + pAAV-EWB-DIO-myrTagRFP-T-P2A-post-eGRASP + Cre
C
DAPI
RFP
YFP
Merge
Camk2a-yellow-pre-eGRASP(32) + Ef1a-DIO-myrTagRFP-T-P2A-post-eGRASP + Fos-CreERT2
D
RFP
YFP
Merge
Reconstruction
Overlay
HC
CFC

Fig. S1. Expression of RFP + YFP (eGRASP) in CA1 engram cells.

(A) Representative image of the dorsal hippocampus bilaterally injected with pre- and post eGRASP constructs into the CA1 and CA3 respectively, with anatomical annotations from the Allen Reference Atlas – Mouse Brain (https://atlas.brain-map.org/) (*89)* at the same slice position. DAPI (blue), RFP (red), reconstituted YFP (green) and RFP + YFP (yellow). Scale bar: 500 µm. **(B)** Representative image of Neuro2A cells co-transfected with EWB-DIO-myrTagRFP-T-P2A-post-eGRASP and Cre, and either a control vector (upper panel) or CWB-yellow pre-eGRASP(p32) (lower panel). DAPI (blue), RFP (red), YFP (green). Co-expression of pre- and post- constructs results in reconstitution within the same cell and YFP expression in the cell somata (lower panel). Scale bar: 20 µm. **(C)** Representative image demonstrating myristoylated RFP and reconstituted YFP expression in the CA1 of the dorsal hippocampus. While RFP labels the entire cell membrane including the soma, the YFP signal is preferentially located on CA1 engram cell dendrites. No reconstituted YFP signal observed in cell soma, ruling out co-expression of both constructs within the same CA1 neuron. DAPI (blue), RFP (red), YFP (green) and RFP + YFP (yellow). Scale bar: 20 µm. **(D)** Representative widefield deconvolved images showing RFP (red), YFP (green) and RFP + YFP (yellow) in the merged image, followed by the semi-automatically reconstructed dendrite (grey) and spines (blue) in IMARIS, and lastly the overlay of the original fluorescent signal and the reconstructed signal – for the home cage (HC) group (upper panel) and the contextual fear conditioned (CFC) group (lower panel). Scale bar = 2 µm.

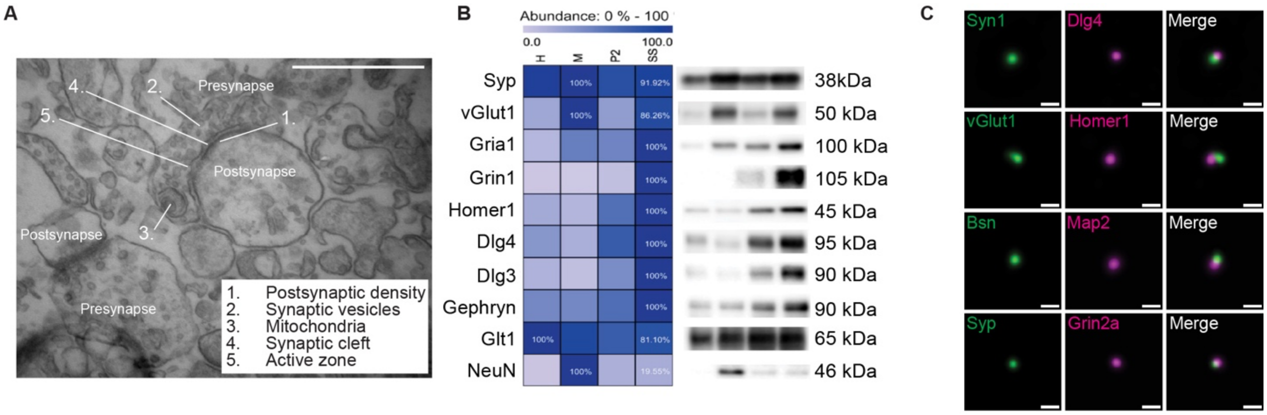

Fig. S2. Analysis of biochemically isolated synaptosomes.

(A) Representative image of isolated synaptosomes acquired using electron microscopy at 8000 x magnification. Ultrastructural features such as a postsynaptic density (1) synaptic vesicles (2), mitochondrion (3), synaptic cleft (4) and presynaptic active zone (5), are labelled. Scale bar is 1μm. **(B)** Protein abundance distribution across subcellular fractions: immunoblot analysis demonstrating a relatively higher intensity of synaptic proteins (presynaptic: vGlut1, Syp, postsynaptic: Gria1, Grin1, Homer1) and a lower intensity of non-synaptic proteins (NeuN) in purified synaptosomes (SS) compared to other subcellular fractions- homogenate (H), microsomes M), and crude synaptosomes (P2), (*n* = 2). **(C)** Representative images of immunolabelled synaptosomes spun onto BSA-coated coverslips demonstrates apposition of a range of pre- and postsynaptic proteins. Presynaptic proteins (green): Vglut1, Bsn, Syn1, and Syp and postsynaptic proteins (magenta): Homer1, Dlg4, Map2, Grin2a). Images acquired using Re-scan Confocal Microscopy. Immunohistochemical stains imaged with excitation wavelengths of 561 nm (emission wavelength: 595) and 640 nm (emission wavelength: 700), respectively. Scale bar: 1μm.

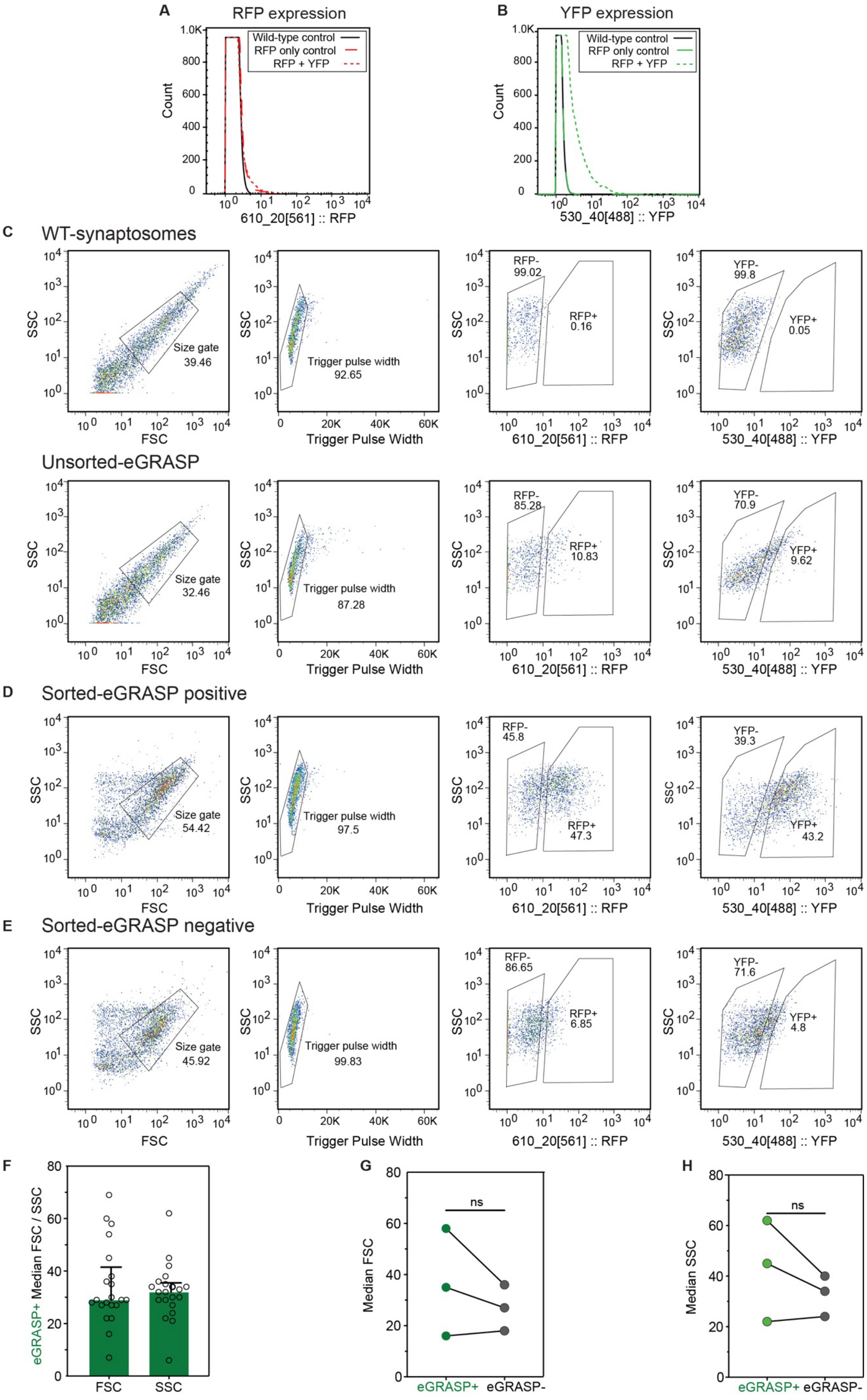
A
RFP expression
Wild-type control
RFP only control
RFP + YFP
Count
610_20[561] :: RFP
B
YFP expression
Wild-type control
RFP only control
RFP + YFP
Count
530_40[488] :: YFP
C
WT-synaptosomes
Size gate
39.46
Trigger pulse width
92.65
RFP-
99.02
RFP+
0.16
YFP-
99.8
YFP+
0.05
Unsorted-eGRASP
Size gate
32.46
Trigger pulse width
87.28
RFP-
85.28
RFP+
10.83
YFP-
70.9
YFP+
9.62
D
Sorted-eGRASP positive
Size gate
54.42
Trigger pulse width
97.5
RFP-
45.8
RFP+
47.3
YFP-
39.3
YFP+
43.2
E
Sorted-eGRASP negative
Size gate
45.92
Trigger pulse width
99.83
RFP-
86.65
RFP+
6.85
YFP-
71.6
YFP+
4.8
SSC
FSC
Trigger Pulse Width
F
eGRASP+ Median FSC / SSC
FSC
SSC
G
ns
Median FSC
eGRASP+
eGRASP-
H
ns
Median SSC
eGRASP+
eGRASP-

Fig. S3. Synaptosome sorting of eGRASP+ CA1 engram cell synapses.

(**A,B**) Histogram plot showing the expression of (A) RFP and (B) YFP signal distribution in wild-type control (black), RFP-only control (solid line) with no pre-eGRASP, and RFP+YFP+ co-expressing eGRASP+ samples (dashed line). The RFP-only control was generated by injecting post-eGRASP without pre-eGRASP to test for potential RFP bleed-through into the YFP detection channel. **(A)** Both RFP-only and RFP+YFP+ samples show more RFP expression than WT controls **(B)** RFP-only samples showed no increase in YFP signal compared to wild-type controls, confirming no detectable fluorescence spillover. In contrast, RFP+YFP co-expressing samples exhibited a clear YFP signal, validating the specificity of YFP detection and the robustness of the gating strategy. **(C)** Representative synaptosome sorting pseudocolor plots were generated for an unlabelled control (top panel: WT-synaptosomes) and unsorted eGRASP synaptosomes (bottom panel). The gating hierarchy described in Fig. 2B of the main text was followed, establishing a size gate using relative size standards (0.4 -1.35 µm) based on light scattering FSC and SSC properties. Synaptosomes exhibiting a narrow pulse width were further enriched using a trigger pulse width gate-based trigger pulse width and SSC parameters. eGRASP+ samples consisted of RFP+ events that were further sorted based on their co-expression with YFP+, while RFP–YFP– synaptosome events constituted the eGRASP- sample. Sorting was done with a 100 µm nozzle at 15 psi, piezo amplitude of 6-10 V and frequency of 25-27 KHz/sec with a 1-drop purity phase mask. **(D), (E)** Representative pseudocolor plots of sorted and reanalyzed (D) eGRASP+ and (E) eGRASP- populations. **(F)** Median FSC and SSC values from eGRASP$^+$ synaptosomes across biological replicates (n = 21). Each point represents values from an independent sort; bars show mean ± SEM. **(G–H)** Paired comparisons of FSC/SSC between sorted eGRASP$^+$ and eGRASP$^-$ synaptosomes. No significant differences were observed in (G) FSC or (H) SSC (Wilcoxon signed-rank test; n = 3 paired sorts; p = 0.5 for both).

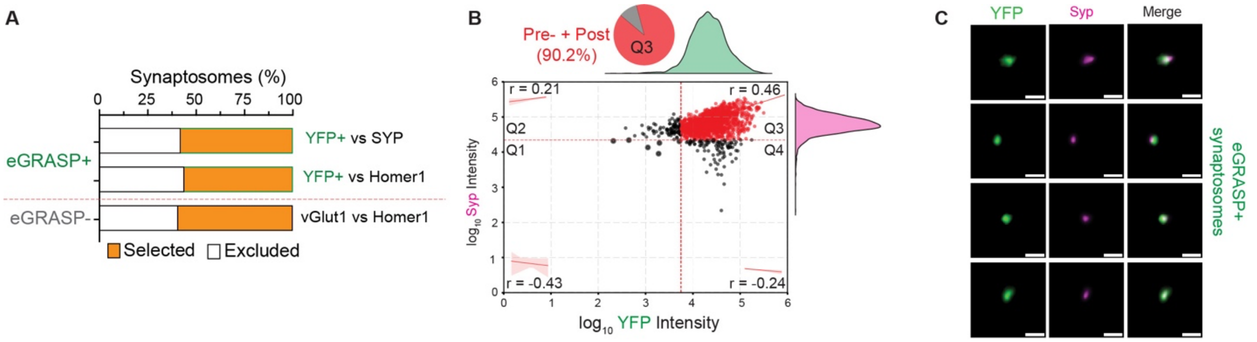

Fig. S4. Analysis of sorted eGRASP+ and eGRASP- synaptosomes.

(A) Bar graph representing the proportion of synaptosomes selected (orange) and excluded (white) for synaptosome colocalization analysis for eGRASP+ and eGRASP- samples (n=10: CFC=5, CE=5)**. (B)** Scatter plot of spearman correlation between YFP intensity (presynaptic, x-axis) and Synaptophysin (Syp) intensity (presynaptic, y-axis). Quadrant 3 (Q3), representing colocalized synaptosomes, exhibits the highest correlation coefficient (r = 0.46). The pie chart indicates that 90.2% of synaptosomes in Q3 contain both pre- and postsynaptic components. Marginal histograms display intensity distributions for each marker. (n =10: CFC = 5, CE = 5)**. (C)** Representative images of sorted eGRASP+ synaptosomes (YFP: green) immunolabelled with presynaptic protein Syp (magenta). Merged images show colocalization of presynaptic and eGRASP components. Images were acquired using Re-scan Confocal Microscopy. Scale bars: 1 µm.

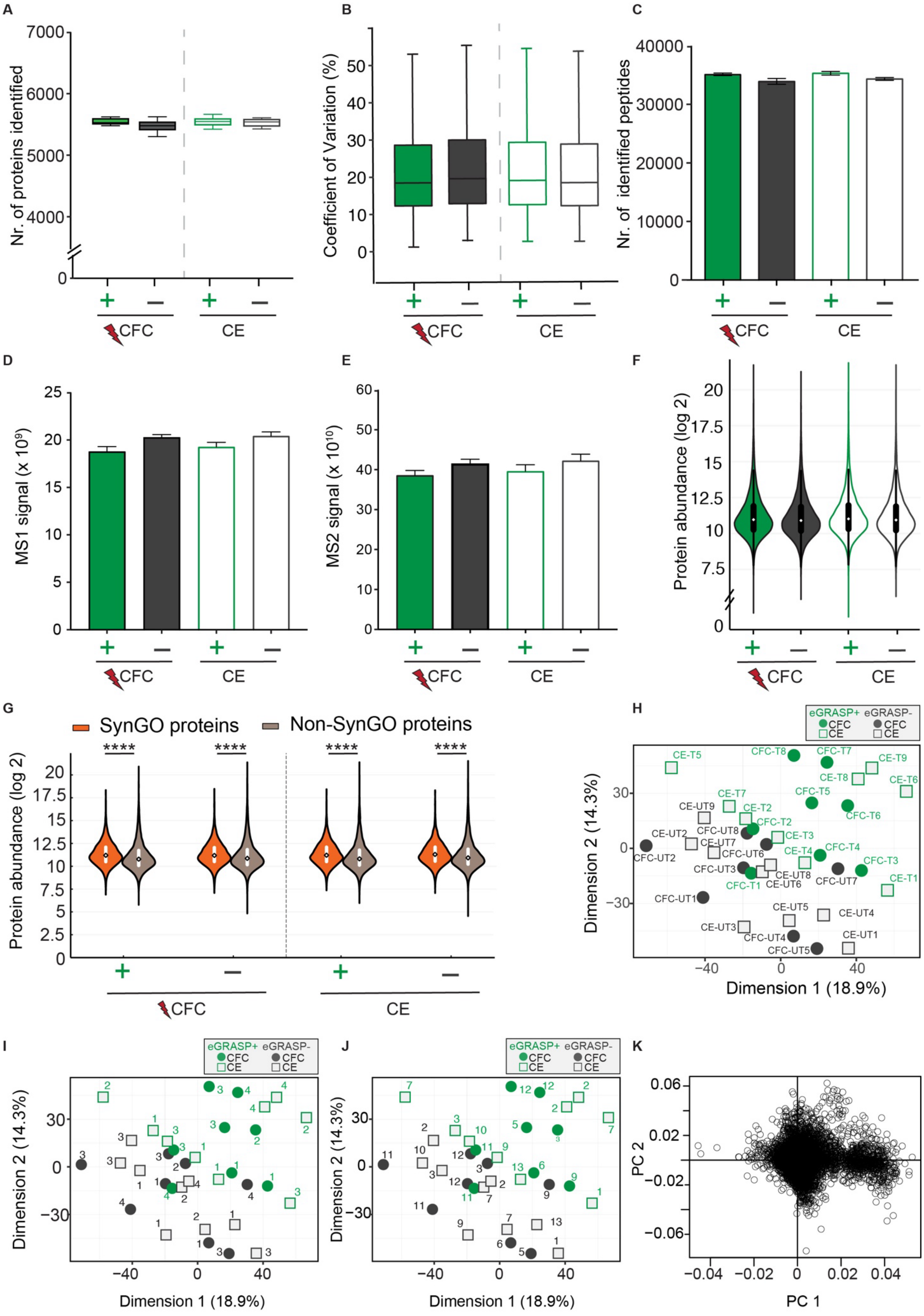

A
Nr. of proteins identified
7000
6000
5000
4000
0
+
−
+
−
CFC
CE
B
Coefficient of Variation (%)
50
40
30
20
10
0
CFC
CE
C
Nr. of identified peptides
40000
30000
20000
10000
0
CFC
CE
D
MS1 signal (x 10^9)
25
20
15
10
5
0
CFC
CE
E
MS2 signal (x 10^10)
60
50
40
30
20
10
0
CFC
CE
F
Protein abundance (log 2)
20
17.5
15
12.5
10
7.5
0
CFC
CE
G
SynGO proteins
Non-SynGO proteins
****
Protein abundance (log 2)
20
17.5
15
12.5
10
7.5
5
2.5
0
CFC
CE
H
eGRASP+ eGRASP-
CFC CFC
CE CE
Dimension 2 (14.3%)
Dimension 1 (18.9%)
−40
0
40
30
0
−30
CE-T5
CFC-T8
CFC-T7
CE-T9
CE-T8
CE-T6
CFC-T5
CE-T7
CE-T2
CFC-T6
CE-UT9
CFC-T2
CE-UT2
CFC-UT8
CE-T3
CE-UT7
CE-T4
CFC-T4
CFC-UT2
CFC-UT6
CFC-T3
CFC-UT3
CE-UT8
CE-T1
CFC-T1
CE-UT6
CFC-UT7
CFC-UT1
CE-UT5
CE-UT4
CE-UT3
CFC-UT4
CE-UT1
CFC-UT5
I
eGRASP+ eGRASP-
CFC CFC
CE CE
Dimension 2 (14.3%)
Dimension 1 (18.9%)
J
eGRASP+ eGRASP-
CFC CFC
CE CE
Dimension 2 (14.3%)
Dimension 1 (18.9%)
K
PC 2
PC 1
0.06
0.02
−0.02
−0.06
−0.04
−0.02
0
0.02
0.04

Fig. S5. Proteome quality assessment, synaptic enrichment, and PCA annotations of CFC and CE eGRASP+ and eGRASP- synaptosomes.

(A) Number of proteins identified in eGRASP+ and eGRASP- samples across CFC and CE. Box plot: mean, minimum, and maximum**. (B)** Protein coefficient of variation within replicates of eGRASP- (grey) and eGRASP+ (green) samples. CFC and CE conditions. Boxplot: median, 25th, and 75th percentiles. **(C)** Total number of identified peptides across CFC and CE eGRASP+ and eGRASP-synaptosomes. Bars represent mean ± SEM. **(D–E)** DIA-NN–reported total MS1 **(D)** and MS2 **(E)** signal quantities per sample acquired in DIA-PASEF mode. Bars represent mean ± SEM. **(F)** Violin plots showing the distribution of log2-transformed protein abundances in eGRASP+ (green) and eGRASP- (grey) synaptosomes for CFC and CE samples. The white dot represents the median, and the thick black bar within each plot represents the interquartile range (IQR). CFC: n = 8, CE: n = 9, 1 sample = 1 mouse. **(G)** Violin plots show log2 protein abundances for SynGO (orange) and non-SynGO (brown) proteins in CFC and CE eGRASP+ and eGRASP- samples. SynGO proteins were significantly more abundant within each group ($p < 0.0001$, Mann–Whitney U test). White dot: median; IQR indicated by thick bar. All pairwise SynGO vs SynGO comparisons were non-significant (Kruskal Wallis, post-hoc Dunn's multiple comparison test, $p > 0.05$). **(H–J)** PCA plot with annotations for subject identity and batch parameters. **(H)** Sample identifiers: CFC-T1-8 (CFC eGRASP+), CFC-UT1-8 (CFC eGRASP-), CE-T1-9 (CE eGRASP+), CE-UT1-9 (CE eGRASP-). **(I)** Samples annotated by behaviour batch (i.e. date of conditioning). **(J)** Samples annotated by synapse sorting batch (i.e. date of sorting). eGRASP+ and eGRASP- samples are shown in green and grey. CFC (circles, n = 8) and CE (squares, n = 9). **(K)** PCA loadings plot (PC1 vs PC2), where each point represents an individual peptide's loading vector, illustrating its contribution to sample separation and variance observed in (H–J).

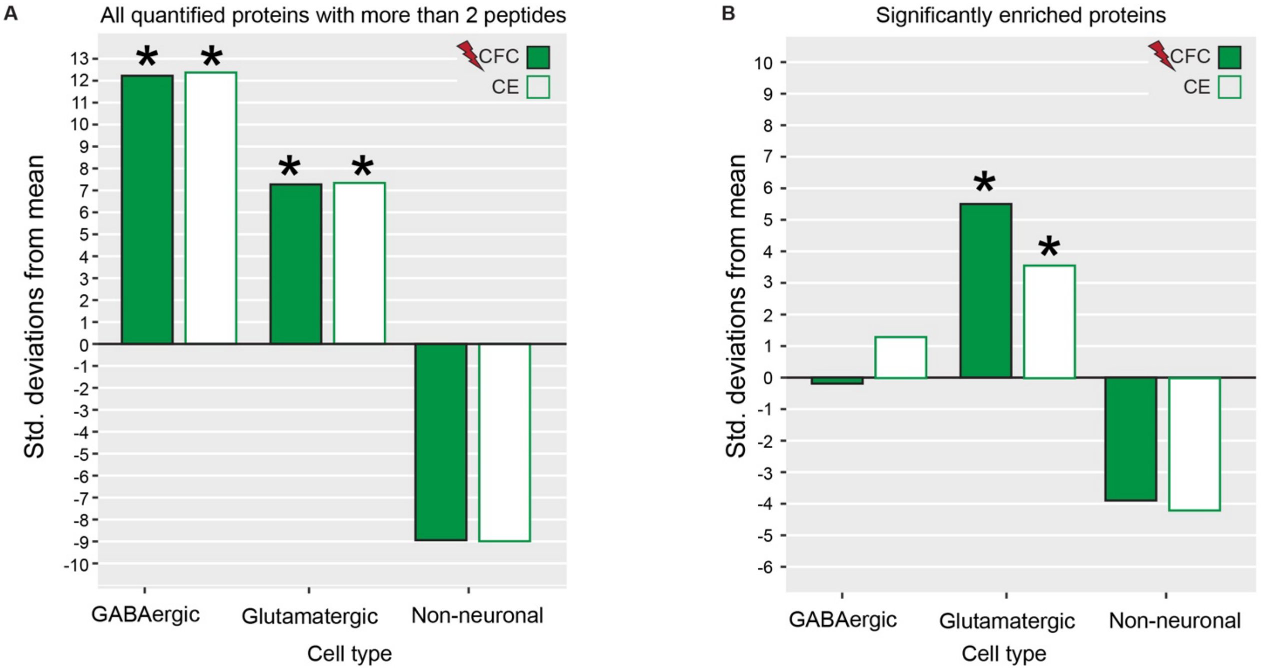

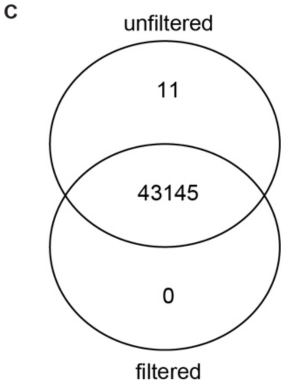

Sequence_match	Protein
NSTPVTSAFPTAK	NLGN1
QDDPKQQPSPFSVDQR	NLGN1
QQPSPFSVDQR	NLGN1
TTTNDLTHAPEEEIMSLQMK	NLGN1
DEGSYHVDESR	NRXN1
DEGSYHVDESRNYISNSAQSNGAVVK	NRXN1
EPYPGSAEVIR	NRXN1
NRDEGSYHVDESR	NRXN1
VGGREPYPGSAEVIR	NRXN1
NYISNSAQSNGAVVK	NRXN1
NYISNSAQSNGAVVKEK	NRXN1

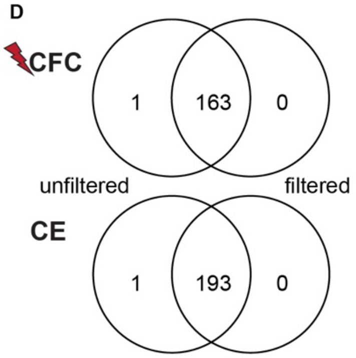

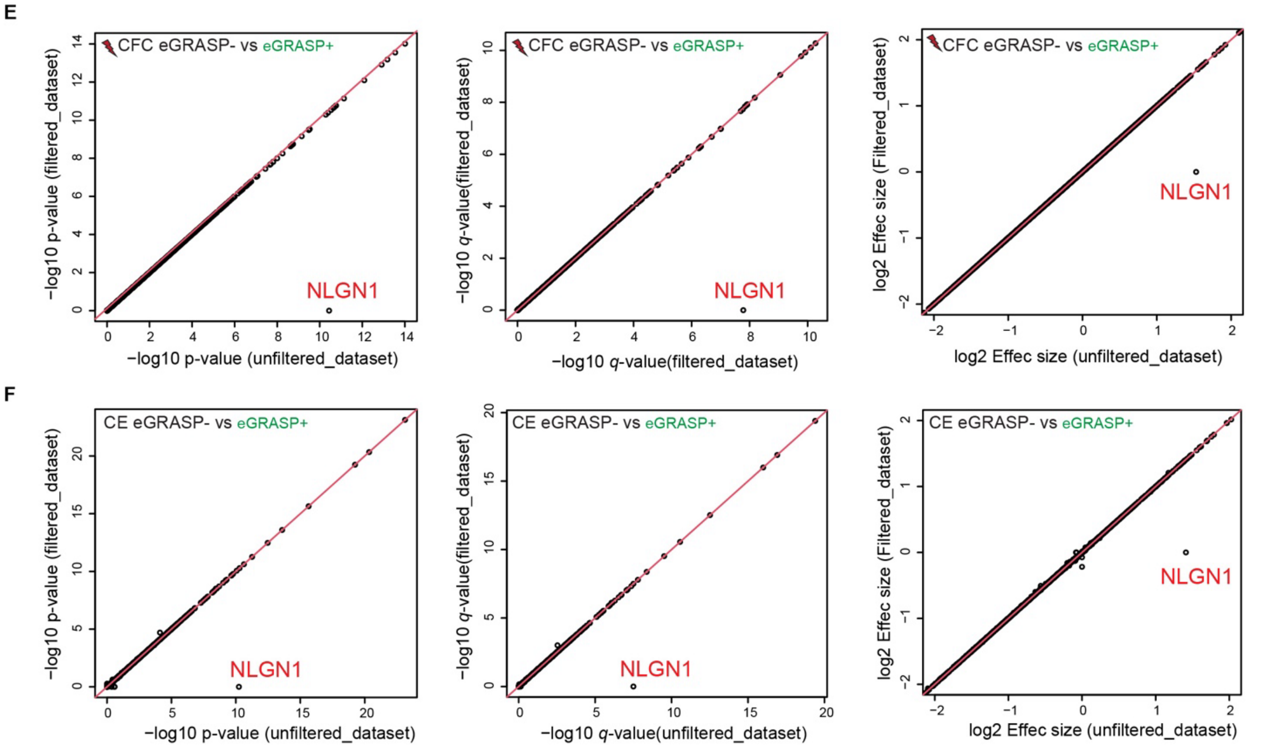

Fig. S6. Cell type enrichment analysis and evaluation of construct-derived peptide effects on quantitative robustness.

(A-B) Cell-type enrichment analysis (EWCE) (*28*) of (A) all proteins quantified with 2 or more peptides after CFC or CE in eGRASP+ and eGRASP- synaptosomes and of (B) proteins significantly enriched in each experimental group. Bootstrapping analysis shows significantly higher expression of all quantified proteins in GABAergic and glutamatergic neuronal cell types (Benjamini-Hochberg corrected *p*-values < 0.001), while significantly enriched proteins are associated with glutamatergic neurons (Benjamini-Hochberg corrected *p*-values < 0.01). Bootstrapping performed with 10,000 repetitions and mouse hippocampal single-nucleus RNA-seq data. **(C)** Left: Eleven construct-derived/shared peptides detected in the unfiltered dataset were removed from downstream quantification. Right: Table lists peptide sequences and corresponding protein assignments. **(D)** Overlap of significantly regulated proteins in the unfiltered and filtered dataset. **(E–F)** Scatter comparisons of unfiltered vs filtered results for *p*-values, *q*-values, and log2 effect sizes demonstrate concordance for: (E) CFC eGRASP- vs eGRASP+ and (F) CE eGRASP- vs eGRASP+. Data points cluster tightly along the unity line, indicating robust preservation of quantitative outcomes. Nlgn1 is highlighted in red as the only protein impacted by construct-derived peptides, with a loss of significance in the filtered analysis.

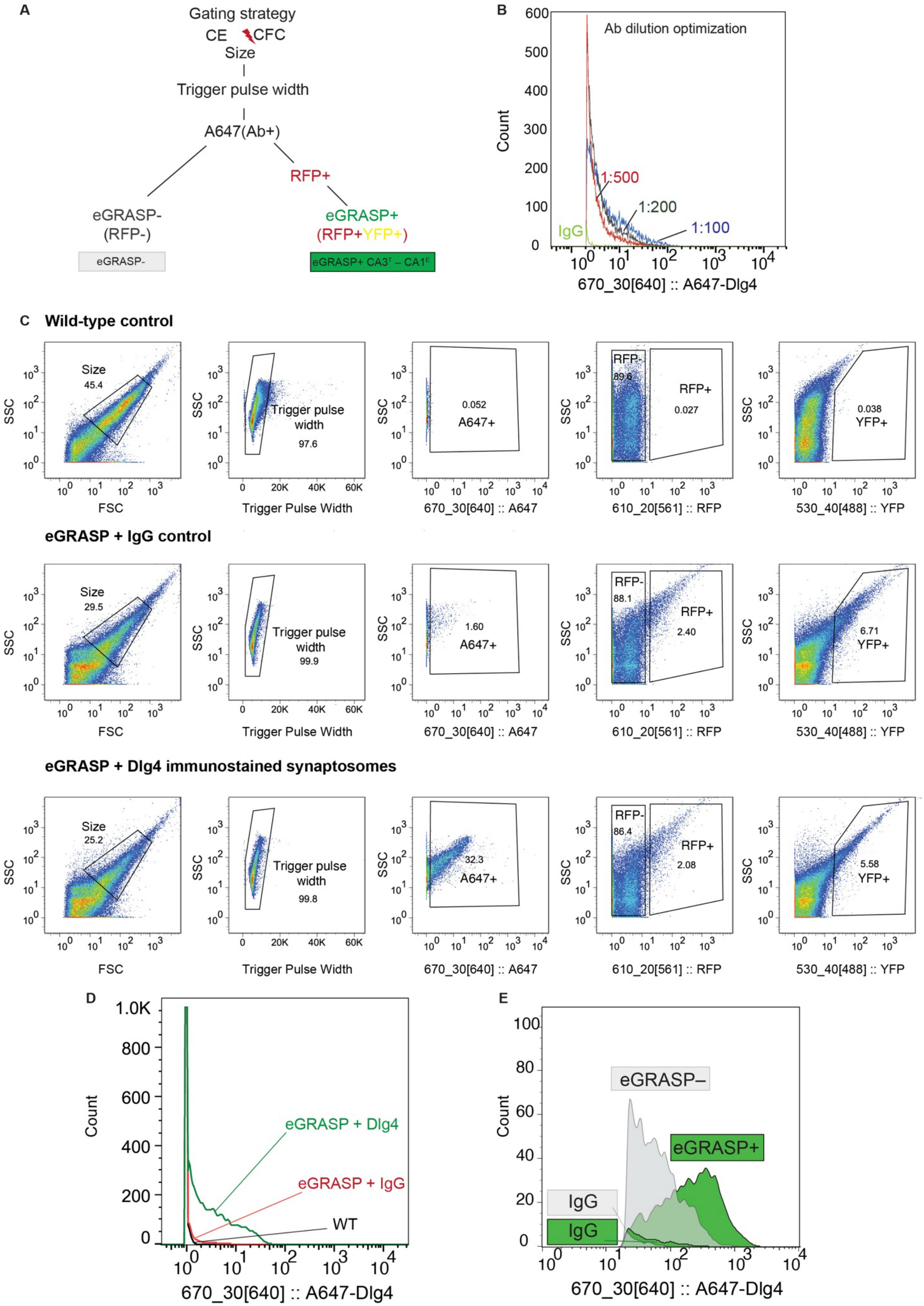
A
Gating strategy
CE
CFC
Size
Trigger pulse width
A647(Ab+)
RFP+
eGRASP-
(RFP-)
eGRASP+
(RFP+YFP+)
eGRASP-
eGRASP+ CA3T – CA1E
B
Ab dilution optimization
Count
IgG
1:500
1:200
1:100
670_30[640] :: A647-Dlg4
C
Wild-type control
Size
45.4
Trigger pulse width
97.6
0.052
A647+
RFP-
89.6
RFP+
0.027
0.038
YFP+
SSC
FSC
Trigger Pulse Width
670_30[640] :: A647
610_20[561] :: RFP
530_40[488] :: YFP
eGRASP + IgG control
Size
29.5
Trigger pulse width
99.9
1.60
A647+
RFP-
88.1
RFP+
2.40
6.71
YFP+
eGRASP + Dlg4 immunostained synaptosomes
Size
25.2
Trigger pulse width
99.8
32.3
A647+
RFP-
86.4
RFP+
2.08
5.58
YFP+
D
Count
eGRASP + Dlg4
eGRASP + IgG
WT
670_30[640] :: A647-Dlg4
E
Count
eGRASP–
eGRASP+
IgG
IgG
670_30[640] :: A647-Dlg4

Fig. S7. Immunofluorescence flow cytometry analysis for protein expression profiling.

(A) Gating hierarchy for analysis of immunolabel intensity: Immunolabelled events (A647-Ab+) with the size and trigger pulse width gate were gated based on RFP and subsequently on YFP expression. eGRASP+ events were then compared with their unlabelled (eGRASP-) counterparts independently. **(B)** Example of optimisation of antibody dilution for flow cytometry analysis (Gria2): Dilution optimisation for accurate immunodetection on the flow cytometer. The highest immunoreactivity of Gria2 in wild type synaptosomes is observed with a 1:100 (blue) concentration. Non-specific binding was measured using an IgG isotype control without secondary antibody (green). Other dilutions tested were 1:500 (red) and 1:200 (grey). This was performed for all antibodies used. **(C)** Representative pseudocolor plots generated from unstained control (WT-Synaptosomes), IgG control (eGRASP sample + IgG antibody + secondary antibody A647) and eGRASP immunolabelled samples for gating and analysing eGRASP+ and eGRASP- events in CFC and CE groups. Gating hierarchy from panel (A) was followed for generating plots. Detection of RFP utilised the 561 nm, 65 mW(BP 610/20) laser, while expression for YFP was checked with the 488 nm, 200 mW (BP 530/40) and 445 nm, 80mW (BP 480/40) lasers respectively. For fluorescent immunolabels, the 640 nm laser (670/30) operated at 100 mW was utilised. **(D)** Representative histogram plot comparing the A647-antibody (Dlg4) intensity of unstained control (WT-Synaptosomes), IgG control (eGRASP sample + IgG antibody + secondary antibody A647) and RFP+YFP+ (eGRASP) immunolabelled samples. While WT-synaptosomes sets the threshold, IgG control measures the background fluorescence for immunolabel expression analysis for eGRASP immunolabelled samples. (**E**) Representative histogram plot comparing the A647-antibody (Dlg4) intensity of eGRASP+ (green) and eGRASP- (grey) synaptosomes, with the IgG control expression used to measure background fluorescence. Corrected median intensity values were calculated using the IgG control.

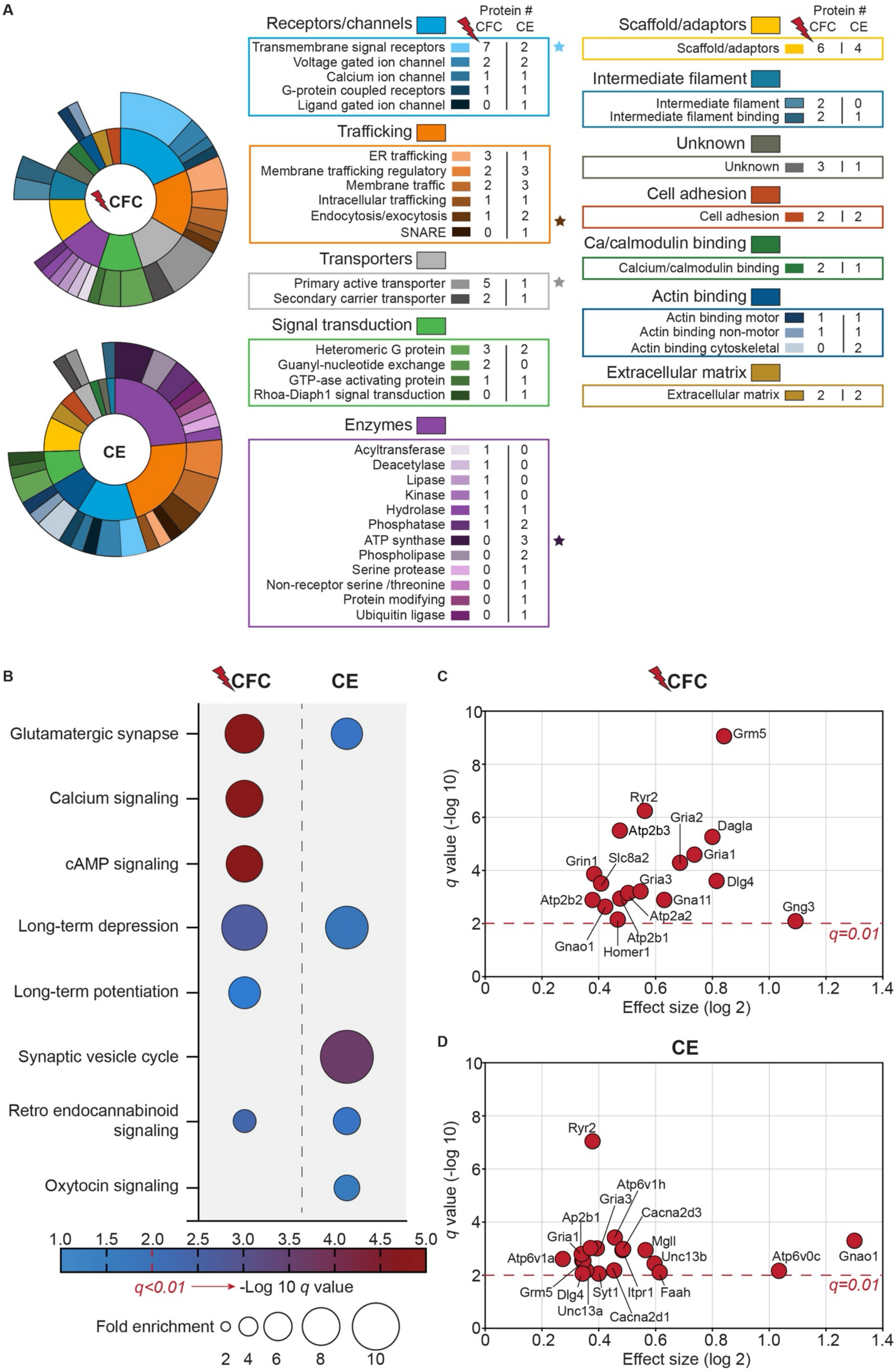
A
CFC
CE
Protein #
CFC CE
Receptors/channels
Transmembrane signal receptors 7 2
Voltage gated ion channel 2 2
Calcium ion channel 1 1
G-protein coupled receptors 1 1
Ligand gated ion channel 0 1
Trafficking
ER trafficking 3 1
Membrane trafficking regulatory 2 3
Membrane traffic 2 3
Intracellular trafficking 1 1
Endocytosis/exocytosis 1 2
SNARE 0 1
Transporters
Primary active transporter 5 1
Secondary carrier transporter 2 1
Signal transduction
Heteromeric G protein 3 2
Guanyl-nucleotide exchange 2 0
GTP-ase activating protein 1 1
Rhoa-Diaph1 signal transduction 0 1
Enzymes
Acyltransferase 1 0
Deacetylase 1 0
Lipase 1 0
Kinase 1 0
Hydrolase 1 1
Phosphatase 1 2
ATP synthase 0 3
Phospholipase 0 2
Serine protease 0 1
Non-receptor serine /threonine 0 1
Protein modifying 0 1
Ubiquitin ligase 0 1
Protein #
CFC CE
Scaffold/adaptors
Scaffold/adaptors 6 4
Intermediate filament
Intermediate filament 2 0
Intermediate filament binding 2 1
Unknown
Unknown 3 1
Cell adhesion
Cell adhesion 2 2
Ca/calmodulin binding
Calcium/calmodulin binding 2 1
Actin binding
Actin binding motor 1 1
Actin binding non-motor 1 1
Actin binding cytoskeletal 0 2
Extracellular matrix
Extracellular matrix 2 2
B
CFC
CE
Glutamatergic synapse
Calcium signaling
cAMP signaling
Long-term depression
Long-term potentiation
Synaptic vesicle cycle
Retro endocannabinoid signaling
Oxytocin signaling
1.0 1.5 2.0 2.5 3.0 3.5 4.0 4.5 5.0
q<0.01 -Log 10 q value
Fold enrichment
2 4 6 8 10
C
CFC
q value (-log 10)
Effect size (log 2)
Grm5
Ryr2
Gria2
Dagla
Atp2b3
Gria1
Grin1
Slc8a2
Dlg4
Gria3
Atp2b2
Gna11
Atp2a2
Gng3
Atp2b1
Gnao1
Homer1
q=0.01
D
CE
q value (-log 10)
Effect size (log 2)
Ryr2
Atp6v1h
Gria3
Cacna2d3
Ap2b1
Gria1
Mgll
Unc13b
Atp6v1a
Atp6v0c
Gnao1
Grm5
Dlg4
Syt1
Itpr1
Faah
Unc13a
Cacna2d1
q=0.01

Fig. S8. Protein classes and KEGG pathways related to the proteome enriched in CA3- CA1 engram cell synapses.

(A) Proteins enriched after CFC or CE were assigned to categories based on protein class and function (also see Supplementary data 6). Plotted sunburst displays the distribution of functional categories (inner circle) and subcategories (outer circle) enriched after CFC or CE, with number of proteins assigned to each displayed on the right. Stars indicate subcategory differences between CFC and CE that contribute to enriched KEGG pathways (B). **(B)** A selection of highlighted KEGG pathways enriched after CFC or CE (also see Supplementary data 7 for all pathways). Size and colour of circles denote fold enrichment and *q-value* (-log10 FDR) respectively of proteins associated with the term. **(C)** CFC enriched proteins associated with the KEGG pathways displayed in (B), with their -log10 *q-value* and effect size derived from differential expression analysis. **(D)** CE enriched proteins associated with the KEGG pathways displayed in (B), with their -log10 *q-value* and effect size derived from differential expression analysis. Dotted line: significance drawn at q = 0.01.

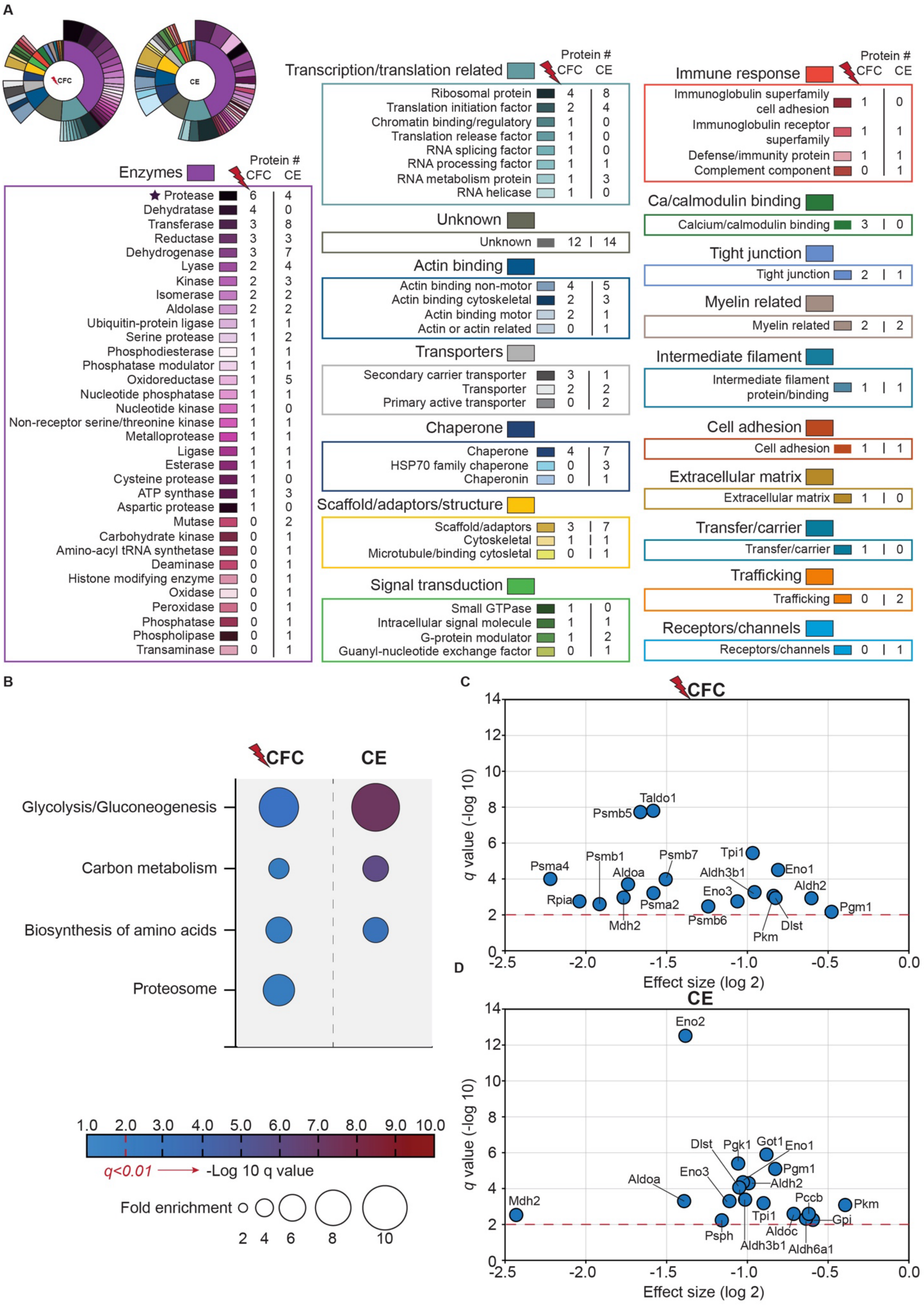
A
CFC
CE
Enzymes
Protein #
CFC CE
Protease 6 4
Dehydratase 4 0
Transferase 3 8
Reductase 3 3
Dehydrogenase 3 7
Lyase 2 4
Kinase 2 3
Isomerase 2 2
Aldolase 2 2
Ubiquitin-protein ligase 1 1
Serine protease 1 2
Phosphodiesterase 1 1
Phosphatase modulator 1 1
Oxidoreductase 1 5
Nucleotide phosphatase 1 1
Nucleotide kinase 1 0
Non-receptor serine/threonine kinase 1 1
Metalloprotease 1 1
Ligase 1 1
Esterase 1 1
Cysteine protease 1 0
ATP synthase 1 3
Aspartic protease 1 0
Mutase 0 2
Carbohydrate kinase 0 1
Amino-acyl tRNA synthetase 0 1
Deaminase 0 1
Histone modifying enzyme 0 1
Oxidase 0 1
Peroxidase 0 1
Phosphatase 0 1
Phospholipase 0 1
Transaminase 0 1
Transcription/translation related
Protein #
CFC CE
Ribosomal protein 4 8
Translation initiation factor 2 4
Chromatin binding/regulatory 1 0
Translation release factor 1 0
RNA splicing factor 1 0
RNA processing factor 1 1
RNA metabolism protein 1 3
RNA helicase 1 0
Unknown
Unknown 12 14
Actin binding
Actin binding non-motor 4 5
Actin binding cytoskeletal 2 3
Actin binding motor 2 1
Actin or actin related 0 1
Transporters
Secondary carrier transporter 3 1
Transporter 2 2
Primary active transporter 0 2
Chaperone
Chaperone 4 7
HSP70 family chaperone 0 3
Chaperonin 0 1
Scaffold/adaptors/structure
Scaffold/adaptors 3 7
Cytoskeletal 1 1
Microtubule/binding cytosletal 0 1
Signal transduction
Small GTPase 1 0
Intracellular signal molecule 1 1
G-protein modulator 1 2
Guanyl-nucleotide exchange factor 0 1
Immune response
Protein #
CFC CE
Immunoglobulin superfamily cell adhesion 1 0
Immunoglobulin receptor superfamily 1 1
Defense/immunity protein 1 1
Complement component 0 1
Ca/calmodulin binding
Calcium/calmodulin binding 3 0
Tight junction
Tight junction 2 1
Myelin related
Myelin related 2 2
Intermediate filament
Intermediate filament protein/binding 1 1
Cell adhesion
Cell adhesion 1 1
Extracellular matrix
Extracellular matrix 1 0
Transfer/carrier
Transfer/carrier 1 0
Trafficking
Trafficking 0 2
Receptors/channels
Receptors/channels 0 1
B
CFC
CE
Glycolysis/Gluconeogenesis
Carbon metabolism
Biosynthesis of amino acids
Proteosome
1.0 2.0 3.0 4.0 5.0 6.0 7.0 8.0 9.0 10.0
q<0.01
-Log 10 q value
Fold enrichment
2 4 6 8 10
C
CFC
q value (-log 10)
Effect size (log 2)
Taldo1
Psmb5
Psma4
Psmb1
Aldoa
Psmb7
Tpi1
Aldh3b1
Eno1
Aldh2
Eno3
Rpia
Psma2
Mdh2
Psmb6
Pkm
Dlst
Pgm1
D
CE
q value (-log 10)
Effect size (log 2)
Eno2
Dlst
Pgk1
Got1
Eno1
Pgm1
Eno3
Aldh2
Aldoa
Mdh2
Pccb
Pkm
Tpi1
Gpi
Psph
Aldoc
Aldh3b1
Aldh6a1

Fig. S9. Protein classes and KEGG pathways related to the proteome depleted in CA3- CA1 engram cell synapses.

(A) Proteins depleted after CFC or CE were assigned to categories based on protein class and function**.** Plotted sunburst displays the distribution of 18 functional categories (inner circle) and 62 subcategories (outer circle) depleted after CFC or CE, with number of proteins assigned to each displayed on the right**.** Stars indicate subcategory differences between CFC and CE that contribute to enriched KEGG pathways (B). **(B)** A selection of highlighted KEGG pathways enriched after CFC or CE (also see Supplementary data 7 for all pathways), driven by depleted proteins. Size and colour of circles denote fold enrichment and *q-value* (-log10 FDR) respectively of proteins associated with the term. **(C)** CFC depleted proteins (blue) associated with the KEGG pathways displayed in (B), with their -log10 *q*-value and effect size derived from differential expression analysis. **(D)** CE depleted proteins (blue) associated with the KEGG pathways displayed in (B), with their -log10 *q*-value and effect size derived from differential expression analysis. Dotted line: significance drawn at $q = 0.01$.

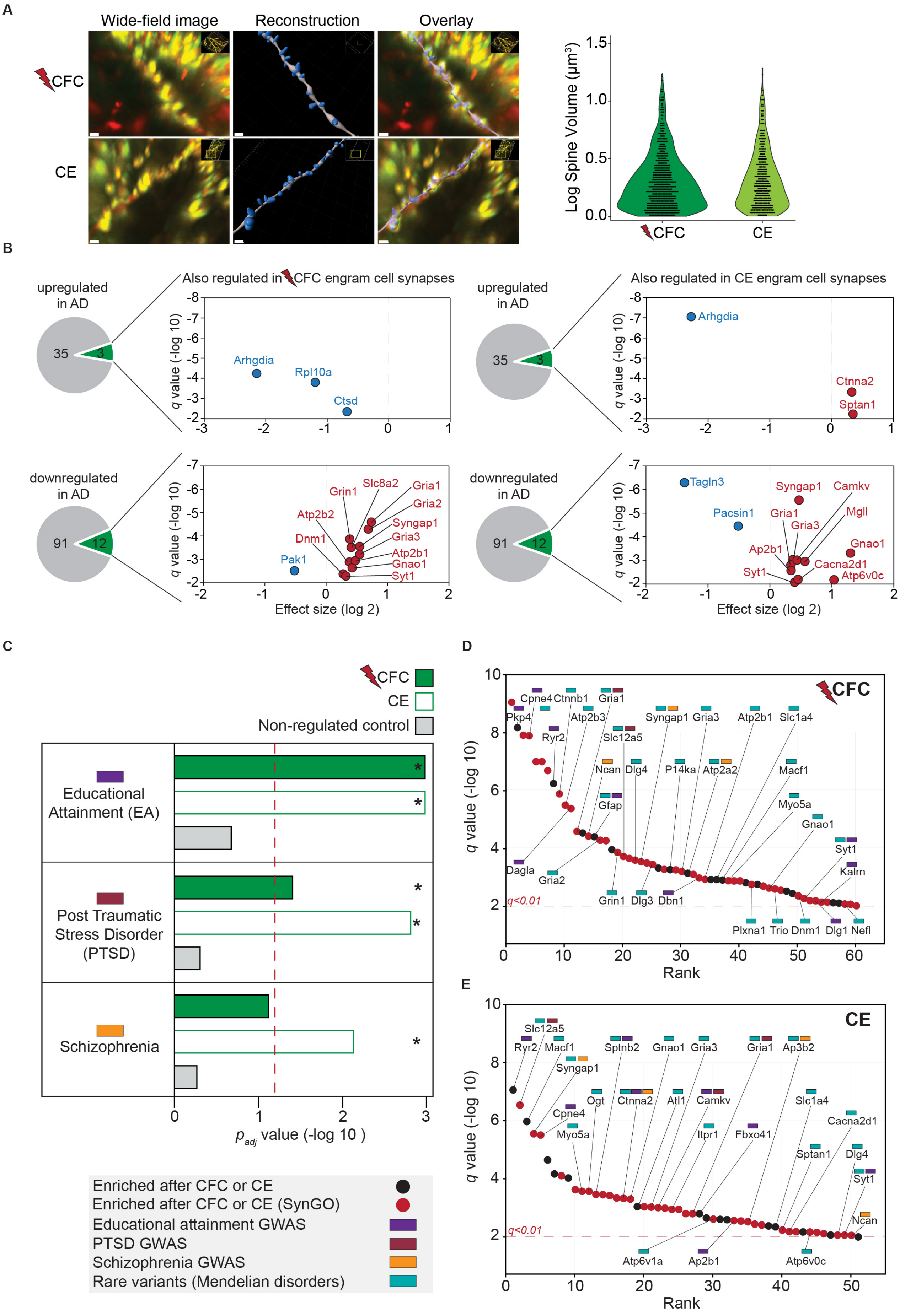
A
Wide-field image
Reconstruction
Overlay
CFC
CE
Log Spine Volume (µm³)
1.5
1.0
0.5
0.0
CFC
CE
B
upregulated in AD
35
3
Also regulated in CFC engram cell synapses
q value (-log 10)
Arhgdia
Rpl10a
Ctsd
downregulated in AD
91
12
Grin1
Slc8a2
Gria1
Gria2
Atp2b2
Syngap1
Gria3
Dnm1
Atp2b1
Gnao1
Pak1
Syt1
Effect size (log 2)
Also regulated in CE engram cell synapses
Arhgdia
Ctnna2
Sptan1
Tagln3
Syngap1
Camkv
Pacsin1
Gria1
Mgll
Gria3
Ap2b1
Gnao1
Syt1
Cacna2d1
Atp6v0c
C
CFC
CE
Non-regulated control
Educational Attainment (EA)
Post Traumatic Stress Disorder (PTSD)
Schizophrenia
p_{adj} value (-log 10)
Enriched after CFC or CE
Enriched after CFC or CE (SynGO)
Educational attainment GWAS
PTSD GWAS
Schizophrenia GWAS
Rare variants (Mendelian disorders)
D
CFC
q value (-log 10)
Pkp4
Cpne4
Ctnnb1
Gria1
Atp2b3
Ryr2
Syngap1
Gria3
Atp2b1
Slc1a4
Slc12a5
Ncan
Dlg4
P14ka
Atp2a2
Macf1
Gfap
Myo5a
Gnao1
Syt1
Dagla
Gria2
Kalrn
q<0.01
Grin1
Dlg3
Dbn1
Plxna1
Trio
Dnm1
Dlg1
Nefl
Rank
E
CE
Slc12a5
Ryr2
Macf1
Sptnb2
Gnao1
Gria3
Gria1
Ap3b2
Syngap1
Ogt
Ctnna2
Atl1
Camkv
Slc1a4
Cpne4
Myo5a
Itpr1
Fbxo41
Cacna2d1
Sptan1
Dlg4
Syt1
Ncan
q<0.01
Atp6v1a
Ap2b1
Atp6v0c
Rank

Fig. S10. CA1 engram cell proteome is enriched with proteins involved in cognitive function.

(A) Left: Representative images demonstrating deconvolved fluorescent signal captured with a widefield microscope (RFP in red, YFP in green, overlap RFP and YFP in yellow), reconstructed dendrite (in grey) and spines (in blue) for analysis after CFC (upper panel) and CE (lower panel). Scale bar: 2 µm. Right: Spine volume of $CA3^{T}$-$CA1^{E}$ eGRASP labelled postsynaptic spines in the dorsal CA1 did not differ between the CFC (n = 975) and CE (n = 605) condition. Each data point represents a spine. Wilcoxon rank sum test: $W = 298758$, $p = 0.665$. **(B)** Overlap between SynGO annotated proteins dysregulated in human postmortem CA1/subiculum tissue of AD across all Braak stages (0-VI) (*32*) and proteins regulated in CFC (left) and CE (right) engram cell synapses ranked according to their significance. Upper panel: Of 38 SynGO proteins upregulated in AD, 3 were also regulated after CFC or CE. Lower panel: Of 103 SynGO proteins downregulated in AD, 12 were also regulated after CFC or CE. **(C)** GWAS traits significantly enriched in CFC and CE enrichment datasets (also see Supplementary data 12) (Fisher exact test with Benjamini-Hochberg FDR correction. EA $p_{adj:}$ CFC: 0.0010, CE: 0.0010, PTSD $p_{adj:}$ CFC: 0.0385, CE: 0.0015, Schizophrenia $p_{adj:}$ CE: 0.0072). The 100 least significantly regulated proteins common to both experimental groups were used as a non-regulated control set. Red dashed line indicates significance threshold set at $-\log10\ p_{adj} > 1.3$ ($*p_{adj} < 0.05$). **(D, E)** Proteins enriched after CFC (B) and CE (C) ranked according to their significance, with those (i) overlapping with significantly enriched GWAS traits (also see Supplementary data 12) (EA: purple, PTSD: maroon, Schizophrenia: orange) and (ii) containing rare variants linked to Mendelian disorders (cyan). SynGO proteins are highlighted in red.

Supplementary Tables

Table S1. Materials used for the study, with their corresponding sources, catalog numbers, RRIDs, and relevant identifiers.

REAGENT or RESOURCE	SOURCE	IDENTIFIER
Antibodies		
anti-RFP	Tebubio BV	Cat# 600-401-379,RRID:AB_2209751
anti-c-Fos	Synaptic Systems	Cat# 226 017, RRID:AB_2864765
anti-Syp	Synaptic Systems	Cat# 101 004, RRID:AB_1210382
anti-Grin2a	Abcam	Cat# ab124913, RRID:AB_10975154
anti-Syn1	Chemicon	Cat# AB1543P, RRID:AB_90757
anti-Dlg4	Neuromab	Cat# 75-028, RRID:AB_2292909
anti-Dlg4	Invitrogen	Cat# MA1-046, RRID:AB_2092361
anti-vGlut1	Synaptic Systems	Cat# 135 302, RRID:AB_887877
anti-vGlut1	Synaptic Systems	Cat# 135 011BT, RRID:AB_2884913
anti-Homer1	Synaptic Systems	Cat# 160 004, RRID:AB_10549720
anti-Bsn	Enzo Life Sciences	Cat# SAP7F407, RRID:AB_2313990
anti-Map2	Sigma-Aldrich	Cat# AB5543, RRID:AB_571049
anti-Gria2	Abcam	Cat# ab133477, RRID:AB_2620181
anti-Dlg3	Invitrogen	Cat# PA5-29116, RRID:AB_2546592
anti-Unc13B	Synaptic Systems	Cat# 126 203, RRID:AB_2619807
anti-Grm5	Synaptic Systems	Cat# 191 508, RRID:AB_3696864
anti-Grm5	Invitrogen	Cat# MA5-24185, RRID: AB_2608804
anti-Slc12a5	Abcam	Cat# ab259969
anti-Dbn1	Proteintech	Cat# 10260-1-AP, RRID: AB_2230301
anti-Gnao1	Invitrogen	Cat# PA5-26142, RRID: AB_2543642
anti-Ap2B1	Abcam	Cat# ab220778, RRID:AB_3099441
anti-Unc13A	Synaptic Systems	Cat# 126 103, RRID:AB_887733
anti-Gria1	Abcam	Cat# ab109450, RRID:AB_10860361
anti-Grin1	Neuromab	Cat# 75-272, RRID:AB_11000180
anti-Gephryn	Synaptic Systems	Cat# 147 111, RRID:AB_887719
anti-Glt1	Invitrogen	Cat#PA5-17099, RRID:AB_10978571
anti-NeuN	Sigma-Aldrich	Cat# MAB377, RRID:AB_2298772
Rabbit IgG isotype control	Invitrogen	Cat# 08-6199, RRID:AB_2532942
Mouse IgG isotype control	Invitrogen	Cat# 10400C, RRID: AB_2532980
Anti-Guinea Pig Alexa Fluor™ 647	Invitrogen	Cat# A-21450, RRID:AB_AB_2535867
Anti-Rabbit Alexa Fluor™ 568	Invitrogen	Cat# A-11011, RRID:AB_143157
Anti-Mouse Alexa Fluor™ 568	Invitrogen	Cat# A-11004, RRID:AB_2534072
Anti-Chicken Alexa Fluor™ 647	Invitrogen	Cat# A-21449, RRID:AB_2535866
Anti-Rabbit Alexa Fluor™ 633	Invitrogen	Cat# A-21070, RRID:AB_2535731
Anti-Rabbit Alexa Fluor™ 647	Abcam	Cat# ab150079, RRID:AB_2722623

Anti-Rabbit Alexa Fluor™ 647	Invitrogen	Cat # A-21245, RRID: AB_2535813
Anti-Mouse Alexa Fluor™ 647	Invitrogen	Cat# A-21235, RRID:AB_AB_2535804
Anti-Rat Alexa Fluor™ 633	Invitrogen	Cat# A-21094, RRID:AB_2535749
Anti-Mouse IgG	Agilent Dako	Cat# P0447, RRID:AB_2617137
Anti-Rabbit IgG	Agilent Dako	Cat# P0448, RRID:AB_2617138
Anti-Guinea Pig IgG	Agilent Dako	Cat# P0141, RRID:AB_2941309
Bacterial and virus strains		
AAV-CWB-yellow pre-eGRASP(p32)	(*10*)	Addgene plasmid #111580
AAV-EWB-DIO-yellow pre-eGRASP(p32)	In-house	N/A
AAV-EWB-DIO-cyan pre-eGRASP(p32)	(*10*)	Addgene plasmid #111589
AAV-EWB-DIO-myrTagRFP-T-P2A-post-eGRASP	(*10*)	Addgene plasmid # 111581
AAV-Fos::CreERT2	(*22*)	N/A
Chemicals, peptides, and recombinant proteins		
Isofluorane	RB Pharmaceuticals	ATC-Code N01AB06
Lidocaine	Sigma-Aldrich	CAS No.: 137-58-6
Temgesic	RB Pharmaceuticals	ATC-Code N02AE01
4-hydroxytamoxifen	Hello Bio	Cat# HB6040; CAS No.: 68392-35-8
DMSO	Sigma-Aldrich	Cat# D8418; CAS No.: 67-68-5
Tween80	Sigma-Aldrich	Cat# P1754; CAS No.: 9005-65-6
Tribromoethanol	Thermo Fisher Scientific	Cat #11422988; CAS No.: 75-80-9
Active Amyl Alcohol	Sigma-Aldrich	Cat# 100975; CAS No.:71-41-0
Normal goat serum	Thermo Fisher Scientific	Cat #11530526
Bovine serum albumin	Sigma-Aldrich	Cat #A7906-500gr; CAS No.: 9048-46-8
Triton-X-100	Sigma-Aldrich	Cat #X100-500ML; CAS No.: 9002-93-1
DAPI	Thermo Fisher Scientific	Cat# D3571; CAS No.: 28718-90-3
ProLong Antifade Mountant	Thermo Fisher Scientific	Cat #P36980
Sucrose	Sigma-Aldrich	Cat #S0389-1KG; CAS No.: 57-50-1
HEPES	Sigma-Aldrich	Cat #H4034; CAS No.: 7365-45-9
EDTA-free Protease Inhibitor Cocktail	Roche	Cat #11873580001
Tween-20	Sigma-Aldrich	Cat #P2287-500ML; CAS No.: 9005-64-5
Super signal West Femto 100 ml	Thermo Fisher Scientific	Cat # 34095
Pluronic F-68 surfactant	Thermo Fisher Scientific	Cat #24040032
Pierce HeLa protein digest standard	Thermo Fisher Scientific	Cat # 88329
Trypsin/Lys-C	Promega	Quote 216006-0
Tris	Sigma-Aldrich	Cat #C4706-2G; CAS No.: 51805-45-9
Paraformaldehyde	Thermo Fisher Scientific	Cat #10342243; CAS No.:30525-89-4
Rainbow Calibration Particles	Invitrogen	Cat # A34305
SPHERO™ Flow Cytometry Particle Size Standard Kit	Spherotech	Cat# NFPPS-52-4K
Software and algorithms		
Ethovision XT	Noldus, commercial (~€4000 license)	https://www.noldus.com/ethovision-xt

Fiji imageJ	(*69),* Open source, free	https://imagej.net/software/fiji/
Video Freeze® Video Fear Conditioning Software	Med Associates Inc., commercial (~€600 license)	https://med-associates.com/product/videofreeze-video-fear-conditioning-software/
GraphPad Prism 10	GraphPad Software, commercial (~€550/ year license)	https://www.graphpad.com/
FlowJO v.10	BD Biosciences, commercial (~€350/ year license)	https://www.flowjo.com/solutions/flowjo
Huygens Professional v21.10	Scientific Volume Imaging BV, commercial (~€6200 license)	https://svi.nl/Huygens-Professional
SynaptosomesMacro v1.0	(*26),* custom macro, free	https://github.com/fabricecordelieres/IJ-Toolset_SynaptosomesMacro.git
DIA-NN 1.8.1	(*18),* Open source, free	https://github.com/vdemichev/DiaNN.git
MS-DAP 1.0.5	(*80),* Open source, free	https://github.com/ftwkoopmans/msdap.git
ShinyGO 0.8067	(*83),* Web tool, free	http://bioinformatics.sdstate.edu/go/
SynGO 1.2	(*27),* Web tool, free	https://syngoportal.org/
StringDB 12.0	(*87),* Web tool, free	https://string-db.org/
Imaris 9.8 and Imaris 10.2	Bitplane AG, commercial (~€15000 license)	https://imaris.oxinst.com
R Studio	R programming, open source	https://github.com/rstudio/rstudio.git
Image Studio™ Lite Software	LI-COR, free	https://www.licor.com/bio/image-studio/
IDLE 3.12.0	Python environment, open source	https://www.python.org
pandas 2.2.3	Python package, open source	https://pandas.pydata.org
NumPy 2.1.2	Python package, open source	https://numpy.org/
SciPy. 1.14.1	Python package, open source	https://scipy.org
statsmodels 0.14.4	Python package, open source	https://www.statsmodels.org
matplotlib 3.9.2	Python package, open source	https://matplotlib.org
seaborn 0.13.2	Python package, open source	https://seaborn.pydata.org
Cell lines and Primary cultures		
Neuro-2a	ATCC	Cat # CCL-131

Table S2.

Summary statistics

Figure	Comparison, sample size	Statistical Test	One /Two-tailed	*p-value*	*t/W* or *F* value, *Stat*
**1B**	Conditioning vs. Retrieval (*n* = 6)	Paired Wilcoxon signed-ran test	Two-tailed	*0.036*	*W = 21*
**1D**	HC (*n* = 4) vs. CFC (*n* = 5)	Wilcoxon rank sum test	Two-tailed	*0.02*	*W = 0*
**1E**	HC (*n* = 4) vs. CFC (*n* = 5)	Independent samples *t*-test	Two-tailed	*0.035*	*t(6.61) = -2.65*
**1H**	HC vs. CFC, *n*-slice = 15, *N*-dendrite = 34	hierarchical GLM		*< 0.001*	*b = 3.60, t = 7.68*
**1I**	HC v CFC, *n*-slice = 15, *n*-dendrite = 77, *N*-spine = 595	hierarchical GLM		*0.025*	*b* = -0.92, *t* = -2.25
**2D**	Unsorted (*n* = 11) vs. Sorted (*n* = 10)	Paired *t*-test	Two-tailed	*< 0.0001*	*t=30.20, df=20*
**3C**	Gria2-CFC: eGRASP- vs eGRASP, *n* = 5	Wilcoxon signed rank test	One-tailed	*0.0312*	*W=15*
	Gria2-CE:eGRASP- vs. eGRASP+, *n* = 5	Wilcoxon signed rank test	Two-tailed	*0.0625*	*W=15*
	Dlg3-CFC: eGRASP- vs. eGRASP+, *n* = 5	Wilcoxon signed rank test	One-tailed	*0.0312*	*W=15*
	Dlg3-CE:eGRASP- vs. eGRASP+, *n* = 5	Wilcoxon signed rank test	Two-tailed	*0.0625*	*W=15*
**3D**	Unc13b-CFC: eGRASP- vs. eGRASP+, *n* = 5	Wilcoxon signed rank test	Two-tailed	*0.0625*	*W=15*
	Unc13b-CE:eGRASP- vs. eGRASP+, *n* = 5	Paired *t*-test	One-tailed	*0.00349*	*t=5.101,df=4*
	Ap2b1-CFC: eGRASP- vs. eGRASP+, *n* = 5	Paired *t*-test	Two-tailed	*0.0004*	*t=10.91, df=4*
	Ap2b1-CE:eGRASP- vs. eGRASP+, *n* = 5	Paired *t*-test	One-tailed	*< 0.0001*	*t=14.14861, df=4*
**3E**	Dlg4-CFC: eGRASP- vs. eGRASP+, *n* = 5	Paired *t*-test	One-tailed	*0.0001*	*t=12.55, df=4*
	Dlg4-CE:eGRASP- vs. eGRASP+, *n* = 5	Paired *t*-test	One-tailed	*0.000303*	*t=9.810, df=4*
	Unc13a-CFC: eGRASP- vs. eGRASP+, *n* = 5	Paired *t*-test	One-tailed	*0.000164*	*t=11.48, df=4*
	Unc13a-CE:eGRASP- vs. eGRASP+, *n* = 4	Paired *t*-test	One-tailed	*0.014567*	*t=3.940, df=3*
**6C**	Dominance (CFC/CE) vs. SynGO-CC location	Chi-square test		*0.0007*	*Chi2 Stat = 16.9867, df = 3*
**6D**	Control vs. Dominant proteins distribution	Kolmogorov–Smirnov test		*0.0317*	*K-S Stat = 0.2460*
**6E**	Postsynapse	Logistic regression		*0.013*	*log (OR) = 2.5649, CI= (0.531, 4.599)*
	Presynapse	Logistic regression		*0.178*	*log (OR) = -1.0986, CI= (-2.699, 0.502)*

**7D**	Grm5: eGRASP+ T-E (*n* = 8) vs. eGRASP+ E-E (*n* = 8)	Unpaired t-test	Two-tailed	*0.00004*	*t = -5.922*
	Gria2: eGRASP+ T-E (*n* = 8) vs. eGRASP+ E-E (*n* = 8)	Unpaired t-test	Two-tailed	*0.25212*	*t = -1.195*
	Slc12a5: eGRASP+ T-E (*n* = 8) vs. eGRASP+ E-E (*n* = 8)	Unpaired t-test	Two-tailed	*0.44468*	*t =-0.787*
	Unc13a: eGRASP+ T-E (*n* = 8) vs. eGRASP+ E-E (*n* = 7)	Unpaired t-test	Two-tailed	*0.48378*	*t =-0.721*
	Dbn1: eGRASP+ T-E (*n* = 8) vs. eGRASP+ E-E (*n* = 8)	Unpaired t-test	Two-tailed	*0.62403*	*t =0.501*
	Dlg4: eGRASP+ T-E (*n* = 8) vs. eGRASP+ E-E (*n* = 7)	Unpaired t-test	Two-tailed	*0.70808*	*t = -0.383*
	Dlg3: eGRASP+ T-E (*n* = 8) vs. eGRASP+ E-E (*n* = 7)	Unpaired t-test	Two-tailed	*0.71561*	*t =0.372*
	Gnao1: eGRASP+ T-E (*n* = 8) vs. eGRASP+ E-E (*n* = 7)	Unpaired t-test	Two-tailed	*0.82589*	*t =0.224*
**7G**	$CA3^E$-$CA1^E$ vs. $CA3^T$-$CA1^E$, *n*-animals = 5, *n*-dendrites = 47, *n*-spines $CA3^T$-$CA1^E$ = 2245, *n*-spines $CA3^E$ -$CA1^E$ = 1388	Hierarchical generalized linear mixed model with Bonferroni corrected post-hoc comparisons		*0.0327*(p_{adj})	*ratio = 0.942, z = -2.546*
	$CA3^N$-$CA1^E$ vs. $CA3^T$-$CA1^E$, *n*-animals = 5, *n*-dendrites = 47, *n*-spines $CA3^T$-$CA1^E$ = 2245, *n*-spines $CA3^{NE}$ -$CA1^E$ = 857	Hierarchical generalized linear mixed model with Bonferroni corrected post-hoc comparisons		*0.0004* (p_{adj})	*ratio = 1.110, z = 3.792*
	$CA3^E$-$CA1^E$ vs. $CA3^N$-$CA1^E$, *n*-animals = 5, *n*-dendrites = 47, *n*-spines $CA3^{NE}$ -$CA1^E$ = 857, *n*-spines $CA3^E$-$CA1^E$ = 1388	Hierarchical generalized linear mixed model with Bonferroni corrected post-hoc comparisons		*< 0.0001* (p_{adj})	*ratio = 1.178, z = 5.471*
**7H**	$CA3^E$-$CA1^E$ vs. $CA3^T$-$CA1^E$, *n*-animals = 5, *n*-dendrites = 47, *n*-spines $CA3^T$-$CA1^E$ = 2245, *n*-spines $CA3^E$ -$CA1^E$ = 1388	Hierarchical generalized linear mixed model with Bonferroni corrected post-hoc comparisons		*0.0002* (p_{adj})	*ratio = -0.018, z = -3.956*
	$CA3^N$-$CA1^E$ vs. $CA3^T$-$CA1^E$, n-animals = 5, *n*-dendrites = 47, *n*-spines $CA3^T$-$CA1^E$ = 2245, *n*-spines $CA3^{NE}$ -$CA1^E$ = 857	Hierarchical generalized linear mixed model with Bonferroni corrected post-hoc comparisons		*< 0.0001* (p_{adj})	*ratio = 0.0298, z = 5.438*
	$CA3^E$-$CA1^E$ vs. $CA3^{NE}$-$CA1^E$, *n*-animals = 5, *n*-dendrites = 47, *n*-spines $CA3^{NE}$ -$CA1^E$ = 857, *n*-spines $CA3^E$-$CA1^E$ = 1388	Hierarchical generalized linear mixed model with Bonferroni corrected post-hoc comparisons		*< 0.0001* (p_{adj})	*ratio = 0.047, z = 8.087*
**7J**	$CA3^E$-$CA1^E$ vs. $CA3^T$-$CA1^E$, *n*-animals = 5, *n*-dendrites = 47, *n*-spines $CA3^T$-$CA1^E$ = 846, *n*-spines $CA3^E$ -$CA1^E$ = 551	Hierarchical generalized linear mixed model with Bonferroni corrected post-hoc comparisons		*0.561* (p_{adj})	*estimate = 0.204 t =1.321*

	$CA3^{NE}$-$CA1^{E}$ vs. $CA3^{T}$-$CA1^{E}$, *n*-animals = 5, *n*-dendrites = 47, *n*-spines $CA3^{T}$-$CA1^{E}$ = 846, *n*-spines $CA3^{NE}$ -$CA1^{E}$ = 295	Hierarchical generalized linear mixed model with Bonferroni corrected post-hoc comparisons		*1.000 (p_{adj})*	*estimate = -0.109*<br>*t =-0.877*
	$CA3^{E}$-$CA1^{E}$ vs. $CA3^{NE}$-$CA1^{E}$, *n*-animals = 5, *n*-dendrites = 47, *n*-spines $CA3^{NE}$ -$CA1^{E}$ = 295, *n*-spines $CA3^{E}$-$CA1^{E}$ = 551	Hierarchical generalized linear mixed model with Bonferroni corrected post-hoc comparisons		*0.178 (p_{adj})*	*estimate = 0.314*<br>*t =1.887*
**7K**	$CA3^{E}$-$CA1^{E}$ vs. $CA3^{T}$-$CA1^{E}$, *n*-animals = 5, *n*-dendrites = 47, *n*-spines $CA3^{T}$-$CA1^{E}$ = 846, *n*-spines $CA3^{E}$ -$CA1^{E}$ = 551	Hierarchical generalized linear mixed model with Bonferroni corrected post-hoc comparisons		*1.000 (p_{adj})*	*estimate = 0.008*<br>*t =0.306*
	$CA3^{NE}$-$CA1^{E}$ vs. $CA3^{T}$-$CA1^{E}$, *n*-animals = 5, *n*-dendrites = 47, *n*-spines $CA3^{T}$-$CA1^{E}$ = 846, *n*-spines $CA3^{NE}$ -$CA1^{E}$ = 295	Hierarchical generalized linear mixed model with Bonferroni corrected post-hoc comparisons		*1.000 (p_{adj})*	*estimate = -0.004*<br>*t =-0.204*
	$CA3^{E}$-$CA1^{E}$ vs. $CA3^{NE}$-$CA1^{E}$, *n*-animals = 5, *n*-dendrites = 47, *n*-spines $CA3^{NE}$ -$CA1^{E}$ = 295, *n*-spines $CA3^{E}$-$CA1^{E}$ = 551	Hierarchical generalized linear mixed model with Bonferroni corrected post-hoc comparisons		*1.000 (p_{adj})*	*estimate = 0.012*<br>*t =0.437*
**7L**	YFP+CFC+ vs. YFP+<br>*n*-animals = 5, *n*-dendrites = 47, *n*-spines = 2245	Hierarchical mixed-effect logistic regression with Bonferroni corrected post-hoc comparisons		*0.684*	*OR = 1.091,*<br>*CI = (0.917-1.300)*
	YFP+CFC- vs. YFP+<br>*n*-animals = 5, *n*-dendrites = 47, *n*-spines = 2245	Hierarchical mixed-effect logistic regression with Bonferroni corrected post-hoc comparisons		*0.283*	*OR = 0.864,*<br>*CI = (0.701-1.065)*
	YFP+CFC+ vs. YFP+CFC-<br>*n*-animals = 5, *n*-dendrites = 47, *n*-spines = 2245	Hierarchical mixed-effect logistic regression with Bonferroni corrected post-hoc comparisons		*0.039*	*OR = 1.264,*<br>*CI = (1.008-1.585)*
**S3G**	eGRASP- FSC vs. eGRASP+ FSC, *n* = 3	Wilcoxon signed rank test	Two-tailed	*0.5*	*W=-4*
**S3H**	eGRASP- SSC vs. eGRASP+ SSC, *n* = 3	Wilcoxon signed rank test	Two-tailed	*0.5*	*W=-4*
**S5G**	CFC GRASP+ Non-SynGO vs. CFCGRASP+ SynGO	Mann Whitney test	Two-tailed	*<0.0001*	*W=112500626*
	CFC GRASP- Non-SynGO vs. CFC GRASP- SynGO	Mann Whitney test	Two-tailed	*<0.0001*	*W=115819973*
	CE GRASP+ Non-SynGO vs. CE GRASP+ SynGO	Mann Whitney test	Two-tailed	*<0.0001*	*W=141881399*
	CE GRASP- Non-SynGO vs. CE GRASP- SynGO	Mann Whitney test	Two-tailed	*<0.0001*	*W=147154865*
	CFC GRASP+ SynGO vs. CFC GRASP- SynGO	Kruskal Wallis test,<br>Dunn's multiple comparisons test	Two-tailed	*>0.9999 (p_{adj})*	*z = 0.008812*
	CFC GRASP+ SynGO vs. CE GRASP+ SynGO	Kruskal Wallis test,<br>Dunn's multiple comparisons test	Two-tailed	*>0.9999 (p_{adj})*	*z = 0.6425*

	CFC GRASP- SynGO vs. CE GRASP+ SynGO	Kruskal Wallis test, Dunn's multiple comparisons test	Two-tailed	>0.9999 (p_{adj})	$z = 0.6322$
	CFC GRASP- SynGO vs. CE GRASP- SynGO	Kruskal Wallis test, Dunn's multiple comparisons test	Two-tailed	0.1497 (p_{adj})	$z = 2.785$
	CE GRASP+ SynGO vs. CE GRASP- SynGO	Kruskal Wallis test, Dunn's multiple comparisons test	Two-tailed	0.7371 (p_{adj})	$z = 2.221$
	CFC GRASP+ SynGO vs. CE GRASP- SynGO	Kruskal Wallis test, Dunn's multiple comparisons test	Two-tailed	0.1433 (p_{adj})	$z = 2.8$
**S10A**	CFC (n = 975) vs. CE (n = 605)	Wilcoxon rank sum test	Two-tailed	0.665	*W=298758*

Supplementary Methods

Immunoblotting

Immunoblotting of subcellular fractions was performed as previously described (*72*). Using a Bradford protein estimation assay reagent (Bio-Rad, The Netherlands), 5 µg of protein from each isolated subcellular fraction (H, M, P2, and SS) was mixed with 5X SDS-containing protein loading buffer. The samples were then boiled at 98 °C for 5 min before being loaded onto an SDS-PAGE gel (4–15%) Criterion TGX Stain-Free Precast Gels (Bio-Rad, The Netherlands). Immediately after SDS-PAGE gel electrophoresis, the gel was scanned using a Gel Doc Easy Scanner (Biorad, The Netherlands), and the images were acquired using the Image Lab software. Subsequently, proteins were transferred onto a PVDF membrane overnight at 4 °C at 40 V using a PowerPac300 (Bio-Rad, The Netherlands). Blocking was performed with 5% non-fat milk in Tris Buffer Saline with Tween-20 (Sigma Aldrich, Netherlands) for 2 h at room temperature. The membrane was then washed three times in TBS with 0.05 % Tween-20. Next, the membranes were incubated overnight with primary antibodies; anti-Syp (1:5000, Guinea pig, cat. 101004, SySy); anti-Dlg4 (1:10000, Mouse, cat. 75-028, Neuromab), anti-vGlut1 (1:5000, Rabbit, cat. 135302, SySy), anti-Homer1 (1:2000, Guinea pig, cat.160004, SySy), anti-Dlg3 (1:1000, Rabbit, cat. PA5-29116, Thermo Fisher Scientific), anti-Gria1 (1:20000, Rabbit, cat. ab109450, Abcam), anti-Grin1 (1:1000, Mouse, cat. 75-272, Neuromab), anti-Gephryn (1:1000, Mouse, cat. 147111, SySy), anti-Glt1 (1:1000, Rabbit, cat. PA5-17099**,** Invitrogen), anti-NeuN (1:1000, Mouse, cat. MAB377, Sigma-Aldrich) in 3 % non-fat milk. Blots were washed three more times in TBS-T, followed by a 2 h incubation with an HRP-conjugated secondary antibodies; Goat anti-Mouse IgG (1:10000, cat. P0447, Agilent Dako), Goat Anti-Rabbit IgG (1:10000, cat. P0448, Agilent Dako), Rabbit Anti-Guinea Pig IgG (1:10000, cat. P0141, Agilent Dako) in 3 % non-fat milk. After incubation, the blots were washed three times and incubated with SuperSignal West Femto Chemiluminescent Substrate (Thermo Fisher Scientific, The Netherlands). Subsequently, blots were scanned on an

Odyssey Fc scanner (LI-COR Biosciences, USA), and the images were acquired using the Image Studio software. To account for gel loading differences, the intensity of bands on the gel was quantified using the Image Studio Lite software (LI-COR Biosciences, U.S.A), and data was used for normalisation with the corresponding band intensity values from the immunoblots. For protein abundance analysis, a heat map was generated by scaling protein signal intensity values between zero and 100 % for a subcellular fraction with the highest protein abundance.

Cell lines and transfection

Neuro2A cells were maintained in culture with DMEM/10%FBS HI/Pen/Strep (Fisher Scientific). Cells were plated in a 96 well plate on the day before and were at 50% confluence on the day of transfection. The cells were co-transfected with 27 ng of DNA per well of pAAV-EWB-DIO-myrTagRFP-T-P2A-post-eGRASP and a Cre-plasmid, and either a pAAV-CWB-yellow pre-eGRASP(p32) or a control vector (pAAV-MCS) using Polyethylenimine (PEI). Media was replaced 24 h later and cells were fixed after 48 h. Nuclei were stained using DAPI and imaged at 40x magnification using an inverted fluorescence microscope (Leica Microsystems, DMi8).

Legends for supplementary data files:

Supplementary Data 1. MS quantified proteins in CFC and their corresponding protein abundance values.

Supplementary Data 2. MS quantified proteins in CE and their corresponding protein abundance values.

Supplementary Data 3. CFC and CE MS quantified proteins (identified in at least 50 % of the eGRASP+ and eGRASP- samples/group) used for differential expression analysis before and after filtering to remove eGRASP derived peptides (related to Fig. 2L, M and supplementary fig. S6, C-F).

Supplementary Data 4. Overlap between proteins identified after CFC and CE and proteins annotated to SynGO defined synaptic subcompartments (related to Fig. 2I). Biological process annotations included.

Supplementary Data 5. Proteins significantly regulated after CFC and CE in eGRASP+ synaptosomes when compared to eGRASP- synaptosomes before and after filtering to remove eGRASP derived peptides (related to Fig. 2L, M and supplementary fig. S6, C-F).

Supplementary Data 6. Functional classes/categories of proteins significantly enriched and depleted after CFC or CE in eGRASP+ synaptosomes when compared to eGRASP- synaptosomes (related to Fig. S8A and Fig. S9A).

Supplementary Data 7. Enriched and depleted Kegg pathways driven by differentially expressed proteins after CFC or CE. Pathways in red are depicted in Fig. S8B and Fig. S9B.

Supplementary Data 8. SynGO annotated proteins significantly enriched and depleted after CFC and CE (related to Fig. 4).

Supplementary Data 9. Overlap between CFC and CE SynGO regulated proteins and known modulators of synaptic plasticity (related to Fig. 5A-D).

Supplementary Data 10. List of proteins used for paralog analysis, and example publications implicating paralogs to various synapse functions, learning & memory, and diversity (related to Fig. 5E).

Supplementary Data 11. Overlap between CFC/CE enriched proteins and proteins dysregulated in the CA1/subiculum of human AD postmortem tissue(29) (related to Fig. S10B).

Supplementary Data 12. GWAS and OMIM analyses (related to Fig. S10, C-E).

Supplementary Data 13. List of proteins used for dominance analysis between CFC and CE, highlighting (red) CFC-dominant and CE-dominant proteins, and their corresponding SynGO-CC location, stats before and after filtering to remove eGRASP derived peptides (related to Fig. 6).

Supplementary Data 14. PheWAS analysis of dominant proteins after CFC and CE.

Supplementary Source data. Source data for Fig. 1, fig. 2, C, E and F, fig. 3, fig. 7, D, F and G-L, fig. S4B, fig. S5, A and C and fig. S10.

REFERENCES

1. S. Tonegawa, X. Liu, S. Ramirez, R. Redondo, Memory engram cells have come of age. *Neuron* **87**, 918–931 (2015).

2. D. J. Cai, D. Aharoni, T. Shuman, J. Shobe, J. Biane, W. Song, B. Wei, M. Veshkini, M. La-Vu, J. Lou, S. E. Flores, I. Kim, Y. Sano, M. Zhou, K. Baumgaertel, A. Lavi, M. Kamata, M. Tuszynski, M. Mayford, P. Golshani, A. J. Silva, A shared neural ensemble links distinct contextual memories encoded close in time. *Nature* **534**, 115–118 (2016).

3. X. Liu, S. Ramirez, P. T. Pang, C. B. Puryear, A. Govindarajan, K. Deisseroth, S. Tonegawa, Optogenetic stimulation of a hippocampal engram activates fear memory recall. *Nature* **484**, 381–385 (2012).

4. N. Matsuo, L. Reijmers, M. Mayford, Spine-type-specific recruitment of newly synthesized AMPA receptors with learning. *Science* **319**, 1104–1107 (2008).

5. P. Rao-Ruiz, J. J. Couey, I. M. Marcelo, C. G. Bouwkamp, D. E. Slump, M. R. Matos, R. J. van der Loo, G. J. Martins, M. van den Hout, W. F. van IJcken, R. M. Costa, M. C. van den Oever, S. A. Kushner, Engram-specific transcriptome profiling of contextual memory consolidation. *Nat. Commun.* **10**, 2232 (2019).

6. D. S. Roy, Y. G. Park, M. E. Kim, Y. Zhang, S. K. Ogawa, N. DiNapoli, X. Gu, J. H. Cho, H. Choi, L. Kamentsky, J. Martin, O. Mosto, T. Aida, K. Chung, S. Tonegawa, Brain-wide mapping reveals that engrams for a single memory are distributed across multiple brain regions. *Nat. Commun.* **13**, 1799 (2022).

7. K. K. Tayler, K. Z. Tanaka, L. G. Reijmers, B. J. Wiltgen, Reactivation of neural ensembles during the retrieval of recent and remote memory. *Curr. Biol.* **23**, 99–106 (2013).

8. R. P. Kesner, Behavioral functions of the CA3 subregion of the hippocampus. *Learn. Mem.* **14**, 771–781 (2007).

9. Y. Shinohara, K. Kohara, Projections of hippocampal CA2 pyramidal neurons: Distinct innervation patterns of CA2 compared to CA3 in rodents. *Hippocampus* **33**, 691–699 (2023).

10. J. H. Choi, S. E. Sim, J. I. Kim, D. I. Choi, J. Oh, S. Ye, J. Lee, T. Kim, H. G. Ko, C. S. Lim, B. K. Kaang, Interregional synaptic maps among engram cells underlie memory formation. *Science* **360**, 430–435 (2018).

11. C. Lee, B. H. Lee, H. Jung, C. Lee, Y. Sung, H. Kim, J. Kim, J. Y. Shim, J. I. Kim, D. I. Choi, H. Y. Park, B. K. Kaang, Hippocampal engram networks for fear memory recruit new synapses and modify pre-existing synapses in vivo. *Curr. Biol.* **33**, 507–516.e3 (2023).

12. T. J. Ryan, D. S. Roy, M. Pignatelli, A. Arons, S. Tonegawa, Memory., Engram cells retain memory under retrograde amnesia. *Science* **348**, 1007–1013 (2015).

13. P. Rao-Ruiz, E. Visser, M. Mitric, A. B. Smit, M. C. van den Oever, A synaptic framework for the persistence of memory engrams. *Front. Synaptic Neurosci.* **13**, 661476 (2021).

14. C. Ortega-de San Luis, T. J. Ryan, Understanding the physical basis of memory: Molecular mechanisms of the engram. *J. Biol. Chem.* **298**, 101866 (2022).

15. T. Kim, D. I. Choi, J. E. Choi, H. Lee, H. Jung, J. Kim, Y. Sung, H. Park, M. J. Kim, D. H. Han, S. H. Lee, B. K. Kaang, Activated somatostatin interneurons orchestrate memory microcircuits. *Neuron* **112**, 201–208.e4 (2024).

16. D. I. Choi, J. Kim, H. Lee, J. I. Kim, Y. Sung, J. E. Choi, S. J. Venkat, P. Park, H. Jung, B. K. Kaang, Synaptic correlates of associative fear memory in the lateral amygdala. *Neuron* **109**, 2717–2726.e3 (2021).

17. E. Thanou, F. Koopmans, D. Pita-Illobre, R. V. Klaassen, B. Ozer, I. Charalampopoulos, A. B. Smit, K. W. Li, Suspension TRAPping filter (sTRAP) sample preparation for quantitative proteomics in the low µg input range using a plasmid DNA micro-spin column: Analysis of the hippocampus from the 5xFAD Alzheimer's Disease Mouse Model. *Cells* **12**, 1242 (2023).

18. V. Demichev, C. B. Messner, S. I. Vernardis, K. S. Lilley, M. Ralser, DIA-NN: Neural networks and interference correction enable deep proteome coverage in high throughput. *Nat. Methods* **17**, 41–44 (2020).

19. F. J. Rubio, D. E. Olivares, C. Dunn, S. Zhang, E. M. Hilaire, A. Henry, C. Mejias-Aponte, C. J. Nogueras-Ortiz, P. V. Selvam, F. C. Cruz, R. Madangopal, M. Morales, B. T. Hope, Flow cytometry of synaptoneurosomes (FCS) reveals increased ribosomal S6 and calcineurin proteins in activated medial prefrontal cortex to nucleus accumbens synapses. *J. Neurosci.* **43**, 4217–4233 (2023).

20. J. Kim, T. Zhao, R. S. Petralia, Y. Yu, H. Peng, E. Myers, J. C. Magee, mGRASP enables mapping mammalian synaptic connectivity with light microscopy. *Nat. Methods* **9**, 96–102 (2011).

21. E. H. Feinberg, M. K. Vanhoven, A. Bendesky, G. Wang, R. D. Fetter, K. Shen, C. I. Bargmann, GFP Reconstitution Across Synaptic Partners (GRASP) defines cell contacts and synapses in living nervous systems. *Neuron* **57**, 353–363 (2008).

22. M. R. Matos, E. Visser, I. Kramvis, R. J. van der Loo, T. Gebuis, R. Zalm, P. Rao-Ruiz, H. D. Mansvelder, A. B. Smit, M. C. van den Oever, Memory strength gates the involvement of a CREB-dependent cortical fear engram in remote memory. *Nat. Commun.* **10**, 2315 (2019).

23. E. Visser, M. R. Matos, R. J. van der Loo, N. J. Marchant, T. J. de Vries, A. B. Smit, M. C. van den Oever, A persistent alcohol cue memory trace drives relapse to alcohol seeking after prolonged abstinence. *Sci. Adv.* **6**, eaax7060 (2020).

24. S. L. Lesuis, N. Brosens, N. Immerzeel, R. J. van der Loo, M. Mitric, P. Bielefeld, C. P. Fitzsimons, P. J. Lucassen, S. A. Kushner, M. C. van den Oever, H. J. Krugers, Glucocorticoids promote fear generalization by increasing the size of a dentate gyrus engram cell population. *Biol. Psychiatry* **90**, 494–504 (2021).

25. C. Biesemann, M. Gronborg, E. Luquet, S. P. Wichert, V. Bernard, S. R. Bungers, B. Cooper, F. Varoqueaux, L. Li, J. A. Byrne, H. Urlaub, O. Jahn, N. Brose, E. Herzog, Proteomic screening of glutamatergic mouse brain synaptosomes isolated by fluorescence activated sorting. *EMBO J.* **33**, 157–170 (2014).

26. V. Paget-Blanc, M. E. Pfeffer, M. Pronot, P. Lapios, M. F. Angelo, R. Walle, F. P. Cordelieres, F. Levet, S. Claverol, S. Lacomme, M. Petrel, C. Martin, V. Pitard, V. De Smedt Peyrusse, T.

Biederer, D. Perrais, P. Trifilieff, E. Herzog, A synaptomic analysis reveals dopamine hub synapses in the mouse striatum. *Nat. Commun.* **13**, 3102 (2022).

27. F. Koopmans, P. van Nierop, M. Andres-Alonso, A. Byrnes, T. Cijsouw, M. P. Coba, L. N. Cornelisse, R. J. Farrell, H. L. Goldschmidt, D. P. Howrigan, N. K. Hussain, C. Imig, A. P. H. de Jong, H. Jung, M. Kohansalnodehi, B. Kramarz, N. Lipstein, R. C. Lovering, H. MacGillavry, V. Mariano, H. Mi, M. Ninov, D. Osumi-Sutherland, R. Pielot, K. H. Smalla, H. Tang, K. Tashman, R. F. G. Toonen, C. Verpelli, R. Reig-Viader, K. Watanabe, J. van Weering, T. Achsel, G. Ashrafi, N. Asi, T. C. Brown, P. De Camilli, M. Feuermann, R. E. Foulger, P. Gaudet, A. Joglekar, A. Kanellopoulos, R. Malenka, R. A. Nicoll, C. Pulido, J. de Juan-Sanz, M. Sheng, T. C. Sudhof, H. U. Tilgner, C. Bagni, A. Bayes, T. Biederer, N. Brose, J. J. E. Chua, D. C. Dieterich, E. D. Gundelfinger, C. Hoogenraad, R. L. Huganir, R. Jahn, P. S. Kaeser, E. Kim, M. R. Kreutz, P. S. McPherson, B. M. Neale, V. O'Connor, D. Posthuma, T. A. Ryan, C. Sala, G. Feng, S. E. Hyman, P. D. Thomas, A. B. Smit, M. Verhage, SynGO: An evidence-based, expert-curated knowledge base for the synapse. *Neuron* **103**, 217–234.e4 (2019).

28. N. G. Skene, S. G. Grant, Identification of vulnerable cell types in major brain disorders using single cell transcriptomes and expression weighted cell type enrichment. *Front. Neurosci.* **10**, 16 (2016).

29. D. Di Fraia, M. Anitei, M. T. Mackmull, L. Parca, L. Behrendt, A. Andres-Pons, D. Gilmour, M. Helmer Citterich, C. Kaether, M. Beck, A. Ori, Conserved exchange of paralog proteins during neuronal differentiation. *Life Sci. Alliance* **5**, e202201397 (2022).

30. J. Nithianantharajah, N. H. Komiyama, A. McKechanie, M. Johnstone, D. H. Blackwood, D. St Clair, R. D. Emes, L. N. van de Lagemaat, L. M. Saksida, T. J. Bussey, S. G. Grant, Synaptic scaffold evolution generated components of vertebrate cognitive complexity. *Nat. Neurosci.* **16**, 16–24 (2013).

31. S. Meftah, J. Gan, Alzheimer's disease as a synaptopathy: Evidence for dysfunction of synapses during disease progression. *Front. Synaptic Neurosci.* **15**, 1129036 (2023).

32. D. C. Hondius, P. van Nierop, K. W. Li, J. J. Hoozemans, R. C. van der Schors, E. S. van Haastert, S. M. van der Vies, A. J. Rozemuller, A. B. Smit, Profiling the human hippocampal proteome at all pathologic stages of Alzheimer's disease. *Alzheimers Dement.* **12**, 654–668 (2016).

33. D. I. Choi, B. K. Kaang, Interrogating structural plasticity among synaptic engrams. *Curr. Opin. Neurobiol.* **75**, 102552 (2022).

34. P. Nemat, S. Semenova, R. J. van der Loo, A. B. Smit, S. Spijker, M. C. van den Oever, P. Rao-Ruiz, Structural synaptic signatures of contextual memory retrieval-reactivated hippocampal engram cells. *Neurobiol. Learn. Mem.* **218**, 108033 (2025).

35. L. J. Goeminne, K. Gevaert, L. Clement, Peptide-level robust ridge regression improves estimation, sensitivity, and specificity in data-dependent quantitative label-free shotgun proteomics. *Mol. Cell. Proteomics* **15**, 657–668 (2016).

36. A. Sticker, L. Goeminne, L. Martens, L. Clement, Robust summarization and inference in proteome-wide label-free quantification. *Mol. Cell. Proteomics* **19**, 1209–1219 (2020).

37. P. Y. Wu, L. Ji, C. De Sanctis, A. Francesconi, Y. Inglebert, R. A. McKinney, Loss of synaptopodin impairs mGluR5 and protein synthesis-dependent mGluR-LTD at CA3-CA1 synapses. *PNAS Nexus* **3**, pgae062 (2024).

38. M. van Oostrum, E. M. Schuman, Understanding the molecular diversity of synapses. *Nat. Rev. Neurosci.* **26**, 65–81 (2025).

39. M. Rigby, F. W. Grillo, B. Compans, G. Neves, J. Gallinaro, S. Nashashibi, S. Horton, P. M. Pereira Machado, M. A. Carbajal, G. Vizcay-Barrena, F. Levet, J. B. Sibarita, A. Kirkland, R. A. Fleck, C. Clopath, J. Burrone, Multi-synaptic boutons are a feature of CA1 hippocampal connections in the stratum oriens. *Cell Rep.* **42**, 112397 (2023).

40. M. S. Kleinjan, W. C. Buchta, R. Ogelman, I. W. Hwang, M. Kuwajima, D. D. Hubbard, D. J. Kareemo, O. Prikhodko, S. L. Olah, L. E. Gomez Wulschner, W. C. Abraham, S. J. Franco, K. M. Harris, W. Chan Oh, M. J. Kennedy, Dually innervated dendritic spines develop in the

absence of excitatory activity and resist plasticity through tonic inhibitory crosstalk. *Neuron* **111**, 1517 (2023).

41. G. W. Knott, C. Quairiaux, C. Genoud, E. Welker, Formation of dendritic spines with GABAergic synapses induced by whisker stimulation in adult mice. *Neuron* **34**, 265–273 (2002).

42. Y. Geinisman, R. W. Berry, J. F. Disterhoft, J. M. Power, E. A. Van der Zee, Associative learning elicits the formation of multiple-synapse boutons. *J. Neurosci.* **21**, 5568–5573 (2001).

43. M. Uytiepo, Y. Zhu, E. Bushong, K. Chou, F. S. Polli, E. Zhao, K. Y. Kim, D. Luu, L. Chang, D. Yang, T. C. Ma, M. Kim, Y. Zhang, G. Walton, T. Quach, M. Haberl, L. Patapoutian, A. Shahbazi, Y. Zhang, E. Beutter, W. Zhang, B. Dong, A. Khoury, A. Gu, E. McCue, L. Stowers, M. Ellisman, A. Maximov, Synaptic architecture of a memory engram in the mouse hippocampus. *Science* **387**, eado8316 (2025).

44. J. M. Veres, T. Andrasi, P. Nagy-Pal, N. Hajos, CaMKIIα promoter-controlled circuit manipulations target both pyramidal cells and inhibitory interneurons in cortical networks. *eNeuro* **10**, ENEURO.0070-23.2023 (2023).

45. D. Jeon, Y. M. Yang, M. J. Jeong, K. D. Philipson, H. Rhim, H. S. Shin, Enhanced learning and memory in mice lacking Na+/Ca2+ exchanger 2. *Neuron* **38**, 965–976 (2003).

46. G. H. Diering, R. L. Huganir, The AMPA receptor code of synaptic plasticity. *Neuron* **100**, 314–329 (2018).

47. S. Neyman, D. Manahan-Vaughan, Metabotropic glutamate receptor 1 (mGluR1) and 5 (mGluR5) regulate late phases of LTP and LTD in the hippocampal CA1 region in vitro. *Eur. J. Neurosci.* **27**, 1345–1352 (2008).

48. R. Fujii, S. Okabe, T. Urushido, K. Inoue, A. Yoshimura, T. Tachibana, T. Nishikawa, G. G. Hicks, T. Takumi, The RNA binding protein TLS is translocated to dendritic spines by mGluR5 activation and regulates spine morphology. *Curr. Biol.* **15**, 587–593 (2005).

49. S. Daumas, H. Halley, B. Frances, J. M. Lassalle, Encoding, consolidation, and retrieval of contextual memory: Differential involvement of dorsal CA3 and CA1 hippocampal subregions. *Learn. Mem.* **12**, 375–382 (2005).

50. K. Tully, V. Y. Bolshakov, Emotional enhancement of memory: How norepinephrine enables synaptic plasticity. *Mol. Brain* **3**, 15 (2010).

51. M. Lovett-Barron, P. Kaifosh, M. A. Kheirbek, N. Danielson, J. D. Zaremba, T. R. Reardon, G. F. Turi, R. Hen, B. V. Zemelman, A. Losonczy, Dendritic inhibition in the hippocampus supports fear learning. *Science* **343**, 857–863 (2014).

52. N. Brosens, S. L. Lesuis, P. Rao-Ruiz, M. C. van den Oever, H. J. Krugers, Shaping memories via stress: A synaptic engram perspective. *Biol. Psychiatry* **95**, 721–731 (2024).

53. M. Shpokayte, O. McKissick, X. Guan, B. Yuan, B. Rahsepar, F. R. Fernandez, E. Ruesch, S. L. Grella, J. A. White, X. S. Liu, S. Ramirez, Hippocampal cells segregate positive and negative engrams. *Commun. Biol.* **5**, 1009 (2022).

54. R. L. Redondo, J. Kim, A. L. Arons, S. Ramirez, X. Liu, S. Tonegawa, Bidirectional switch of the valence associated with a hippocampal contextual memory engram. *Nature* **513**, 426–430 (2014).

55. J. T. Lambert, T. C. Hill, D. K. Park, J. H. Culp, K. Zito, Protracted and asynchronous accumulation of PSD95-family MAGUKs during maturation of nascent dendritic spines. *Dev. Neurobiol.* **77**, 1161–1174 (2017).

56. S. Reddy-Alla, M. A. Bohme, E. Reynolds, C. Beis, A. T. Grasskamp, M. M. Mampell, M. Maglione, M. Jusyte, U. Rey, H. Babikir, A. W. McCarthy, C. Quentin, T. Matkovic, D. D. Bergeron, Z. Mushtaq, F. Gottfert, D. Owald, T. Mielke, S. W. Hell, S. J. Sigrist, A. M. Walter, Stable positioning of Unc13 restricts synaptic vesicle fusion to defined release sites to promote synchronous neurotransmission. *Neuron* **95**, 1350–1364.e12 (2017).

57. M. A. Bohme, A. W. McCarthy, A. T. Grasskamp, C. B. Beuschel, P. Goel, M. Jusyte, D. Laber, S. Huang, U. Rey, A. G. Petzoldt, M. Lehmann, F. Gottfert, P. Haghighi, S. W. Hell, D.

Owald, D. Dickman, S. J. Sigrist, A. M. Walter, Rapid active zone remodeling consolidates presynaptic potentiation. *Nat. Commun.* **10**, 1085 (2019).

58. H. Sakamoto, T. Ariyoshi, N. Kimpara, K. Sugao, I. Taiko, K. Takikawa, D. Asanuma, S. Namiki, K. Hirose, Synaptic weight set by Munc13-1 supramolecular assemblies. *Nat. Neurosci.* **21**, 41–49 (2018).

59. F. Zhu, M. Cizeron, Z. Qiu, R. Benavides-Piccione, M. V. Kopanitsa, N. G. Skene, B. Koniaris, J. DeFelipe, E. Fransen, N. H. Komiyama, S. G. N. Grant, Architecture of the mouse brain synaptome. *Neuron* **99**, 781–799.e10 (2018).

60. F. Varoqueaux, A. Sigler, J. S. Rhee, N. Brose, C. Enk, K. Reim, C. Rosenmund, Total arrest of spontaneous and evoked synaptic transmission but normal synaptogenesis in the absence of Munc13-mediated vesicle priming. *Proc. Natl. Acad. Sci. U.S.A.* **99**, 9037–9042 (2002).

61. A. Pooryasin, M. Maglione, M. Schubert, T. Matkovic-Rachid, S. M. Hasheminasab, U. Pech, A. Fiala, T. Mielke, S. J. Sigrist, Unc13A and Unc13B contribute to the decoding of distinct sensory information in Drosophila. *Nat. Commun.* **12**, 1932 (2021).

62. C. M. Nievergelt, A. X. Maihofer, E. G. Atkinson, C. Y. Chen, K. W. Choi, J. R. Coleman, N. P. Daskalakis, L. E. Duncan, R. Polimanti, C. Aaronson, A. B. Amstadter, S. B. Andersen, O. A. Andreassen, P. A. Arbisi, A. E. Ashley-Koch, S. B. Austin, E. Avdibegovic, D. Babic, S. A. Bacanu, D. G. Baker, A. Batzler, J. C. Beckham, S. Belangero, C. Benjet, C. Bergner, L. M. Bierer, J. M. Biernacka, L. J. Bierut, J. I. Bisson, M. P. Boks, E. A. Bolger, A. Brandolino, G. Breen, R. A. Bressan, R. A. Bryant, A. C. Bustamante, J. Bybjerg-Grauholm, M. Baekvad-Hansen, A. D. Borglum, S. Borte, L. Cahn, J. R. Calabrese, J. M. Caldas-de-Almeida, C. Chatzinakos, S. Cheema, S. A. P. Clouston, L. Colodro-Conde, B. J. Coombes, C. S. Cruz-Fuentes, A. M. Dale, S. Dalvie, L. K. Davis, J. Deckert, D. L. Delahanty, M. F. Dennis, T. deRoon-Cassini, F. Desarnaud, C. P. DiPietro, S. G. Disner, A. R. Docherty, K. Domschke, G. Dyb, A. D. Kulenovic, H. J. Edenberg, A. Evans, C. Fabbri, N. Fani, L. A. Farrer, A. Feder, N. C. Feeny, J. D. Flory, D. Forbes, C. E. Franz, S. Galea, M. E. Garrett, B. Gelaye, J. Gelernter, E. Geuze, C. F. Gillespie, A. Goci, S. B. Goleva, S. D. Gordon, L. R. Grasser, C. Guindalini, M. Haas, S. Hagenaars, M. A. Hauser, A. C. Heath, S. M. Hemmings, V. Hesselbrock, I. B. Hickie, K. Hogan, D. M. Hougaard, H. Huang, L. M. Huckins, K. Hveem, M.

Jakovljevic, A. Javanbakht, G. D. Jenkins, J. Johnson, I. Jones, T. Jovanovic, K. I. Karstoft, M. L. Kaufman, J. L. Kennedy, R. C. Kessler, A. Khan, N. A. Kimbrel, A. P. King, N. Koen, R. Kotov, H. R. Kranzler, K. Krebs, W. S. Kremen, P. F. Kuan, B. R. Lawford, L. A. M. Lebois, K. Lehto, D. F. Levey, C. Lewis, I. Liberzon, S. D. Linnstaedt, M. W. Logue, A. Lori, Y. Lu, B. J. Luft, M. K. Lupton, J. J. Luykx, I. Makotkine, J. L. Maples-Keller, S. Marchese, C. Marmar, N. G. Martin, G. A. MartInez-Levy, K. McAloney, A. McFarlane, K. A. McLaughlin, S. A. McLean, S. E. Medland, D. Mehta, J. Meyers, V. Michopoulos, E. A. Mikita, L. Milani, W. Milberg, M. W. Miller, R. A. Morey, C. P. Morris, O. Mors, P. B. Mortensen, M. S. Mufford, E. C. Nelson, M. Nordentoft, S. B. Norman, N. R. Nugent, M. O'Donnell, H. K. Orcutt, P. M. Pan, M. S. Panizzon, G. A. Pathak, E. S. Peters, A. L. Peterson, M. Peverill, R. H. Pietrzak, M. A. Polusny, B. Porjesz, A. Powers, X. J. Qin, A. Ratanatharathorn, V. B. Risbrough, A. L. Roberts, B. O. Rothbaum, A. O. Rothbaum, P. Roy-Byrne, K. J. Ruggiero, A. Rung, H. Runz, B. P. F. Rutten, S. S. de Viteri, G. A. Salum, L. Sampson, S. E. Sanchez, M. Santoro, C. Seah, S. Seedat, J. S. Seng, A. Shabalin, C. M. Sheerin, D. Silove, A. K. Smith, J. W. Smoller, S. R. Sponheim, D. J. Stein, S. Stensland, J. S. Stevens, J. A. Sumner, M. H. Teicher, W. K. Thompson, A. K. Tiwari, E. Trapido, M. Uddin, R. J. Ursano, U. Valdimarsdottir, L. L. van den Heuvel, M. Van Hooff, S. J. van Rooij, E. Vermetten, C. H. Vinkers, J. Voisey, Z. Wang, Y. Wang, M. Waszczuk, H. Weber, F. R. Wendt, T. Werge, M. A. Williams, D. E. Williamson, B. S. Winsvold, S. Winternitz, E. J. Wolf, C. Wolf, Y. Xia, Y. Xiong, R. Yehuda, R. M. Young, K. A. Young, C. C. Zai, G. C. Zai, M. Zervas, H. Zhao, L. A. Zoellner, J. A. Zwart, M. B. Stein, K. J. Ressler, K. C. Koenen, Discovery of 95 PTSD loci provides insight into genetic architecture and neurobiology of trauma and stress-related disorders. medRxiv (2023); https://doi.org/10.1101/2023.08.31.23294915.

63. A. Kreis, F. Issa, X. Yerna, C. Jabbour, O. Schakman, M. de Clippele, N. Tajeddine, N. Pierrot, J. N. Octave, R. Gualdani, P. Gailly, Conditional deletion of KCC2 impairs synaptic plasticity and both spatial and nonspatial memory. *Front. Mol. Neurosci.* **16**, 1081657 (2023).

64. B. D. Hobson, P. A. Sims, Critical analysis of particle detection artifacts in synaptosome flow cytometry. *eNeuro* **6**, ENEURO.0009-19.2019 (2019).

65. P. Rao-Ruiz, J. Yu, S. A. Kushner, S. A. Josselyn, Neuronal competition: Microcircuit mechanisms define the sparsity of the engram. *Curr. Opin. Neurobiol.* **54**, 163–170 (2019).

66. N. Apostolo, S. N. Smukowski, J. Vanderlinden, G. Condomitti, V. Rybakin, J. Ten Bos, L. Trobiani, S. Portegies, K. M. Vennekens, N. V. Gounko, D. Comoletti, K. D. Wierda, J. N. Savas, J. de Wit, Synapse type-specific proteomic dissection identifies IgSF8 as a hippocampal CA3 microcircuit organizer. *Nat. Commun.* **11**, 5171 (2020).

67. T. Kitamura, S. K. Ogawa, D. S. Roy, T. Okuyama, M. D. Morrissey, L. M. Smith, R. L. Redondo, S. Tonegawa, Engrams and circuits crucial for systems consolidation of a memory. *Science* **356**, 73–78 (2017).

68. S. Heo, G. H. Diering, C. H. Na, R. S. Nirujogi, J. L. Bachman, A. Pandey, R. L. Huganir, Identification of long-lived synaptic proteins by proteomic analysis of synaptosome protein turnover. *Proc. Natl. Acad. Sci. U.S.A.* **115**, E3827–E3836 (2018).

69. J. Schindelin, I. Arganda-Carreras, E. Frise, V. Kaynig, M. Longair, T. Pietzsch, S. Preibisch, C. Rueden, S. Saalfeld, B. Schmid, J. Y. Tinevez, D. J. White, V. Hartenstein, K. Eliceiri, P. Tomancak, A. Cardona, Fiji: An open-source platform for biological-image analysis. *Nat. Methods* **9**, 676–682 (2012).

70. E. Aarts, M. Verhage, J. V. Veenvliet, C. V. Dolan, S. van der Sluis, A solution to dependency: Using multilevel analysis to accommodate nested data. *Nat. Neurosci.* **17**, 491–496 (2014).

71. J. J. Hox, M. Moerbeek, R. van de Schoot, Multilevel Analysis (Routledge, ed. 2, 2010).

72. N. J. Pandya, F. Koopmans, J. A. Slotman, I. Paliukhovich, A. B. Houtsmuller, A. B. Smit, K. W. Li, Correlation profiling of brain sub-cellular proteomes reveals co-assembly of synaptic proteins and subcellular distribution. *Sci. Rep.* **7**, 12107 (2017).

73. K. H. Gylys, J. A. Fein, F. Yang, D. J. Wiley, C. A. Miller, G. M. Cole, Synaptic changes in Alzheimer's disease: Increased amyloid-beta and gliosis in surviving terminals is accompanied by decreased PSD-95 fluorescence. *Am. J. Pathol.* **165**, 1809–1817 (2004).

74. K. N. Richter, H. Wildhagen, M. S. Helm, J. E. Ussling, T. Schikorski, S. O. Rizzoli, Comparative synaptosome imaging: A semi-quantitative method to obtain copy numbers for synaptic and neuronal proteins. *Sci. Rep.* **8**, 14838 (2018).

75. B. A. Gyorffy, J. Kun, G. Torok, E. Bulyaki, Z. Borhegyi, P. Gulyassy, V. Kis, P. Szocsics, A. Micsonai, J. Matko, L. Drahos, G. Juhasz, K. A. Kekesi, J. Kardos, Local apoptotic-like mechanisms underlie complement-mediated synaptic pruning. *Proc. Natl. Acad. Sci. U.S.A.* **115**, 6303–6308 (2018).

76. M. Marcatti, A. Fracassi, M. Montalbano, C. Natarajan, B. Krishnan, R. Kayed, G. Taglialatela, Aβ/tau oligomer interplay at human synapses supports shifting therapeutic targets for Alzheimer's disease. *Cell. Mol. Life Sci.* **79**, 222 (2022).

77. S. Sokolow, K. M. Henkins, I. A. Williams, H. V. Vinters, I. Schmid, G. M. Cole, K. H. Gylys, Isolation of synaptic terminals from Alzheimer's disease cortex. *Cytometry A* **81A**, 248–254 (2012).

78. T. W. van Voorst, M. A. van Boven, K. I. Marinus, J. M. Colon-Mercado, J. Schretzmeir, C. Haag, R. F. Toonen, F. Koopmans, M. E. Ward, A. B. Smit, R. E. van Kesteren, M. Verhage, L. N. Cornelisse, One-step induction of human GABAergic neurons promotes presynaptic development & synapse maturation. bioRxiv 662293 (2025); https://doi.org/10.1101/2025.06.30.662293.

79. P. Skowronek, M. Thielert, E. Voytik, M. C. Tanzer, F. M. Hansen, S. Willems, O. Karayel, A. D. Brunner, F. Meier, M. Mann, Rapid and in-depth coverage of the (phospho-)proteome with deep libraries and optimal window design for dia-PASEF. *Mol. Cell. Proteomics* **21**, 100279 (2022).

80. F. Koopmans, K. W. Li, R. V. Klaassen, A. B. Smit, MS-DAP platform for downstream data analysis of label-free proteomics uncovers optimal workflows in benchmark data sets and increased sensitivity in analysis of Alzheimer's biomarker data. *J. Proteome Res.* **22**, 374–386 (2023).

81. Z. Yao, C. T. J. van Velthoven, T. N. Nguyen, J. Goldy, A. E. Sedeno-Cortes, F. Baftizadeh, D. Bertagnolli, T. Casper, M. Chiang, K. Crichton, S. L. Ding, O. Fong, E. Garren, A. Glandon, N. W. Gouwens, J. Gray, L. T. Graybuck, M. J. Hawrylycz, D. Hirschstein, M. Kroll, K. Lathia, C. Lee, B. Levi, D. McMillen, S. Mok, T. Pham, Q. Ren, C. Rimorin, N. Shapovalova, J. Sulc, S. M. Sunkin, M. Tieu, A. Torkelson, H. Tung, K. Ward, N. Dee, K. A. Smith, B. Tasic, H. Zeng, A taxonomy of transcriptomic cell types across the isocortex and hippocampal formation. *Cell* **184**, 3222–3241.e26 (2021).

82. P. D. Thomas, D. Ebert, A. Muruganujan, T. Mushayahama, L. P. Albou, H. Mi, PANTHER: Making genome-scale phylogenetics accessible to all. *Protein Sci.* **31**, 8–22 (2022).

83. S. X. Ge, D. Jung, R. Yao, ShinyGO: A graphical gene-set enrichment tool for animals and plants. *Bioinformatics* **36**, 2628–2629 (2020).

84. M. Kanehisa, M. Furumichi, Y. Sato, M. Ishiguro-Watanabe, M. Tanabe, KEGG: Integrating viruses and cellular organisms. *Nucleic Acids Res.* **49**, D545–D551 (2021).

85. K. Watanabe, S. Stringer, O. Frei, M. Umicevic Mirkov, C. de Leeuw, T. J. C. Polderman, S. van der Sluis, O. A. Andreassen, B. M. Neale, D. Posthuma, A global overview of pleiotropy and genetic architecture in complex traits. *Nat. Genet.* **51**, 1339–1348 (2019).

86. G. Stelzer, I. Plaschkes, D. Oz-Levi, A. Alkelai, T. Olender, S. Zimmerman, M. Twik, F. Belinky, S. Fishilevich, R. Nudel, Y. Guan-Golan, D. Warshawsky, D. Dahary, A. Kohn, Y. Mazor, S. Kaplan, T. Iny Stein, H. N. Baris, N. Rappaport, M. Safran, D. Lancet, VarElect: The phenotype-based variation prioritizer of the GeneCards Suite. *BMC Genomics* **17**, 444 (2016).

87. D. Szklarczyk, A. L. Gable, K. C. Nastou, D. Lyon, R. Kirsch, S. Pyysalo, N. T. Doncheva, M. Legeay, T. Fang, P. Bork, L. J. Jensen, C. von Mering, The STRING database in 2021: Customizable protein-protein networks, and functional characterization of user-uploaded gene/measurement sets. *Nucleic Acids Res.* **49**, D605–D612 (2021).

88. Y. Perez-Riverol, A. Csordas, J. Bai, M. Bernal-Llinares, S. Hewapathirana, D. J. Kundu, A. Inuganti, J. Griss, G. Mayer, M. Eisenacher, E. Perez, J. Uszkoreit, J. Pfeuffer, T. Sachsenberg, S. Yilmaz, S. Tiwary, J. Cox, E. Audain, M. Walzer, A. F. Jarnuczak, T. Ternent,

A. Brazma, J. A. Vizcaino, The PRIDE database and related tools and resources in 2019: Improving support for quantification data. *Nucleic Acids Res.* **47**, D442–D450 (2019).

89. Allen Institute for Brain Science, Allen Mouse Brain Atlas [dataset] (2004); https://atlas.brain-map.org/.
